# The species-level microbiota of healthy eyes revealed by the integration of metataxonomics with culturomics and genome analysis

**DOI:** 10.3389/fmicb.2022.950591

**Published:** 2022-09-02

**Authors:** Kui Dong, Ji Pu, Jing Yang, Guohong Zhou, Xuan Ji, Zhiming Kang, Juan Li, Min Yuan, Xiaoling Ning, Zhaoxia Zhang, XingYu Ma, Yanpeng Cheng, Hong Li, Qin Ma, Hong Li, Lijun Zhao, Wenjing Lei, Bin Sun, Jianguo Xu

**Affiliations:** ^1^Shanxi Eye Hospital, Shanxi Province Key Laboratory of Ophthalmology, Taiyuan, China; ^2^School of Public Health, Shanxi Medical University, Taiyuan, China; ^3^State Key Laboratory of Infectious Disease Prevention and Control, National Institute for Communicable Disease Control and Prevention, Chinese Center for Disease Control and Prevention, Beijing, China; ^4^Shenzhen Center for Disease Control and Prevention, Shenzhen, China; ^5^Research Institute of Public Heath, Nankai University, Tianjin, China; ^6^Research Units of Discovery of Unknown Bacteria and Function, Chinese Academy of Medical Sciences, Beijing, China

**Keywords:** species-level microbiota, ocular surface, healthy eye, metataxonomics, antibiotics resistance, 16S rRNA, culturomics

## Abstract

**Objectives:**

To characterize the healthy ocular surface microbiota at the species level, including cultured and uncultured taxa.

**Methods:**

We integrated the metataxonomic method with culturomics and genome sequencing analysis of selected isolated strains to better illustrate the taxonomic structure of the ocular surface microbiota. The metataxonomics used the full-length 16S rRNA gene sequences and the operational phylogenetic unit strategy, which can precisely identify the cultured and uncultured or potentially new taxa to species level based on the phylogenetic tree constructed.

**Results:**

We detected 1,731 operational phylogenetic units (OPUs) in 196 healthy eyes from 128 people, affiliated to 796 cultured species, 784 potentially new species, and 151 potentially new higher taxa. The microbiota for each eye had 49.17 ± 35.66 OPUs. Of the 796 cultured species, 170 (21.36%) had previously caused clinical infections. Based on where they were initially isolated, the ocular surface microbiota mainly came from human body sites (34.55%), the environment (36.93%), plants (9.05%), animals (4.90%), and others; 428 strains were isolated from 20 eyes, affiliated to 42 species, and had come from the environment (33.33%) and the skin (16.67%). Of these, 47.62% had previously caused clinical infections. Genome analysis of 73 isolators revealed that 68.5% of them carried antibiotic resistance genes. The most frequently isolated genera, namely *Staphylococcus, Streptococcus*, and *Moraxella*, had an average of 5.30, four, and three resistance genes per strain, respectively.

**Discussion:**

The study found that the ocular surface microbiota mainly came from the environment, plants, animals, food, and human body sites such as the skin, oral cavity, upper respiratory tract, etc. No core member of ocular surface microbiota was detected at the species level. The human eyes were invaded and colonized by bacteria from the exposed environment, some of which were capable of causing infections in humans and carried antibiotic resistance genes. Preventive measures should be developed to protect our eyes from danger.

## Introduction

The ocular surface is the interface between the eye and the environment; it is routinely exposed to the environment and interacts with various microorganisms, including pathogens (Zegans and Van Gelder, 2014). The ocular surface microbiota is defined as the microbial species residing on the conjunctiva, associated with the host defense (Kugadas and Gadjeva, 2016). Analyzing the human ocular surface microbiota remained a challenging task because of the difficulty of culturing and detection (St Leger and Caspi, 2018). The total numbers of quantifiable bacterial cells in the conjunctiva are several orders of magnitude less than those found in higher biomass areas like the intestines. The supporting evidence for the existence of core ocular surface microbiota was initially provided by conventional microbial cultivation studies in 1930 (Graham et al., 2007). However, the cultured species might only represent a small proportion of the microbiota which were prone to grow under the applied cultivation conditions (Graham et al., 2007). The advanced high-throughput sequencing studies suggested some core microbiota of the ocular surface, composed of 12 bacterial genera (Graham et al., 2007; Dong et al., 2011). However, the limitations of using a short length of the 16S rRNA sequencing approach are its inability to identify the uncultured bacteria or potentially new bacterial species down to the species level (Meng et al., 2017; Yang et al., 2020), which may magnify the microbiota similarity. The accurate classification of all new bacterial species requires the phylogenetic analysis of the full-length 16S rRNA sequences. It is a requirement for the publication of the description of new bacterial species in the International Journal of Systematic and Evolution of Microbiology (IJSEM).

The reliable and accurate identification of the cultured and uncultured bacteria to species level requires full-length 16S rRNA sequences (Yarza et al., 2014). In this regard, metataxonomics is the method of choice: it uses the almost full-length 16S rRNA gene sequences, generated by the PacBio system (Meng et al., 2017). It relies on the operational phylogenetic unit (OPU) strategy, rather than operational taxonomic units (OTUs). The OPU strategy clusters the full-length 16S rRNA gene sequences at a higher identity threshold and precisely identifies its taxonomic position based on the phylogenetic tree constructed. When the representative sequences of a given OPU were identical or nearly identical (>98.7% identity) with the type strain sequences, it was identified as the cultured species which has been officially recognized. When a given OPU represents an independent lineage within a clear genus, it was assigned as a potentially new species in the given genus. When the OPUs represents an unclear genus, family, or higher taxon, it was proposed to belong to a potential higher taxon, since it cannot be correctly classified based on 16S rRNA sequence only. Both potentially new species and potentially higher taxa have not been cultured and characterized yet (Bai et al., 2018; Wirth et al., 2020). Therefore, this is an accurate approach to species-level classification, especially for uncultured or potentially new bacteria (Wirth et al., 2020). With this strategy, we characterized the intestinal microbiota of Chinese people at the species level and found that a majority of them were uncultured (Meng et al., 2017; Bai et al., 2018; Yang et al., 2020). Herein, we analyze the healthy human ocular surface microbiota at the species level by using the methods of metataxonomics, integrated with culturomics and genome sequencing for selected isolates. We found that the human ocular surface microbiota profile was extremely diverse, with little or no similarity.

## Materials and methods

### Subjects

Volunteers from Taiyuan city of Shanxi Province China who applied *via* the Internet for the study were interviewed and selected by our research team members in 2019. The 128 healthy individuals aged 0–89 were divided into four groups, namely, the children group (under 19 years old), youth group (from 20 to 39), middle age group (from 40 to 59), and elderly group (over 60) (Table 1). The refractive eye was not considered because of its high prevalence. None of the volunteers had signs of disease or abnormal clinical laboratory results. Clinical and ophthalmic examinations included body temperature (36–37°C), pulse (60–100 beats/min), blood pressure (below 140/90 mmHg), respiration, visual acuity, corrected visual acuity, intraocular pressure (10–21 mmHg), eyelid, lacrimal apparatus, conjunctiva (without redness, swelling, hyperemia, increased secretion, and malformation), and protrusion (12–14 mm). The exclusion criteria included severe systemic diseases; pregnant or breastfeeding women; systemic antibiotics or topical eye drops containing antibiotics, anti-inflammatory, and corticosteroid eye drop administration within 3 months; wearing of contact lenses; ocular and periocular infection in the past 4 weeks; eye surgery in the past 3 months; as well as dry eyes and other eye diseases. Dry eye disease was diagnosed by ophthalmologists (Herrero-Vanrell and Peral, 2007; Committee of Cross-Straits Medicine Exchange Association Dry Eye Group of Chinese Ophthalmologist Association, 2021). The study was approved by the Medical Ethics Committee of The Shanxi Provincial Eye Hospital (201804A).

**Table 1:** Statistical table of demographic characteristics of the sample.

**Demographic characteristics**		**Level/category**	**Age**	**Number**	**Percentage (%)**
Gender	Female		42.92 ± 19.15	77	60.16%
	Male		35.69 ± 18.61	51	39.84%
Age group	Children group	Under the age of 9	7.67 ± 1.22	9	7.03%
		10–19 old	13.17 ± 3.19	6	4.70%
	Youth group	20–29 old	25.27 ± 2.15	33	25.78%
		30–39 old	34.16 ± 2.57	19	14.84%
	Middle-aged group	40–49 old	44.54 ± 3.57	13	10.16%
		50–59 old	53.40 ± 2.77	25	19.53%
	Elderly group	60–69 old	64.93 ± 3.24	15	11.72%
		70–79 old	72.80 ± 2.05	5	3.90%
		Over 80 years old	80.67 ± 1.15	3	2.34%

Conjunctival swabs were sampled at the same hospital by professional nurses. Three minutes after instilling topical anesthesia (proparacaine hydrochloride ophthalmic solution, about 0.039 ml), a sterile swab was applied three times from the medial to the lateral side of the inferior fornix of the conjunctival sac without touching the eyelids, cornea and meibomian, which may decrease the detected ocular microbiota diversity (Shin et al., 2016). The swabs were then conserved in lysis solution and stored in a freezer (at −80°C) until use (Qi et al., 2021).

### Metataxonomic analysis

The detailed metataxonomics procedures have been described in our previous studies (Meng et al., 2017; Yang et al., 2020). Total DNA was lysed and extracted from each swab using the DNeasy PowerSoil^®^ DNA Extraction Kit (Qiagen, Hilden, Germany). The almost full length of the bacterial 16S rRNA gene was amplified using the universal primer set 27F (5′-AGAGTTTGATCCTGGCTCAG-3′) and 1492R (5′-TACGACTTAACCCCAATCGC-3′). PCR was performed using KAPA HiFi high-fidelity polymerase (Kapa Biosystems, USA). The parameters for amplification were as follows: an initial denaturation step at 95°C for 3 min; then 35 cycles at 95°C for 20 s, 64°C for 15 s, and 72°C for 100 s; and finally a 100-s extension at 72°C. The amplicons were purified using the QIA Rapid PCR Purification Kit (Qiagen, Hilden, Germany). Integrity was then determined using an Agilent 2100 Bioanalyzer (Agilent, USA) and agarose gel electrophoresis, and purity and concentration were checked using a Nanodrop 2000 (Thermo Fisher, Shanghai, China). Library preparation and sequencing were performed at Tianjin BioChip (Tianjin, China) according to the standard procedure of PacBio Sequel, using P6/C4 chemistry and a 20 h sequencing chip. Raw sequencing data were processed based on PacBio SMRT Link (version 6.0.0) software. The downstream sequencing data of multiple samples were split according to Sequal_384_ barcodes with a minimum barcode score of 26. To reduce the error rate and improve the sequence quality, filtering was followed with a minimum number of five circular consensus sequencing (CCS) cycles and a minimum prediction accuracy of 99.9%. Subsequently, QIIME software was applied to remove ambiguous bases, low-quality sequences, and sequencing primers. Sequences exceeding the expected size (<1,200 and >1,600 bp) were trimmed. Chimeras were detected and removed using the UCHIME algorithm embedded in the USEARCH software (version 11.0.667; option:-UCHIME_ ref-strand plus-non-chimeras) (Meng et al., 2017; Yang et al., 2020). The microbiome raw sequence data of this study have been deposited in the NCBI Sequence Read Archive (SRA) with the accession code PRJNA741708 (SRR17068294~ SRR17068494).

The USEARCH pipeline was used to cluster the 16S rRNA sequences into OTUs with a threshold set at 98.7% identity. The most frequent reads of each OTU were selected as a representative to be added to the LTP132 database (The All-Species Living Tree Project) and aligned using the SINA tool (SILVA Incremental Aligner). The aligned sequences were inserted into the default tree using the Parsimony tool implemented in the ARB software package. The resulting insertions were manually inspected to recognize all representative sequences affiliating closely with either type strain sequences or clearly within a genus lineage. All sequences that were unaffiliated were added to the SILVA SSURef_NR_132 (Silva Reference Non-Redundant) database and inserted into the default tree. Approximately three closest relative sequences representing uncultured organisms were selected for each independent lineage made by the OTU representatives and exported to the LTP132 database. A subset of sequences containing (i) all PacBio OTU representative sequences, (ii) the selection of the reference type strains and the SILVA REF132 recruited sequences, and (iii) the neighbor-joining supporting sequence data set was used to reconstruct a neighbor-joining tree. The reconstruction was performed using a 30% conservational filter to avoid phylogenetic noise. All OPUs were generated individually by the manual inspection of the constructed NJ tree.

### The relative abundance and prevalence of eye OPUs

Analysis for the relative abundance of a given OPU in each sample was normalized as OPU reads/total reads. The prevalence of OPUs was determined by plotting the number and relative abundance of OPUs in 5% intervals (from 0 to 100% of the samples). The resulting OPU table was divided according to different prevalence levels, and their proportions were analyzed separately. Visualization of OPU distribution was conducted by package *circlize* implemented in R software (version 4.0.0) (Yang et al., 2020).

### Culturomics

Twenty eye samples were selected for the culturomics study (Dubourg et al., 2013, 2014). The brain heart infusion agar (BHIA), BHIA with 5% defibrillated sheep blood, R-2A Agar(R_2_A), trypticase soy agar medium (TSA), columbia agar (CA), and nutrient agar (NA) medium were used to culture the bacterial cells at 35 or 37°C, anaerobically or aerobically. In order to obtain more culturable species, we placed samples on different kinds of plates and inoculated them by streaking. The purified strains were identified by using full-length 16S rRNA gene sequencing.

### The initially isolated origin, antibiotics resistance gene, and infection history

By searching the LPSN (List of Prokaryotic names with Standing in Nomenclature) and NCBI (National Center for Biotechnology Information) databases, the initially isolated origin and infection history of known species in high-throughput sequencing research results were obtained, and the search results are recorded in the attached Supplementary Table 1, and the retrieval source marked. By integrating the CARD and ResFinder databases, drug resistance genes were annotated on the genome of isolated strains (the similarity threshold was set at 80% and the coverage threshold was set at 70%).

### Information analysis of ocular surface microbiota

The isolation origin of the isolated or named species detected by metataxonomics was mainly collected from the papers published in the International Journal of Systematic Bacteriology, the official publication of the International Committee on Systematics of Prokaryotes, where they were initially isolated. The genome of the selected strains was sequenced for potential virulence and antibiotic resistance genes (Liu and Pop, 2009). All named species were searched in the published literature for clinical infection records.

## Results

### Hierarchic taxonomic composition of the ocular surface microbiota

A total of 302 volunteers signed up to participate in this study through public recruitment. After the strict screening, and after excluding potential volunteers with eye diseases and systemic diseases, a total of 128 volunteers were enrolled and conjunctival sac swab samples were collected from them. The average age of the 128 volunteers was 40.03 years; the oldest was 82 years old and the youngest was 6 years old. There were 51 males (39.84%) and 77 females (60.16%), and by age group, there were 15 teenagers (11.73%), 52 (40.62%) in the youth group, 38 (29.69%) in the middle-aged group, and 23 (17.96%) in the elderly group (Table 1). The 128 healthy individuals aged 0–89 were divided into four groups, namely, the children group (under 19 years old), youth group (from 20 to 39), middle age group (from 40 to 59), and elderly group (over 60) (Table 1). We obtained 256 conjunctival swabs from the 128 volunteers; 196 swabs yielded high-quality sequencing data, which fulfilled our quality control (Johnson et al., 2019). We obtained both left and right healthy eye swabs from 67 volunteers. Analysis of the sequencing data of subjects with both left and right eyes sampled at the same time showed no significant difference in Shannon index and Chao's estimator (Pairwise Wilcoxon test, Shannon index -*p*value = 0.60, Chao's estimator *p*-value = 0.36) (Supplementary Figure 1).

The PacBio sequencing platform rendered 1,973,971 raw 16S rRNA reads from the 196 conjunctival swabs. After quality filtering and chimera removal, 1,209,465 (61.27%) high-quality reads of 16S rRNA amplicons were obtained, with an average of 6,017 ± 4,434 reads per swab and an average of 1,451 ± 31 base pairs (bp) in length per reading (Supplementary Table 2). Using the USEARCH pipeline, 1,209,465 almost full-length 16S rRNA reads were clustered into 15,049 OTUs at 98.7% identity, the threshold for the discrimination of species. Of these, 639 OTUs were removed as contamination reads because they were detected from the commercial laboratory materials used for sampling and sequencing. The most frequent representative sequences within each OTU were selected for phylogenetic inference using the LTP132 (Yarza et al., 2010) or the NR database (Quast et al., 2013). Thereafter, 1,731 OPUs were identified based on the visual inspection of the final tree, generated using database-based phylogenetic *de novo* tree reconstruction (Mora-Ruiz Mdel et al., 2015; Vidal et al., 2015; Meng et al., 2017). Rarefaction curve analysis showed high coverage but incomplete saturation (Supplementary Figure 2). The average error rate generated by the PacBio sequencing platform was 0.011%. The full-length 16S rRNA sequences and conservational filters were used to reconstruct the trees *de novo* (Yarza et al., 2014).

**Figure 1:**
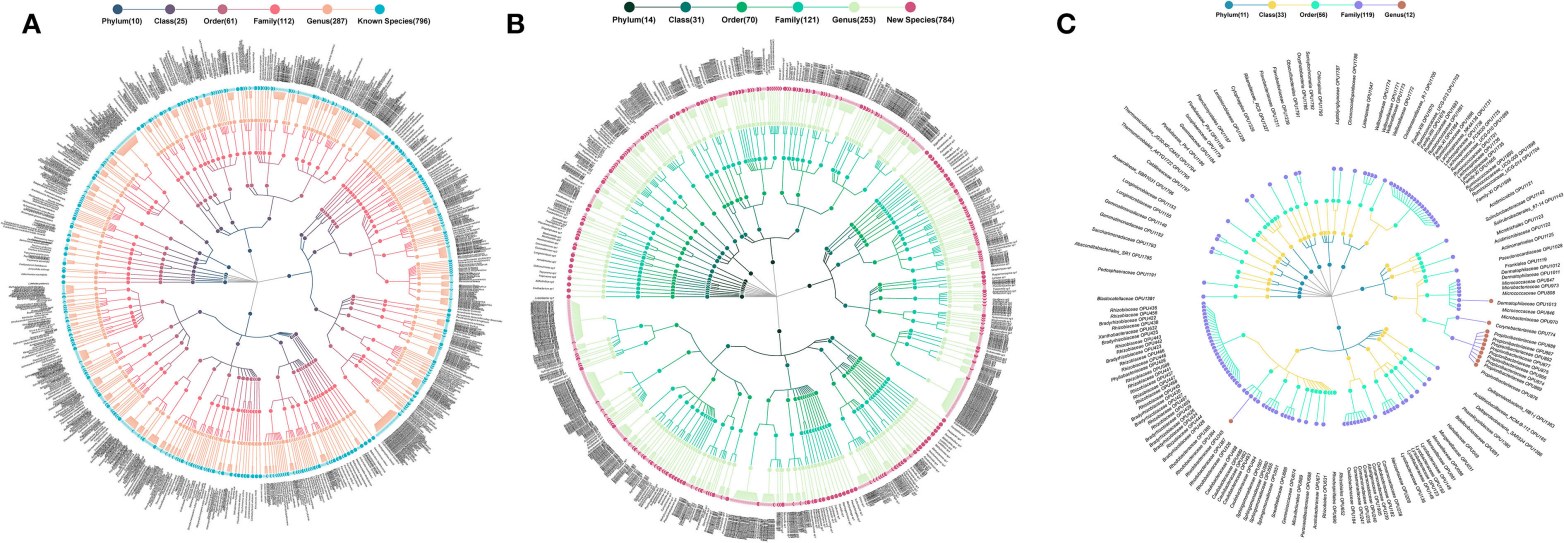
Taxonomic profiles of human ocular surface microbiota. **(A)** 796 cultured species, **(B)** 784 potentially new species, and **(C)** 151 potentially higher taxa. *Each dot represents an OPU. The descending hierarchical levels are expressed from the inner to the outer rings. The total number of OPUs at different hierarchical levels is displayed in brackets.

The 1,731 OPUs were further identified as 796 cultured, 784 potentially new species, and 151 potentially higher taxa (Meng et al., 2017; Bai et al., 2018) (Figure 1). The potentially new species proposed in this study were suggested by full-length 16S rRNA sequence analysis, of which the phylogenetic position was established, but has not been isolated and characterized yet. According to bacterial taxonomy rules, the similarity of the full-length 16S rRNA of the potentially new species with all validated species in the given genus was <98.7% and could form independent branches on the phylogenetic tree constructed according to all the 16S rRNA mentioned above (Chun et al., 2018).

The 796 cultured species, listed in the officially validated bacterial species (Yarza et al., 2014), were affiliated to 115 genera, 62 families, 27 orders, 12 classes, and 5 phyla, accounting for 44.66% of the total reads (Figure 1A). The 784 potentially new species were affiliated to 107 genera, 68 families, 36 orders, 24 classes, and 14 phyla, accounting for 40.84% of the total reads (Figure 1B). The remaining 151 OPUs were affiliated with 119 families, 56 orders, 33 classes, and 14 phyla, accounting for 14.70% of the total reads (Figure 1C).

### The prevalence and relative abundances of OPUs of the ocular surface microbiota

**Figure 2:**
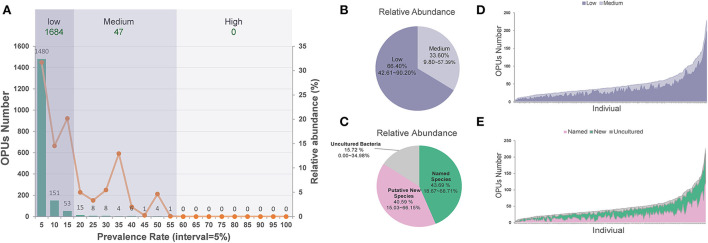
The distribution of low and medium prevalent 1,731 OPUs in the healthy eyes. **(A)** The prevalence and relative abundance of OPUs in ocular surface microbiota the numbers (left axis) and the relative abundance (right axis) of the OPUs in individuals with the 5% interval. **(B)** The relative abundance of OPUs in low and medium prevalent group. **(C)** The relative abundance of OPUs of named species, potential new species, and high-level taxa. **(D)** The number of OPUs in low and medium prevalence groups in 196 eyes. **(E)** The number of OPUs of named, potential species, and high-level taxa in 196 eyes.

On average, each ocular surface microbiota had 49.17 ± 35.66 OPUs, for the 196 healthy eyes studied. Only one of the 1,731 OPUs was distributed in 55% of the eyes. None was detected in over 60% of the eyes. Therefore, no core microbiota exists in the eye at the species level (Figure 2A).

Of the 1,731 OPUs, 1,480 (85.5%) were distributed in <5% of the 196 eyes, while 151 (8.7%) and 53 (3.1%) were detected in <10 or <15% of the eyes. Only five (0.29%) OPUs were detected in 50–55% of healthy eyes. Based on this observation, we classified the 1,684 (97.28%) of 1,731 OPUs as an extremely low-prevalence bacteria group, distributed in <15% of the 196 eyes, accounting for 66.40% of the total reads (Figure 2B). We classified the 47 (2.72%) OPUs into the medium-prevalence bacteria group, accounting for 33.60% of the total reads (Figure 2).

By analyzing the OPUs from 67 volunteers who provided swabs of both eyes, no significant difference in alpha diversity (Shannon index) and several species (Chao's estimator) was observed (data are not shown).

### The culturomics analysis of the ocular surface microbiota

There were 428 strains isolated from 20 healthy eye swabs, affiliated with 4 phyla, 8 classes, 13 orders, 20 families, 22 genera, and 42 species; 375 (87.6%) isolated strains were distributed in <15% of the eyes; none was detected in over 55% of the eyes (Supplementary Figure 3). The most frequently isolated bacterial species were *Staphylococcus epidermidis* (*n* = 168, 39.3%), *Microbacterium oleivorans* (*n* = 30, 7.0%), *Brevundimonas vesicularis* (*n* = 28, 6.5%), *Bacillus pumilus* (*n* = 26, 6.1%), *Moraxella osloensis* (*n* = 25, 5.8%), *Staphylococcus haemolyticus* (*n* = 23, 5.4%), and *Staphylococcus warneri* (*n* = 20, 4.6%) (Dubourg et al., 2013, 2014).

### The potential pathogen, antibiotics resistance gene analysis, and resistance test

Of the 796 classified species detected by metataxonomics, 170 (21.36%) had caused clinical infections; this was demonstrated by searching 515 published literature in the NCBI database (Supplementary Table 1). Of the 42 isolated species from 20 eyes, 20 (47.62%) had been shown to cause clinical infection in 35 published literature (Supplementary Table 3, namely, *S. aureus, S. capitis, S. epidermidis, S. haemolyticus, S. pseudopneumoniae*, and *S. oralis*.

**Figure 3:**
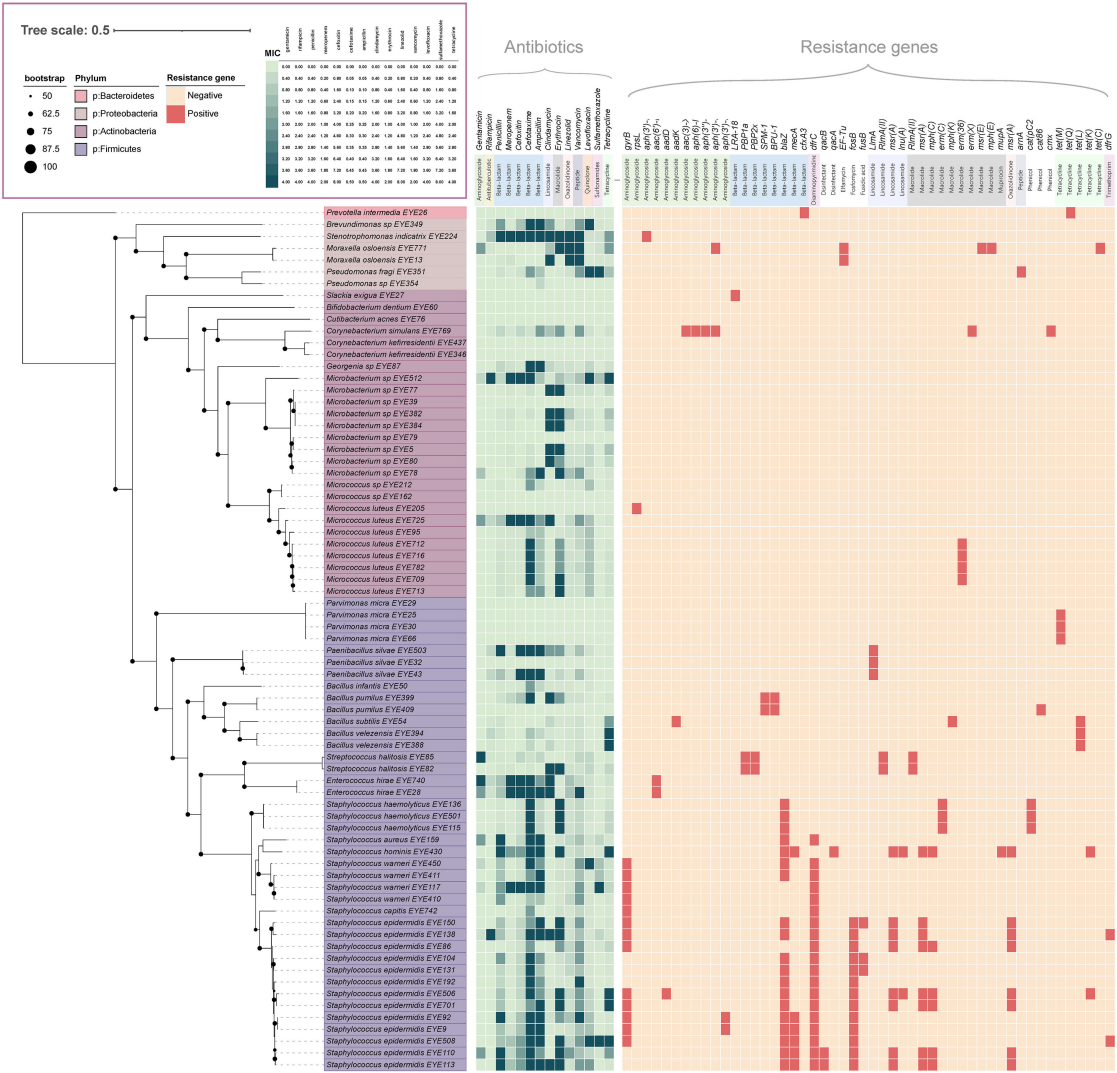
Antibiotic resistance profile of ocular surface isolates. The phylogenetic tree was constructed from concatenated protein sequences using PhyloPhlAn and drawn using iTOL. Strains assigned to various phyla are displayed in different colors. All shades of green pixels represent MIC values. All red pixels represent antimicrobial resistance genes detected along each strain using RGI and ResFinder database.

Of the 428 isolated strains, 73 were selected for genome sequencing, including 24 named species and 5 potentially new species; 43 genes encoding resistance to 12 antibiotics, such as aminoglycosides, beta-lactams, diaminopyrimidine, elfamycin, fosfomycin, fusidic acid, lincosamide, macrolides, mupirocin, oxazolidinone, tetracyclines, and trimethoprim, were detected in those genomes; 50 (68.5%) of the 73 strains carried resistance genes (Liu and Pop, 2009). The genera carrying the most resistance genes were *Staphylococcus, Streptococcus*, and *Moraxella*, with an average of 5.30, four, and three resistance genes per strain, respectively. The most frequently detected resistance genes included *gyrB, blaZ, dfrC, fosB, msr(A)*, and *mph(C)* (Figure 3). Antibiotic resistance tests showed that those strains were resistant to all the 14 antibiotics tested (Figure 3).

### OPU distribution was different in the four age groups

According to the results of the OPU distribution analysis of the four age groups (Supplementary Figure 4), there are 629 OPUs in the children group with an average of 62.10 ± 43.97, 946 OPUs in the youth group with an average of 37.41 ± 22.52, 853 OPUs in the middle-aged group with an average of 46.02 ± 26.04, and 969 OPUs in the elderly group with an average of 74.09 ± 51.98. The average level of the children group and the elderly group in individual OPUs was significantly higher than that of the youth group and the middle-aged group (Supplementary Figure 5). Both the children group and the elderly group had high-priority OPUs (>60%); the children group had four high-priority OPUs: *D. ruhatensis* (80.95%), *S. oralis* subsp. *dentisani* (76.19%)*, S. pitis* (61.90%), and *S. pneumoniae* (61.90%). The elderly group had two highly prevalent OPUs: *Corynebacterium* Sp19 (65.71%) and *P. sphaerophysae* (62.85%).

### Tracing the initially isolated origin

**Figure 4:**
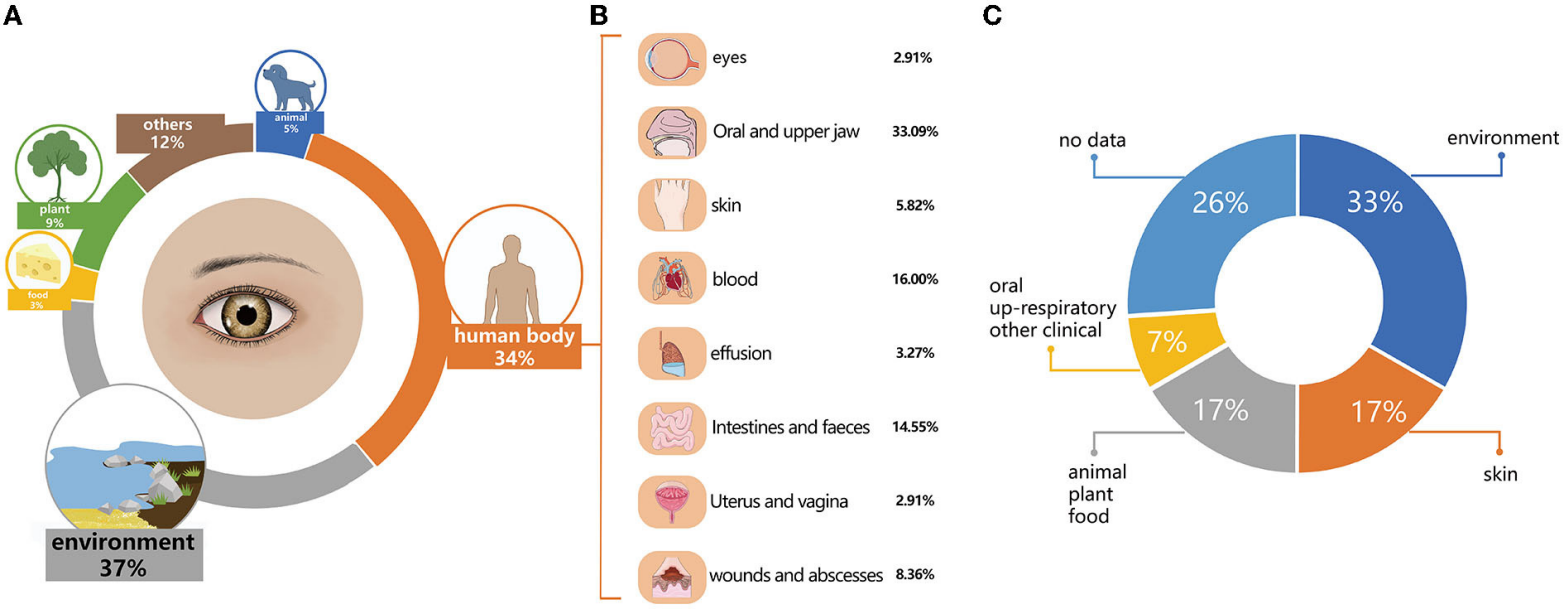
The initially isolated origin and potential pathogens in named species. **(A)** The initial isolation origin of named species detected by metataxonomics. **(B)** The initial isolation origin of named species isolated by culturomics. **(C)** The potential pathogenic species in named species detected by metataxonomic.

Based on where they were initially isolated, the 796 named species detected by metataxonomics can be categorized into environment (*n* = 294, 36.93%), human body sites (*n* = 275, 34.55%), plants (*n* = 72, 9.05%), animals (*n* = 39, 4.90%), food (*n* = 24, 3.02%), and others (*n* = 92, 11.56%). The human body site group was further classified into nine sub-groups, such as oral and upper respiratory tract (*n* = 91, 33.09%), blood (*n* = 44, 16.00%), intestinal and fecal (*n* = 40, 14.55%), infected wounds and abscesses (*n* = 23, 8.36%), skin (*n* = 16, 5.82%), clinical effusion (*n* = 9, 3.27%), eyes (*n* = 8, 2.91%), uterus and vagina (*n* = 8, 2.91%), and other clinical samples (*n* = 36, 13.09%) (Figure 4). The top 20 most prevalent species detected by metataxonomics were mainly from the environment (*D. tsuruhatensis*), skin (*S. capitis, C. acnes)*, plant and others (*P. sphaerophysae, B. vesicularis, E. aerosaccus*), oral and upper respiratory tract (*H. parainfluenzae, S. oralis* subsp. *dentisan*i, S. *pneumoniae, S. oralis* subsp. *oralis., S. sanguinis, C. accolens*), as well as the eye (*G. haemolysans, N. flavescens, P. pasteri*, and *A. oris*).

The 42 isolated species were categorized into four groups. The environment group included 14 species, originating from the air, soil, water, or sludges, such as *A. flavum, B. cereus, B. pumilus, B. velezensis. G. daeguensis, M. oleivorans, M. antarcticus, P. sylvae, P. laeviglucosivorans, P. frederiksbergensis, P. koreensis, P. orientalis, S. olei*, and *T. aidingensis*. The skin group included *C. avidum, S. capitis, S. hominis, S. warneri, S. cristatus, S. haemolyticus*, and *C. acnes*. The animal, plant, and food groups included *A. choanae, A. citreus, E. hirae, P. weihenstephanensis, M. aloeverae, M. endophyticus*, and *M. yunnanensis*. The oral and upper respiratory and other clinical groups included *S. oralis, C. tuberculostearicum*, and *C. simulans*.

## Discussion

The idea of defining the “core microbiota” of shared organisms in a given body habitat of all or the vast majority of humans is intriguing (Turnbaugh et al., 2007). However, a core microbiota may not exist in humans according to a strict definition that requires a particular taxon to be present in each sample; several phyla and genera were found in more than 90% of all samples from either humans or animals (Sekelja et al., 2011; Holman et al., 2017). The existence of a “true” resident of the ocular surface microbiota has been debated for years by ophthalmologists, with no clear consensus reached yet (Zegans and Van Gelder, 2014; Huang et al., 2016; Lyon, 2017). Previous studies with conventional culture methods and high-throughput sequencing technology for the V3–V4 hypervariable region of 16S rRNA suggested a “core” microbiota.

It must be mentioned that analyzing the ocular surface microbiota remained a difficult task because of the sampling difficulty. The conjunctiva is a low biomass tissue. The total numbers of quantifiable bacteria are several orders of magnitude less than those found in other body site specimens such as feces (St Leger and Caspi, 2018). The swab sampling of the conjunctiva only provided a trace amount of specimens for study. It was only enough for culturing or sequencing at most causes. It was also difficult to distinguish if the bacteria were resident in the eyelids or from the conjunctiva. However, with metataxonomic and culturomics methods at a species level, we found that every eye was colonized by a group of very diversified bacterial species, with no core bacterial species identified.

Unexpectedly, the human ocular surface microbiota has more bacterial taxa than the intestines (St Leger and Caspi, 2018). A total of 1,731 OPUs were detected in 196 eyes from 128 individuals, including 796 (46%) cultured species, with 935 (54%) unknown bacterial taxa. However, only 1,235 OPUs were detected from the intestinal samples of 120 healthy people, including 461 cultured species and 774 unknown bacterial taxa (Yang et al., 2020). Generally, each OPU represents one species (Yarza et al., 2014; del Mora-Ruiz et al., 2016). It seems that the human eye interacts with more bacterial taxa than the intestines, taxonomically (Yang et al., 2020).

The human ocular surface microbiota differed extremely among the volunteers. On average, 49.17 ± 35.66 OPUs per eye were detected in 196 eyes from 128 individuals (Supplementary Table 4). For intestine samples, 186 ± 51 OPUs per person were detected in a cohort of 120 individuals (Yang et al., 2020). It seems that the human ocular surface microbiota is more diversified than that of the intestines. Of the 116 OPUs on average for intestinal microbiota per individual detected, 90 OPUs were shared by over 60% of individuals in the cohort, with 77.58% similarity (90/116) (Yang et al., 2020). However, no OPU was detected in over 60% of eyes. Therefore, the microbiota taxonomic similarity of the human eye was very low. This data strongly suggested that the human ocular surface microbiota is to a greater extent personalized, and varied extremely among the volunteers, without core species (Yang et al., 2020). Based on this observation, we hypothesized that the human ocular surface microbiota is mainly from the environments the eyes are exposed to, which could trigger a large immune response (Zegans and Van Gelder, 2014).

In this study, 73 isolated strains were sequenced, and a total of 43 genes encoding resistance to 12 antibiotics were detected. The detection of antibiotic resistance genes of eye isolated strains in this study will help clinicians to understand the changes in the resistance genes of ocular bacteria, which can provide a more reliable basis for the selection of antibiotics for patients with ocular inflammation.

The eyes are routinely exposed to various environments and are endangered by the microorganisms in these environments. Of the 796 cultured species detected by the metataxonomic method, 170 (21.36%) had previously caused clinical infections. Of the 42 cultured bacterial species, 20 (47.62%) had caused clinical infections. Some of the most prevalent species had caused clinical infections (Ranc et al., 2018), namely *S. capitis* (49.23% prevalence) (Wirth et al., 2020), *C. acnes* (47.65%) (Platsidaki and Dessinioti, 2018), *S. pneumoniae* (36.4%) (Regev-Yochay et al., 2004), *S. oralis* subsp. *dentisani* (46.67%) (Ronis et al., 2019), *S. oralis* (29.74%), and *S. sanguinis* (27.18%) (Arredondo et al., 2019). Genome sequence analysis provided evidence that 68.5% of the cultured strains carried antibiotic resistance genes. The most frequently isolated genera, such as *Staphylococcus, Streptococcus*, and *Moraxella*, had 3 to 5.3 resistance genes per strain on average, which may pose potential difficulty for antibiotic treatment for eye bacterial infections.

Studies on the composition and function of microbial communities at the ocular surface have brought new insights and clarified the pathology of ocular surface dynamics in health and disease (Aragona et al., 2021). By using 16S, Li et al. (2019) found that patients with dry eye disease had increased levels of *Bacteroidetes*. The microbiota composition was associated with pterygium and lid laxity (Ozkan et al., 2018). Increased levels of some bacteria, such as *S. pneumoniae* and *H. influenzae*, were associated with trachoma (Hu et al., 2011). Zilliox et al. reported that patients with Stevens–Johnson Syndrome had a high abundance of *Staphylococcus* on ocular surfaces. Patients with lax eyelid syndrome and dry eye disease had a relative abundance of *Corynebacterium*. The reduced microbiome diversity was found in four chronic ocular surface diseases (Zilliox et al., 2020). However, most of those studies classified the bacteria into genera or higher taxonomic levels. The species-level information may lead to a better understanding of the topic.

In short, the ocular surface microbiota of healthy eyes has about 49.17 ± 35.66 species-level bacterial taxa on average, the taxonomic profile of which varied individually. No single bacterial species was shared by over 60% of the healthy eyes analyzed. By studying the initially isolated origins of the 796 and 42 cultured species detected by metataxonomics or culturomics, it was found that members of the ocular surface microbiota were mainly from the environment, plants, animals, food, and human body sites. Therefore, it seems that our eyes are invaded and colonized by the bacteria from the environments they are exposed to. Some of the bacteria were able to cause infections and carried antibiotic resistance genes. Therefore, prevention tools or protocols should be developed to protect our eyes from danger. The regulation of ocular surface microbiota seems to be promising for the diagnosis, prevention, and treatment of ocular disease (Ren et al., 2021, 2022).

## Data availability statement

The datasets presented in this study can be found in online repositories. The names of the repository/repositories and accession number(s) can be found in the article/Supplementary material.

## Author contributions

JX and BS proposed, designed, and supervised this study. KD, JP, GZ, HL (thirteenth author), JY, XN, ZZ, XM, HL (fifteenth author), QM, and WL organized the volunteer recruitment, collected the samples, and performed experiments. JL, MY, and LZ performed the antibiotics resistance tests. JP, KD, XJ, ZK, and YC conducted the data analysis. JX, JP, KD, and BS wrote the manuscript. All authors read the manuscript and approved the manuscript.

## Funding

This work was supported by grants from the National Key R&D Program of China (2019YFC1200501 and 2019YFC1200505), the Research Units of Discovery of Unknown Bacteria and Function, Chinese Academy of Medical Science (2018RU010), the Scientific Research Project of Shanxi Provincial Health Commission (2021XM46), the Natural Science Research Project of Shanxi Provincial Department of Science and Technology (20210302123343), and the Shanxi Provincial Eye Hospital Innovation Fund Project (C201901).

## Conflict of interest

The authors declare that the research was conducted in the absence of any commercial or financial relationships that could be construed as a potential conflict of interest.

## Publisher's note

All claims expressed in this article are solely those of the authors and do not necessarily represent those of their affiliated organizations, or those of the publisher, the editors and the reviewers. Any product that may be evaluated in this article, or claim that may be made by its manufacturer, is not guaranteed or endorsed by the publisher.

## Supplementary material

Supplementary Figure 1 Group differences in alpha diversity at the species level.

Supplementary Figure 2 The rarefaction curves.

Supplementary Figure 3 The distribution and detection rate of cultured strains were isolated.

Supplementary Figure 4 OPU distribution in each aging group.

Supplementary Figure 5 Diversity of species abundance in different age groups.

Supplementary Table 1 Statistical table of pathogenicity analysis of eye surface sequencing bacteria.

Supplementary Table 2 OPUs table.

Supplementary Table 3 Statistical table of pathogenicity analysis of strains isolated and cultivated on the ocular surface.

Supplementary Table 4 Basic information of volunteers.
