# Supplementary figures and images for: The species-level microbiota of healthy eyes revealed by the integration of metataxonomics with culturomics and genome analysis
Source: Front Microbiol. 2022 Sep 2;13:950591. doi: 10.3389/fmicb.2022.950591 (PMC9481467; doi:10.3389/fmicb.2022.950591)

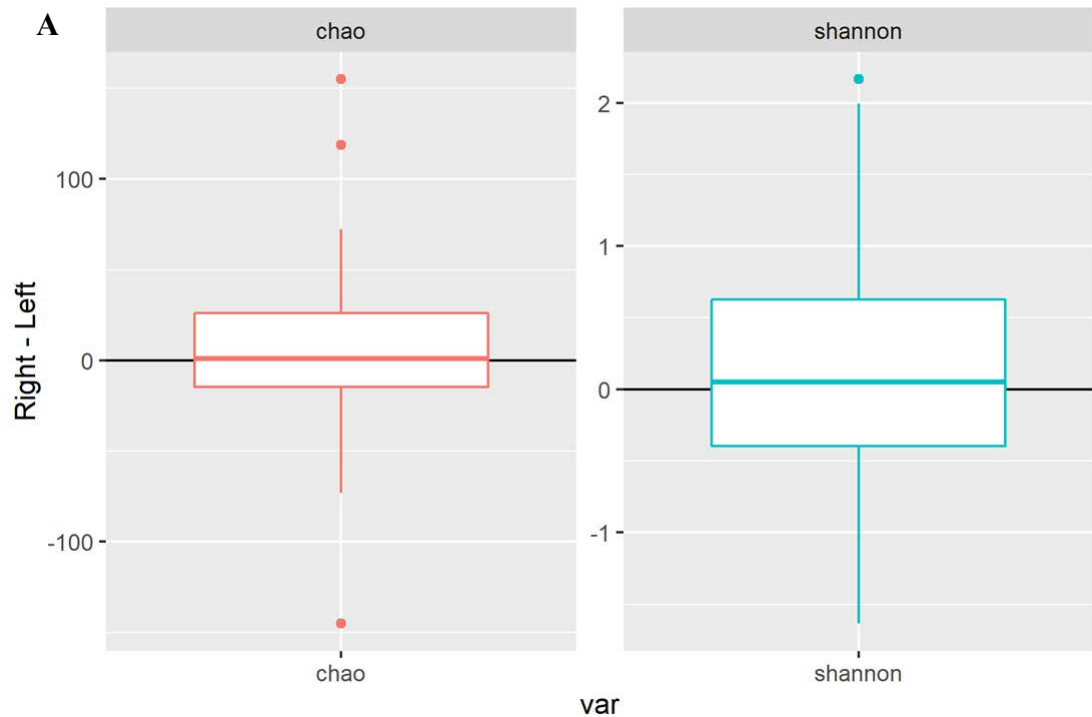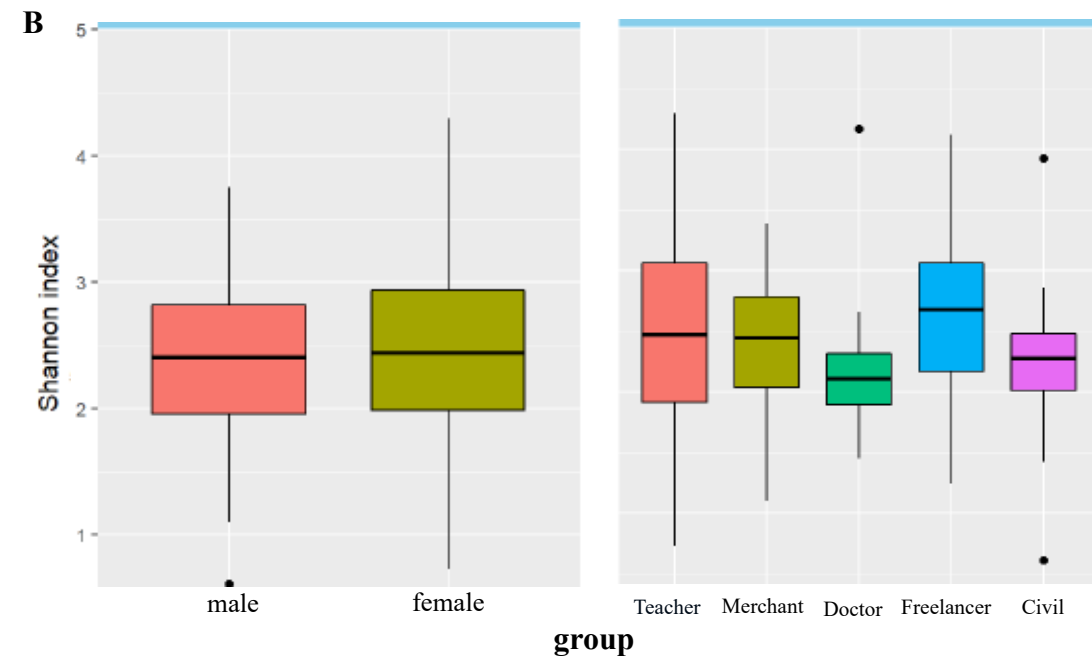

**Supplementary Figure 1. Group differences in alpha diversity at the species level**

Supplement: Supplementary Figure 1 — Group differences in alpha diversity at the species level. [file Image_1.PDF]

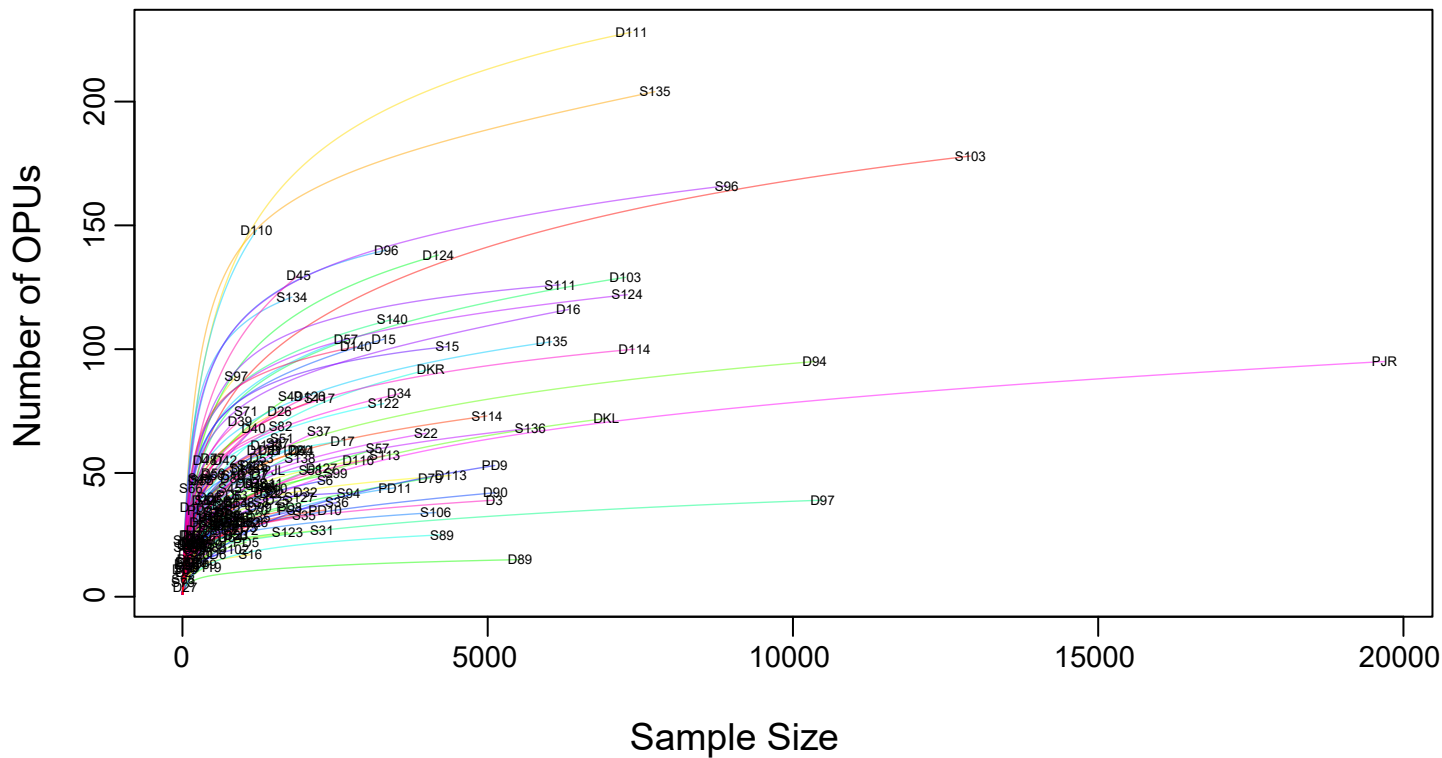

Supplement: Supplementary Figure 2 — The rarefaction curves. [file Image_2.PDF]

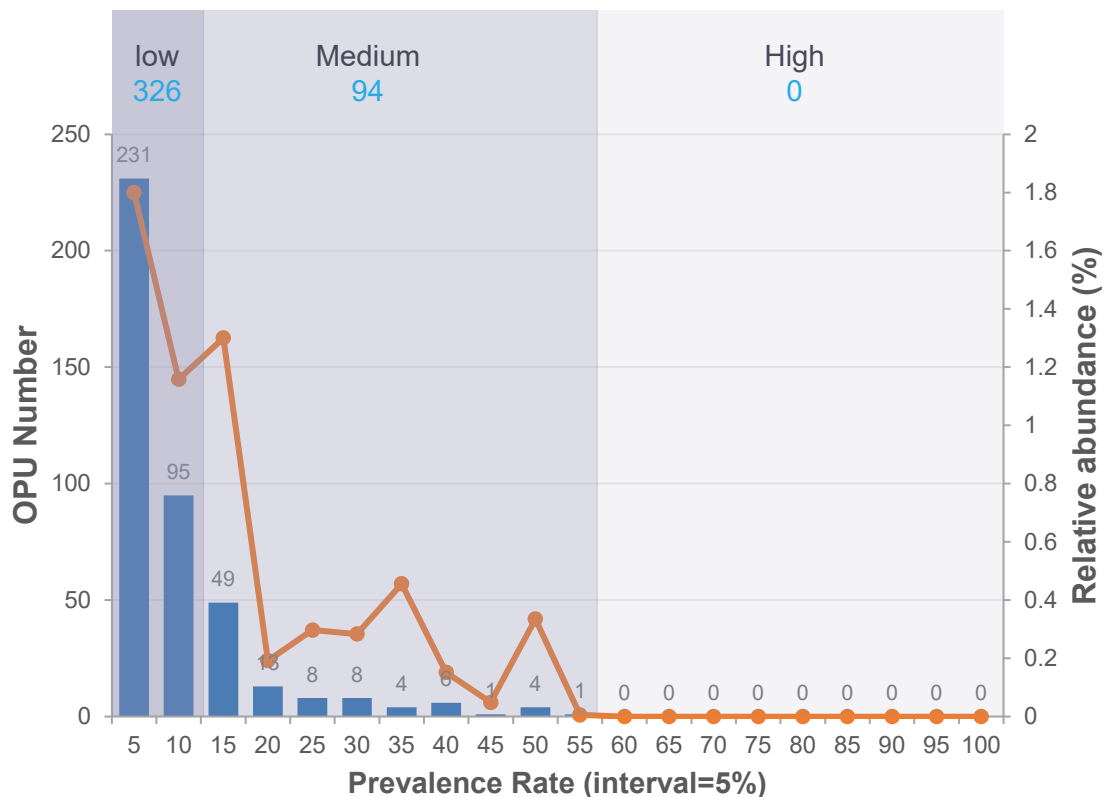

**Supplementary Figure 3. The distribution and detection rate of cultured strains were isolated**

Supplement: Supplementary Figure 3 — The distribution and detection rate of cultured strains were isolated. [file Image_3.PDF]

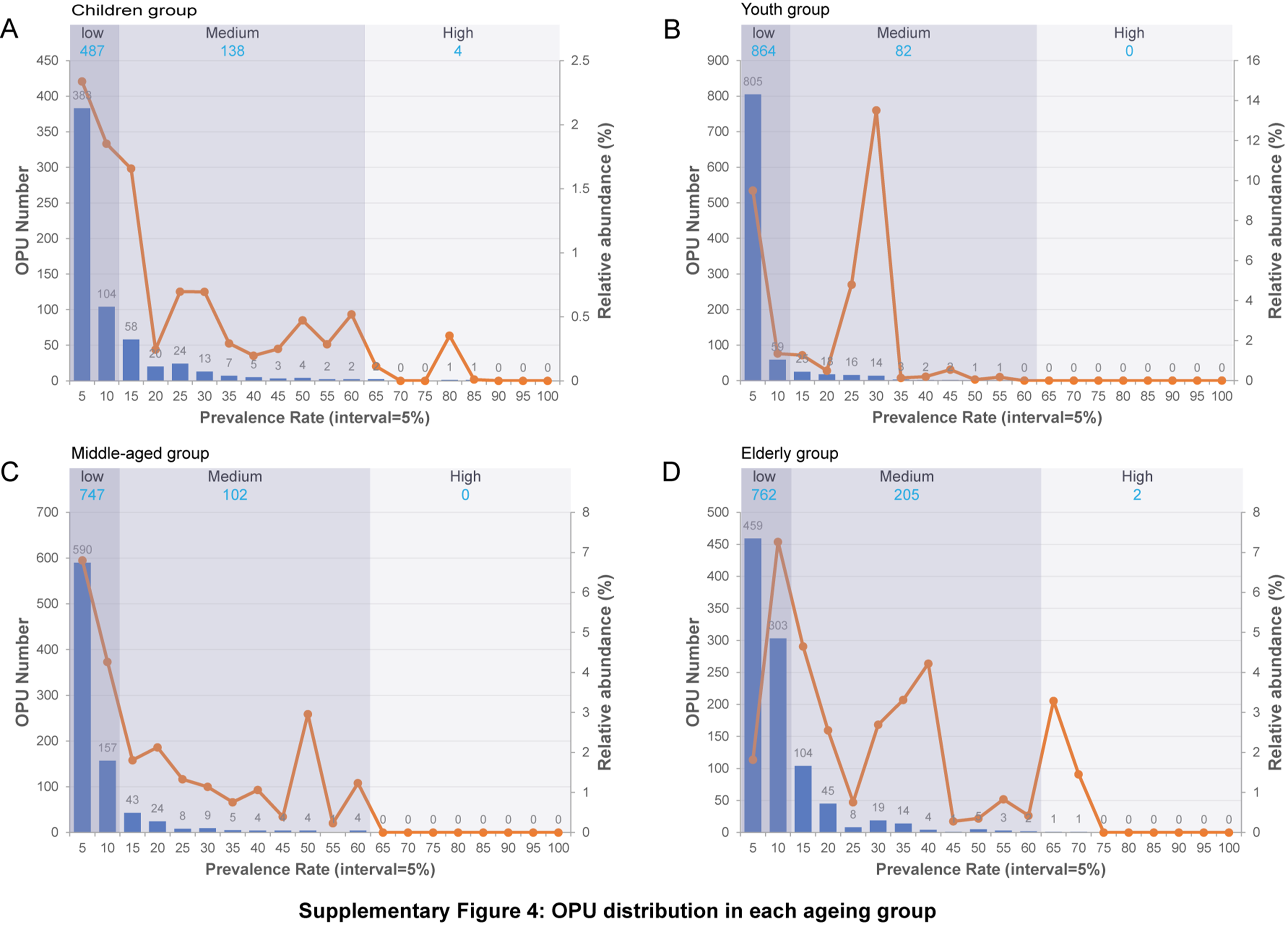

Supplement: Supplementary Figure 4 — OPU distribution in each aging group. [file Image_4.tif]

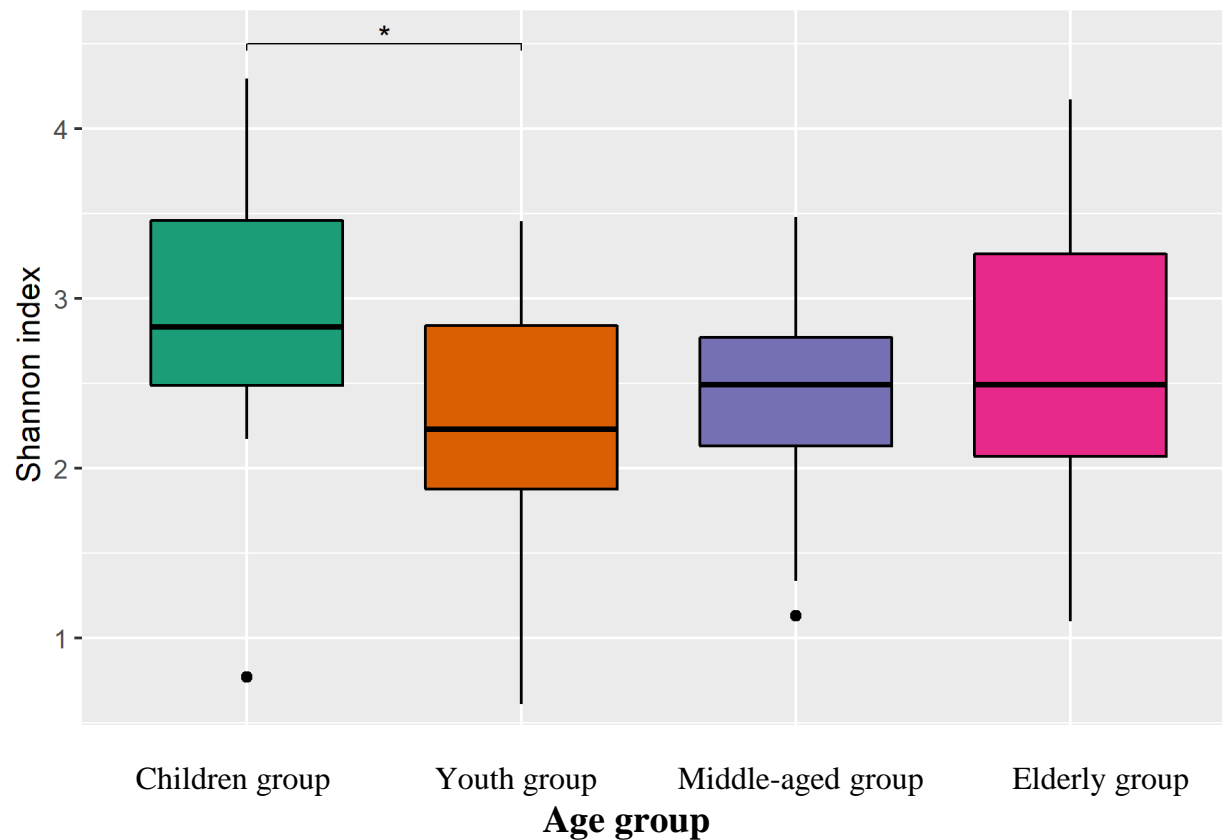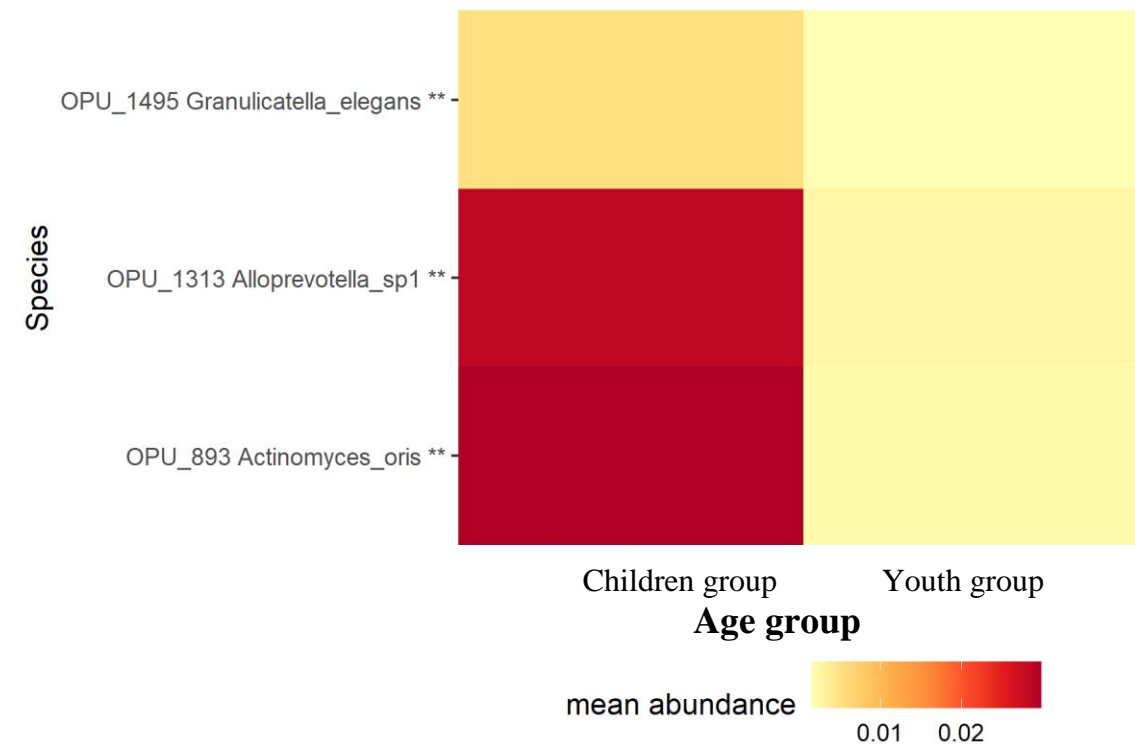

**Supplementary Figure 5: Diversity of species abundance in different age groups**

Supplement: Supplementary Figure 5 — Diversity of species abundance in different age groups. [file Image_5.PDF]
