# Supplementary material for: The species-level microbiota of healthy eyes revealed by the integration of metataxonomics with culturomics and genome analysis
Source: Front Microbiol. 2022 Sep 2;13:950591. doi: 10.3389/fmicb.2022.950591 (PMC9481467; doi:10.3389/fmicb.2022.950591)
Supplement: Supplementary Table 1 — Statistical table of pathogenicity analysis of eye surface sequencing bacteria. [file Table_1.PDF]

Supplementary Table 1*:* Statistical table of pathogenicity analysis of eye surface sequencing bacteria

Serial number	#Tax tag	Species	Pathogenicity analysis
**2**	OPU 1583	*Staphylococcus capitis*	Endophthalmitis[2];Bacteraemia[3];Endocarditis[1]; Meningitis[4]; Artificial joint infection[5]; Osteomyelitis[6]
**3**	OPU 864	*Cutibacterium acnes*	Breast augmentation surgery infection[7];Periprosthetic hip infection[8];Brain abscess[9];Surgical site infection[10];Tympanitis[11]; Pneumonia[12];Endocarditis[13];Entophthalmia[14,15];Granulomatous adenitis[16]
**4**	OPU 1409	*Streptococcus oralis subsp dentisani*	Endocarditis[17];Pneumonia[18];Bloodstream infections[19]; Meningitis[20,21];Peritonitis[22];Entophthalmia[23]; Keratitis[23]
**6**	OPU 728	*Corynebacterium accolens*	Pelvic osteomyelitis[24];Granulomatous mastitis[25]
**9**	OPU 469	*Brevundimonas vesicularis*	Peritonitis[26-28]; Septic arthritis[29];Endocarditis[30];Meningitis[31];Skin infection[32]; Keratitis[33];Bacteremia[34-38]
**10**	OPU 1392	*Streptococcus sanguinis*	Endocarditis[39-41];Brain abscess[42];Periodontosis[43];
**18**	OPU 893	*Actinomyces oris*	Osteomyelitis[44]; Endocarditis[45]
**21**	OPU 912	*Actinomyces odontolyticus*	Mediastinal abscess[46];Nephropyelitis[47]
**25**	OPU 696	*Corynebacterium tuberculostearicum*	Chronic nasosinusitis[48] ;The orthopaedic infections[49]; Granulomatous mastitis[50]
**26**	OPU 695	*Corynebacterium simulans*	Suppurative spondylitis[51];Psoriatic arthritis[52]
**28**	OPU 1447	*Lactobacillus iners*	Bacterial vaginitis[53]
**33**	OPU 1557	*Staphylococcus xylosus*	Knee prosthesis infection[54]
**37**	OPU 1294	*Prevotella timonensis*	Bacterial vaginitis[55]; Cervical intraepithelial neoplasm[56]
**46**	OPU 1399	*Streptococcus australis*	Periodontitis[57]
**48**	OPU 213	*Neisseria elongata*	Endocarditis[58-60]
**53**	OPU 1558	*Staphylococcus cohnii*	Endocarditis[61]; Scytitis[62]
**65**	OPU 1569	*Staphylococcus aureus*	Endocarditis[63,64]; Osteomyelitis[65]
**66**	OPU 841	*Rothia dentocariosa*	Endocarditis[66];Periodontitis[67];Keratohelcosis[68]

**67**	OPU 199	*Lautropia mirabilis*	Stomatitis[69,70]
**69**	OPU 1612	*Gemella sanguinis*	Endocarditis[71-73]; Pericarditis[74]
**72**	OPU 894	*Actinomyces naeslundii*	Rheumatoid arthritis[75]; Periodontitis[67]
**85**	OPU 1655	*Peptoniphilus harei*	Bacteremia[76];Osteomyelitis[77]
**88**	OPU 1424	*Streptococcus sinensis*	Endocarditis[78-81]
**92**	OPU 365	*Paracoccus yeei*	Peritonitis[82,83]; Endophthalmitis[84]
**102**	OPU 783	*lawsonella clevelandensis*	Rheumatoid arthritis[85]
**107**	OPU 1460	*Leuconostoc mesenteroides*	Chronic inflammatory skin disease[86]
**108**	OPU 1209	*Capnocytophaga gingivalis*	Bacteremia[87];Periodontitis[88]
**111**	OPU 1306	*Prevotella nigrescens*	Periodontal disease [89]Cystic fibrosis[90]
**115**	OPU 763	*Corynebacterium jeikeium*	Otitis media[11]Keratitis[91]
**118**	OPU 858	*Dermabacter jinjuensis*	Gangrene infection[92]
**120**	OPU 095	*Psychrobacter sanguinis*	Meningitis [93,94]
**125**	OPU 717	*Corynebacterium mucifaciens*	Endocarditis[95]Keratohelcosis[96]Pneumonia[97]Bacteremia [98]
**126**	OPU 1354	*Campylobacter showae*	Bacteremia [99]Periodontitis[100]
**127**	OPU 1739	*Catonella morbi*	Meningitis [101];The pulp infection[102]
**128**	OPU 749	*Corynebacterium glucuronolyticum*	Pneumonia[103];eproductive infection[104,105];ranulomatous mastitis[50]
**130**	OPU 1631	*Anaerococcus nagyae*	Pyohemia[106]
**141**	OPU 034	*Haemophilus paraphrohaemolyticus*	Cystic fibrosis[107]
**143**	OPU 865	*Propionibacterium namnetense*	Hepatapostema [108];one infection[109-111]
**149**	OPU 1570	*Staphylococcus lugdunensis*	Suppurative pericarditis[112];acteremia [113];rosthesis knee infection[114]
**153**	OPU 1776	*Selenomonas infelix*	Bacteremia [115];ingivitis[116]
**155**	OPU 697	*Corynebacterium pseudodiphtheriticum*	Suppurative arthritis[117];Bronchitis[118];Respiratory disease [119]
**160**	OPU 1204	*Capnocytophaga ochracea*	Spticemia [120-122] Dntal plaque [123]
**161**	OPU 1161	*Treponema denticola*	Periodontal disease [124-129]

**177**	OPU 1353	*Campylobacter concisus*	Gingivitis[130-133]
**179**	OPU 1641	*Anaerococcus lactolyticus*	Urinary tract pathogens [134]
**180**	OPU 1084	*Tsukamurella tyrosinosolvens*	Oular infection [135];Bacteremia ;umonia[136]
**184**	OPU 926	*Gardnerella vaginalis*	Bacterial vaginitis(BV)[137,138]
**186**	OPU 1783	*Selenomonas artemidis*	Bacteremia [115]
**187**	OPU 1769	*Dialister pneumosintes*	Periodontitis[139-141]
**190**	OPU 1318	*Alloprevotella rava*	Oteomyelitis [142]
**191**	OPU 1489	*Dolosigranulum pigrum*	Acute multiple sclerosis [143] ;orneal abscess[144];Hard grain sex Keratitis[145];rthritis[146]
**193**	OPU 1166	*Leptotrichia trevisanii*	Villoamnitis[147,148] ;cute genital infection[149,150] ;acteremia [151-154]
**194**	OPU 071	*Acinetobacter junii*	Cellulitis[155] ;eratohelcosis[156];acteremia [157,158] ;uman pathogen [159]
**198**	OPU 097	*Moraxella catarrhalis*	Atypical pituitary abscess [160];Upper respiratory tract infection in children [161,162]
**200**	OPU 197	*Burkholderia stabilis*	Cystic fibrosis[163,164]
**201**	OPU 1256	*Porphyromonas somerae*	Chronic skin and soft tissue or bone infections [165]
**204**	OPU 1466	*Enterococcus faecium*	Important hospital pathogens [166-169]
**206**	OPU 1418	*Streptococcus intermedius*	Hepatapostema [170-173];Endocarditis[174,175]Entophthalmia [176]Pelvospondylitis[177]
**207**	OPU 065	*Acinetobacter parvus*	Bacteremia [178]
**210**	OPU 1279	*Prevotella bivia*	Intracranial abscess[179];Endocarditis[180];Peritonitis[181];Lemierre's syndrome [182]
**211**	OPU 721	*Corynebacterium aquatimens*	Bacteremia [183]
**216**	OPU 1168	*Leptotrichia buccalis*	Bacteremia [184,185];horioamnionitis[186];eptic shock[187]
**218**	OPU 1307	*Prevotella corporis*	Odontogenic abscess[188]
**224**	OPU 751	*Corynebacterium pyruviciproducens*	Mammitis [189]
**227**	OPU 908	*Actinomyces graevenitzii*	Lung abscess [190] [191] Bacteremia [192] Pulmonary infection [193]
**229**	OPU 039	*Aggregatibacter aphrophilus*	Encephalopyosis [194,195] Spinal epidural abscess [196] Left superficial temporal abscess [197] Endocarditis[198-200] Entophthalmia [201] Abscesses of the retroperitoneum, psoas major and scrotum [202]Bronchiectasia[203] Chronic lacrimal cutis [204] Arthritis[205] Vntriculitis [206] multiple abscess[207]
**238**	OPU 935	*Brevibacterium epidermidis*	Endocarditis[208] Central venous line infection [209]

**241**	OPU 1355	*Campylobacter gracilis*	The pulp infection[210-212] Bacteremia [213]
**244**	OPU 1729	*Eubacterium rectale*	colonitis[214]
**245**	OPU 341	*Haematobacter massiliensis*	Endocarditis[215]
**246**	OPU 857	*Dermabacter hominis*	Encephalopyosis [216] Sbaceous cyst [217] nephritis [218] Bacteremia [219,220]
**254**	OPU 1767	*Dialister micraerophilus*	bartholinitis[221]
**256**	OPU 764	*Corynebacterium urealyticum*	urinary tract infection[222,223] Cystitis, pyelonephritis,Bacteremia [224]
**260**	OPU 190	*Massilia timonae*	Entophthalmia [225] Systemic lymphadenopathy [226] Corneal abscess[227]
**261**	OPU 909	*Actinomyces turicensis*	Bacteremia [228,229] Pleural pyothorax[230] Necrotizing soft tissue infection [231,232]Central nervous system (CNS) infectionMeningitis [233]
**269**	OPU 609	*Methylobacterium mesophilicum*	Central duct infection [234]Meningitis [235] arthromeningitis [236]
**270**	OPU 1673	*Eubacterium brachy*	Periodontitis[237-239] Breast abscess[240]Lung abscess [241]
**272**	OPU 1419	*Streptococcus constellatus*	pelvic infection [242] pneumothorax [243,244] mediastinitis [245] Bacteremia [246] Odontogenic infection [247] [248] Peritonitis[249] Septic shock[250] suppurative spondylitis [249] Herpes zoster of the ocular branch of the trigeminal ganglion conceals cavernous sinus thrombosis [251]
**274**	OPU 069	*Acinetobacter ursingii*	Bacteremia [252,253]
**275**	OPU 716	*Corynebacterium afermentans*	Bacteremia [254] Pyohemia[255] multiple abscess[256] Lung abscess [257]
**279**	OPU 221	*Neisseria oralis*	Bacteremia [258] acute cystitis [259]
**280**	OPU 450	*Ochrobactrum pseudogrignonense*	Bacteremia [260]
**282**	OPU 1091	*Gordonia sputi*	Bacteremia [261-263] Encephalopyosis [264] Meningitis [265] Entophthalmia [262] Peritonitis[262]
**283**	OPU 1263	*Parabacteroides distasonis*	Splenic abscess[266]
**284**	OPU 744	*Corynebacterium mastitidis*	Suppurative adenitis of the prepuce [267]
**292**	OPU 661	*Roseomonas mucosa*	Endocarditis[268] Bacteremia [269] Entophthalmia [270] Peritonitis[271]
**293**	OPU 915	*Mobiluncus curtisii*	Bacteremia [272-274] bacterial vaginosis [275-277]
**297**	OPU 601	*Aureimonas altamirensis*	Peritonitis[278,279] Bacteremia [280] phlegmon [281]
**298**	OPU 033	*Haemophilus influenzae*	Otitis media[282,283]
**301**	OPU 1280	*Prevotella amnii*	Spinal epidural abscess [284]

**307**	OPU 933	*Brevibacterium casei*	Entophthalmia [285];septicemia[286]
**308**	OPU 1132	*Atopobium vaginae*	Bacteremia [287];Peritonitis[288]
**311**	OPU 1467	*Enterococcus casseliflavus*	Infection of blood, urinary tract and surgical wound [289]， Entophthalmia [290]
**313**	OPU 1663	*Bacteroides coagulans*	Arthritis[291]
**318**	OPU 739	*Corynebacterium massiliense*	Bacteremia [292]
**319**	OPU 1800	*Jonquetella anthropi*	periodontal disease [293,294]
**323**	OPU 1770	*Negativicoccus succinicivorans*	Bacteremia [295]
**327**	OPU 1297	*Prevotella pleuritidis*	Lung abscess [296];purulent pleurisy [297]
**337**	OPU 1134	*Atopobium parvulum*	Dental implant infection [298]
**338**	OPU 1083	*Tsukamurella pulmonis*	Bacteremia [299];conjunctivitis [300]
**340**	OPU 698	*Corynebacterium propinquum*	Endocarditis[301];Pneumonia[302];Keratitis[303];Elbow pus [304];hydrothorax [305]
**347**	OPU 043	*Halomonas johnsoniae*	Bacteremia [306]
**350**	OPU 1578	*Staphylococcus hominis*	Bacteremia,ndocarditis[307]
**352**	OPU 1683	*Peptostreptococcus anaerobius*	urinary tract infection[308];Endocarditis[309];Osteomyelitis[310]
**353**	OPU 735	*Corynebacterium tuscaniense*	Bacteremia [183]
**356**	OPU 1524	*Bacillus circulans*	Entophthalmia [311];spondylitis [312];septicemia[313];Endocarditis[314];Cellulitis[315];Knuckle infection [316]
**357**	OPU 1464	*Weissella viridescens*	acne [317]
**363**	OPU 1560	*Staphylococcus sciuri*	Peritonitis[318];Cellulitis[319];urinary tract infection[320];pelvic inflammation [321];Septic shock[322];wound infection [323];pyaemia[324];Endocarditis[325]
**370**	OPU 910	*Actinomyces cardiffensis*	Bacteremia [326]
**371**	OPU 1266	*Tannerella forsythia*	Periodontitis[327]
**372**	OPU 087	*Moraxella atlantae*	Keratitis[328];Endocarditis[329];Bacteremia [330];Bone marrow infection [331];pyaemia [332]
**374**	OPU 1090	*Gordonia bronchialis*	Endocarditis[333];Entophthalmia [334];Bacteremia [335];Bone infection[336];Osteomyelitis[337];Peritonitis[338];skin nodules [339];dermapostasis [340];Bacteremia [341];Recurrent breast infection [342]
**380**	OPU 742	*Corynebacterium confusum*	The foot infections [343]

**384**	OPU 927	*Scardovia wiggsiae*	decayed tooth [344]	
**386**	OPU 1281	*Prevotella copri*	Bacteremia [345]	
**394**	OPU 1158	*Treponema amylovorum*	Periodontal disease[346]	
**407**	OPU 1627	*Clostridium ramosum*	ear infection;Bacteremia [347];Infectious thoracic aortic aneurysm [348];Suppurative arthritis[349];Encephalopyosis [350];discitis [351]	
**409**	OPU 870	*Propionimicrobium lymphophilum*	Bacteremia [352];urinary tract infection[353]	
**411**	OPU 1108	*Nocardia farcinica*	Bacteremia [354];Keratitis[355];infection of joint [356];Pneumonia[357];Orbital and intracranial infection [358];Psoas major and neck abscess [359];Suppurative pericarditis[360];Encephalopyosis [361];Meningitis [362];Mammitis [363];Entophthalmia [364];myelitis[365];Prostatic abscess [366]	
**413**	OPU 754	*Corynebacterium kroppenstedtii*	Granulomatous mastitis[367];gathered breast [368];Infection after dacryocystosis [369];Endocarditis[370]	
**415**	OPU 1325	*Bacteroides dorei*	Bacteremia [371]	
**424**	OPU 1626	*Solobacterium moorei*	Middle ear infection, wound infection after total laryngectomy, jaw abscess [372];Bacteremia [373];Thrombophlebitis of the femoral vein and septic pulmonary embolism [374];pyaemia [375]	
**443**	OPU 1081	*Mycobacterium iranicum*	Arthritis[376];Bacteremia [377];granuloma [378];Peritonitis[379]	
**451**	OPU 1076	*Mycobacterium rhodesiae*	Pneumonia[380];Peritonitis[381]	
**454**	OPU 1133	*Atopobium deltae*	Fournier's gangrene[382,383]Pyohemia[383]	
**460**	OPU 1721	*Ruminococcus torques*	irritable bowel syndrome [384,385];chronic nephrosis [386]Graves'[387];Type 2 diabetes [388,389]; colonitis[390];AMD[391];constipation [392]infantile autism [393]urinary tract infection[394];hiv-1[395];Crohn's disease [396]	
**463**	OPU 1278	*Prevotella baroniae*	The pulp infection[397];Periodontitis[398,399];Lung abscess [400]	
**465**	OPU 1400	*Streptococcus cristatus*	Arthritis[401];Endocarditis[402,403];dental plaque[404];decayed tooth [404-406]	
**475**	OPU 779	*Dietzia papillomatosis*	Immunofusion reticular papillomatosis [407]	
**478**	OPU 1677	*Peptoanaerobacter stomatis*	Periodontitis[408]	
**481**	OPU 226	*Neisseria weaveri*	Cellulitis,acteremia [409-411]	
**485**	OPU 228	*Neisseria subflava*	Entophthalmia [412];Bacteremia [413-415];Meningitis [416];Endocarditis[417];conjunctivitis;Arthritis[418]	N
**490**	OPU 1357	*Campylobacter ureolyticus*	gastroenteritis [419-427];Meningitis [428]	N

**491**	OPU 1765	*Dialister propionicifaciens*	HIV serum conversion, inflammation, immune cells related [429]	N
**494**	OPU 1079	*Mycobacterium llatzerense*	pulmonary infection [430];Abdominal abscess [431];Encephalopyosis [432]	N
**508**	OPU 1754	*Veillonella tobetsuensis*	lung cancer[433]	
**510**	OPU 709	*Corynebacterium argentoratense*	amygdalitis [434];RTI(respiratory tract infection) [435];Bacteremia [436];Otitis media[437]	
**519**	OPU 1264	*Parabacteroides merdae*	hypertension [438];polycystic ovarian syndrome [439];colonitis[440];Prognostic marker of liver failure [441];Parkinson[442]	
**551**	OPU 1323	*Bacteroides uniformis*	Clonitis[440]	
**554**	OPU 1493	*Aerococcus christensenii*	Chorioamnionitis[443];Endocarditis[444]	
**580**	OPU 101	*Cardiobacterium hominis*	Endocarditis[445-456];Suppurative arthritis[457];Heart infection [457];pericarditis [458];acute dacryocystitis [459];cerebral embolism [460]	
**592**	OPU 1127	*Cryptobacterium curtum*	Oral biological disorder [461]	
**593**	OPU 1131	*Olsenella uli*	Periodontitis[462-469]	
**612**	OPU 1008	*Dermacoccus nishinomiyaensis*	Bacteremia [470]	
**619**	OPU 1159	*Treponema socranskii*	Periodontitis[471 472]	
**620**	OPU 946	*Microbacterium paraoxydans*	Catheter-related infection, bloodstream infection [473],ritonitis[474]	
**621**	OPU 1574	*Staphylococcus simulans*	Uinary tract infection[475], skin infection [476], toxic shock syndrome [476,477], Osteoarthrosis [478]	
**627**	OPU 030	*Morganella morganii subsp morganii*	Postatitis;urinary tract infection[479,480]	
**636**	OPU 1126	*Slackia exigua*	Bacteremia [481], Joint infection around the prosthesis [482], septic shock [483]	
**639**	OPU 917	*Actinomyces europaeus*	Necrotizing infection [484], galactapostema [485,486]	
**640**	OPU 102	*Cardiobacterium valvarum*	Arthritis[487], Endocarditis[488]	
**644**	OPU 1020	*Streptomyces somaliensis*	Cranial and epidural myceloma [489]	
**646**	OPU 998	*Kytococcus schroeteri*	Endocarditis[490], Ventriculoperitoneal shunt infection [491], Pneumonia,Bacteremia [492]	
**655**	OPU 1718	*Moryella indoligenes*	Bacteremia [493]	
**656**	OPU 1321	*Bacteroides caccae*	bloodstream infection [494]	
**657**	OPU 1135	*Atopobium rimae*	Bacteremia [495]，Tooth apical abscess [496]	

**672**	OPU 1162	*Treponema putidum*	The pulp infection[497], Infectious dermatitis [498,499]
**690**	OPU 1742	*Clostridium butyricum*	Necrotizing colonitis of the small intestine [500]
**699**	OPU 015	*Tatumella ptyseos*	Spticemia[501],
**703**	OPU 1157	*Treponema maltophilum*	Periodontitis[502]
**720**	OPU 1779	*Centipeda periodontii*	Periodontitis[503]
**732**	OPU 546	*Sphingobium yanoikuyae*	Cntral nervous system infection[504]
**751**	OPU 194	*Ralstonia insidiosa*	Hospital infection [505]
**756**	OPU 835	*Kocuria kristinae*	Pneumonia,Bacteremia [506]; Keratitis[507]
**775**	OPU 920	*Actinotignum schaalii*	Uinary tract infection; Bacteremia [352]
**781**	OPU 1713	*Clostridium bolteae*	Neuromyelitis optica [508] Atism [509]
**782**	OPU 1753	*Veillonella parvula*	Bacteremia [510]; Meningitis [511]
**789**	OPU 1614	*Gemella asaccharolytica*	Recognized conditionally pathogenic bacteria on animal mucous membranes [512]
**791**	OPU 1412	*Streptococcus tigurinus*	Endocarditis;Meningitis [513]Oal infection[514]
**792**	OPU 1548	*Facklamia hominis*	Bacteremia , Uemia[515]

1. Douedi S, Odak M, Ravin A, Campbell N. Staphylococcus capitis Endocarditis of a Native Valve. *Cureus*. Jun 2021;13(6):e15738. doi:10.7759/cureus.15738
2. Elhusseiny AM, Shamim MM, Sanders RN, Sallam AB. Endogenous endophthalmitis caused by Staphylococcus capitis. *American journal of ophthalmology case reports*. Mar 2022;25:101415. doi:10.1016/j.ajoc.2022.101415
3. Marr I, Swe K, Henderson A, Lacey JA, Carter GP, Ferguson JK. Cefazolin susceptibility of coagulase-negative staphylococci (CoNS) causing late-onset neonatal bacteraemia. *The Journal of antimicrobial chemotherapy*. Feb 2 2022;77(2):338-344. doi:10.1093/jac/dkab402
4. Khan FY. Adult Coagulase-Negative Staphylococcal Meningitis in Qatar: Clinical Characteristics and Therapeutic Outcomes. *Asian journal of neurosurgery*. Oct-Dec 2021;16(4):714-718. doi:10.4103/ajns.AJNS_144_21
5. Bottagisio M, Bidossi A, Logoluso N, Pellegrini A, De Vecchi E. A spacer infection by Candida albicans secondary to a Staphylococcus capitis prosthetic joint infection: a case report. *BMC infectious diseases*. May 4 2021;21(1):416. doi:10.1186/s12879-021-06113-8
6. Brooks D, Thomas V, Snowden J. Staphylococcus capitis Osteomyelitis: Case Report. *Glob Pediatr Health*. 2019;6:2333794X19833736. doi:10.1177/2333794X19833736

7. Latorre MC, Alcalá L, Castellano M, et al. The role of biofilm production in Cutibacterium acnes strains isolated from breast implants. *Journal of plastic, reconstructive & aesthetic surgery : JPRAS*. Sep 13 2021;doi:10.1016/j.bjps.2021.08.014
8. Gussago S, Elzi L, Arigoni M, Poroli Bastone C, Molina MN. Simultaneous Propionibacterium avidum and Propionibacterium acnes Chronic Periprosthetic Hip Joint Infection: A Case Report. *Cureus*. Dec 2021;13(12):e20771. doi:10.7759/cureus.20771
9. Smith JL, Cruz-Gordillo P, Luiselli G, et al. Spontaneous Propionibacterium Acnes abscess with intraventricular rupture in an immunocompetent adult without prior neurosurgical intervention. *Clinical case reports*. Jan 2022;10(1):e05216. doi:10.1002/ccr3.5216
10. Siller S, Skrap B, Grabein B, Trabold R, Zausinger S, Tonn JC. Routine intraoperative microbiological smear testing in patients with reoperation after elective degenerative non-instrumented spine surgery-useful or negligible adjunct. *Acta neurochirurgica*. Jan 26 2022;doi:10.1007/s00701-022-05132-x
11. Burton M, Krumbeck JA, Wu G, et al. The adult microbiome of healthy and otitis patients: Definition of the core healthy and diseased ear microbiomes. *PloS one*. 2022;17(1):e0262806. doi:10.1371/journal.pone.0262806
12. Abdullah HM, Waqas Q, Abdalla A, Omar M, Berger P. Cutibacterium acnes Pneumonia in an Immunocompromised Patient: A Case Report and Review of the Literature. *South Dakota medicine : the journal of the South Dakota State Medical Association*. Nov 2021;74(11):523-526.
13. Fihman V, Faury H, Moussafeur A, et al. Blood Cultures for the Diagnosis of Infective Endocarditis: What Is the Benefit of Prolonged Incubation? *Journal of clinical medicine*. Dec 13 2021;10(24)doi:10.3390/jcm10245824
14. Al-Mulla AH, Al-Rushoud MW. Unusual Late Presentation of Capsular Bag Distension Syndrome Associated With Propionibacterium acnes Endophthalmitis. *Cureus*. Nov 2021;13(11):e19684. doi:10.7759/cureus.19684
15. Armstrong GW, Lin SR, Assidon A, Soukiasian S, Eliott D, Jurkunas U. Propionibacterium acnes endophthalmitis following transplantation of contaminated Descemet's membrane endothelial keratoplasty graft. *Am J Ophthalmol Case Rep*. Dec 2021;24:101227. doi:10.1016/j.ajoc.2021.101227
16. Letzelter M, Cesbron E, Bellal S, Chassain K, Maillard H. Cutaneous granulomas and granulomatous adenitis: Consider Cutibacterium acnes. *International journal of dermatology*. Oct 31 2021;doi:10.1111/ijd.15963
17. Wydall S, Durrant F, Scott J, Cheesman K. Streptococcus oralis endocarditis leading to central nervous system infection in pregnancy. *Anaesthesia reports*. Jul-Dec 2021;9(2):e12133. doi:10.1002/anr3.12133
18. Kalizang'oma A, Chaguza C, Gori A, et al. Streptococcus pneumoniae serotypes that frequently colonise the human nasopharynx are common recipients of penicillin-binding protein gene fragments from Streptococcus mitis. *Microbial genomics*. Sep 2021;7(9)doi:10.1099/mgen.0.000622
19. Chamat-Hedemand S, Bruun NE, Østergaard L, et al. Proposal for the use of echocardiography in bloodstream infections due to different streptococcal species. *BMC infectious diseases*. Jul 16 2021;21(1):689. doi:10.1186/s12879-021-06391-2

20. Cruz Cardoso J, Ferreira D, Assis R, et al. Streptococcus oralis Meningitis. *European journal of case reports in internal medicine*. 2021;8(5):002349. doi:10.12890/2021_002349
21. Nakamura Y, Uemura T, Kawata Y, Hirose B, Yamauchi R, Shimohama S. Streptococcus oralis Meningitis with Gingival Bleeding in a Patient: A Case Report and Review of the Literature. *Internal medicine (Tokyo, Japan)*. Mar 1 2021;60(5):789-793. doi:10.2169/internalmedicine.5628-20
22. Kotani A, Oda Y, Hirakawa Y, Nakamura M, Hamasaki Y, Nangaku M. Peritoneal Dialysis-Related Peritonitis Caused by Streptococcus oralis. *Internal medicine (Tokyo, Japan)*. Nov 1 2021;60(21):3447-3452. doi:10.2169/internalmedicine.6234-20
23. Santin K, Bispo PJM, Rocchetti TT, et al. Characterization and distribution of viridans group streptococci isolated from infectious endophthalmitis and keratitis. *Arquivos brasileiros de oftalmologia*. Nov-Dec 2020;83(6):463-472. doi:10.5935/0004-2749.20200087
24. Wong JS, Seaward LM, Ho CP, et al. Corynebacterium accolens-associated pelvic osteomyelitis. *Journal of clinical microbiology*. Feb 2010;48(2):654-5. doi:10.1128/jcm.00818-09
25. Ang LM, Brown H. Corynebacterium accolens isolated from breast abscess: possible association with granulomatous mastitis. *Journal of clinical microbiology*. May 2007;45(5):1666-8. doi:10.1128/jcm.02160-06
26. Paramasivam V, Paez A, Verma A, Landry D, Braden GL. Brevundimonas vesicularis Peritonitis in a Chronic Peritoneal Dialysis Patient. *Case reports in nephrology and dialysis*. Sep-Dec 2021;11(3):314-320. doi:10.1159/000517140
27. Christiadi D, Singer RF, Roberts DM. Successful Treatment of PD Peritonitis Due to Brevundimonas vesicularis. *Peritoneal dialysis international : journal of the International Society for Peritoneal Dialysis*. Sep-Oct 2018;38(5):379-381. doi:10.3747/pdi.2018.00014
28. Bolzon C, Nguyen BH. A rare case of peritonitis due to Brevundimonas vesicularis. *Journal of community hospital internal medicine perspectives*. 2018;8(3):161-162. doi:10.1080/20009666.2018.1478564
29. Sofer Y, Zmira S, Amir J. Brevundimonas vesicularis septic arthritis in an immunocompetent child. *European journal of pediatrics*. Jan 2007;166(1):77-8. doi:10.1007/s00431-006-0216-y
30. Yang ML, Chen YH, Chen TC, Lin WR, Lin CY, Lu PL. Case report: infective endocarditis caused by Brevundimonas vesicularis. *BMC infectious diseases*. Dec 29 2006;6:179. doi:10.1186/1471-2334-6-179
31. Mondello P, Ferrari L, Carnevale G. Nosocomial Brevundimonas vesicularis meningitis. *Le infezioni in medicina*. Dec 2006;14(4):235-7.
32. Panasiti V, Devirgiliis V, Mancini M, et al. A cutaneous infection caused by Brevundimonas vesicularis: a case report. *International journal of immunopathology and pharmacology*. Apr-Jun 2008;21(2):457-61. doi:10.1177/039463200802100226
33. Pelletier JS, Ide T, Yoo SH. Brevundimonas vesicularis keratitis after laser in situ keratomileusis. *Journal of cataract and refractive surgery*. Feb 2010;36(2):340-3.

doi:10.1016/j.jcrs.2009.07.050
34. Bhatawadekar SM, Sharma J. Brevundimonas vesicularis bacteremia: a rare case report in a female infant. *Indian journal of medical microbiology*. Oct-Dec 2011;29(4):420-2. doi:10.4103/0255-0857.90184
35. Zhang CC, Hsu HJ, Li CM. Brevundimonas vesicularis bacteremia resistant to trimethoprim-sulfamethoxazole and ceftazidime in a tertiary hospital in southern Taiwan. *Journal of microbiology, immunology, and infection = Wei mian yu gan ran za zhi*. Dec 2012;45(6):448-52. doi:10.1016/j.jmii.2012.01.010
36. Shang ST, Chiu SK, Chan MC, et al. Invasive Brevundimonas vesicularis bacteremia: two case reports and review of the literature. *Journal of microbiology, immunology, and infection = Wei mian yu gan ran za zhi*. Dec 2012;45(6):468-72. doi:10.1016/j.jmii.2011.12.021
37. Ben Haj Khalifa A, Bouzidi H, Sfar MT, Kheder M, Ayadi A. [Brevundimonas vesicularis bacteremia in an infant presenting with Pompe disease]. *Medecine et maladies infectieuses*. Aug 2012;42(8):370-1. Bactériémie à Brevundimonas vesicularis chez un nourrisson atteint de la maladie de Pompe. doi:10.1016/j.medmal.2012.06.001
38. Eskind CC, Doucet CA, Harris BD. Scombroid Poisoning with Concurrent Brevundimonas Septicemia: A Unique Case Report and Brief Literature Review. *Case reports in infectious diseases*. 2019;2019:2148654. doi:10.1155/2019/2148654
39. Solakyildirim K, Li Y, Bayer AS, et al. Proteoglycan 4 (lubricin) is a highly sialylated glycoprotein associated with cardiac valve damage in animal models of infective endocarditis. *Glycobiology*. Dec 18 2021;31(11):1582-1595. doi:10.1093/glycob/cwab095
40. Puccio T, Kunka KS, An SS, Kitten T. Contribution of a ZIP-family protein to manganese uptake and infective endocarditis virulence in Streptococcus sanguinis. *Molecular microbiology*. Feb 2022;117(2):353-374. doi:10.1111/mmi.14853
41. Caratti di Lanzacco L, Wauters A. Pacemaker lead rupture in a patient with subacute endocarditis: a case report. *European heart journal Case reports*. Feb 2022;6(2):ytac054. doi:10.1093/ehjcr/ytac054
42. Chang CY, Chai CSY, Ong ELC. Streptococcus sanguis brain abscess as an initial manifestation of pulmonary arteriovenous malformation. *Clinical case reports*. Dec 2020;8(12):2685-2688. doi:10.1002/ccr3.3272
43. Chakraborty P, Chowdhury R, Bhakta A, Mukhopahyay P, Ghosh S. Microbiology of periodontal disease in adolescents with Type 1 diabetes. *Diabetes & metabolic syndrome*. Nov-Dec 2021;15(6):102333. doi:10.1016/j.dsx.2021.102333
44. Yamamoto S, Takegawa H, Taniike N, Takenobu T. Actinomycotic Osteomyelitis of the Mandible Diagnosed Using Matrix Assisted Laser Desorption Ionization-Time of Flight Mass Spectrometry: A Case Report. *Journal of oral and maxillofacial surgery : official journal of the American Association of Oral and Maxillofacial Surgeons*. Oct 2018;76(10):2122-2130. doi:10.1016/j.joms.2018.04.020
45. Phichaphop C, Apiwattanakul N, Wanitkun S, Boonsathorn S. Bacterial Endocarditis Caused by Actinomyces oris: First Reported Case and Literature Review. *Journal of investigative medicine high impact case reports*. Jan-Dec 2020;8:2324709620910645. doi:10.1177/2324709620910645

46. Razok A, Ali M, Aker L, Ziglam H. Actinomyces odontolyticus bacteraemia associated with cervical and mediastinal abscesses in an immunocompetent patient: First reported case in Qatar. *New microbes and new infections*. Jan 2022;45:100956. doi:10.1016/j.nmni.2022.100956
47. Kitano H, Hieda K, Kitagawa H, et al. Case Report: Emphysematous Pyelonephritis With a Congenital Giant Ureterocele. *Frontiers in pediatrics*. 2021;9:775468. doi:10.3389/fped.2021.775468
48. De Boeck I, Wittouck S, Martens K, et al. Anterior Nares Diversity and Pathobionts Represent Sinus Microbiome in Chronic Rhinosinusitis. *mSphere*. Nov 27 2019;4(6)doi:10.1128/mSphere.00532-19
49. Kalt F, Schulthess B, Sidler F, et al. Corynebacterium Species Rarely Cause Orthopedic Infections. *Journal of clinical microbiology*. Dec 2018;56(12)doi:10.1128/jcm.01200-18
50. Dobinson HC, Anderson TP, Chambers ST, Doogue MP, Seaward L, Werno AM. Antimicrobial Treatment Options for Granulomatous Mastitis Caused by Corynebacterium Species. *Journal of clinical microbiology*. Sep 2015;53(9):2895-9. doi:10.1128/jcm.00760-15
51. Ogasawara M, Matsuhisa T, Kondo T, et al. Pyogenic spondylitis with acute course caused by Corynebacterium simulans. *Journal of infection and chemotherapy : official journal of the Japan Society of Chemotherapy*. Mar 2020;26(3):294-297. doi:10.1016/j.jiac.2019.10.012
52. Olejniczak-Staruch I, Ciążyńska M, Sobolewska-Sztychny D, Narbutt J, Skibińska M, Lesiak A. Alterations of the Skin and Gut Microbiome in Psoriasis and Psoriatic Arthritis. *International journal of molecular sciences*. Apr 13 2021;22(8)doi:10.3390/ijms22083998
53. Gustin AT, Thurman AR, Chandra N, et al. Recurrent bacterial vaginosis following metronidazole treatment is associated with microbiota richness at diagnosis. *American journal of obstetrics and gynecology*. Feb 2022;226(2):225.e1-225.e15. doi:10.1016/j.ajog.2021.09.018
54. Brand YE, Rufer B. Late prosthetic knee joint infection with Staphylococcus xylosus. *IDCases*. 2021;24:e01160. doi:10.1016/j.idcr.2021.e01160
55. Petrina MAB, Cosentino LA, Rabe LK, Hillier SL. Susceptibility of bacterial vaginosis (BV)-associated bacteria to secnidazole compared to metronidazole, tinidazole and clindamycin. *Anaerobe*. Oct 2017;47:115-119. doi:10.1016/j.anaerobe.2017.05.005
56. Mitra A, MacIntyre DA, Ntritsos G, et al. The vaginal microbiota associates with the regression of untreated cervical intraepithelial neoplasia 2 lesions. *Nature communications*. Apr 24 2020;11(1):1999. doi:10.1038/s41467-020-15856-y
57. Lim YK, Park SN, Shin JH, et al. Streptococcus koreensis sp. nov., Isolated from Human Subgingival Dental Plaque of Periodontitis Lesion. *Current microbiology*. Dec 2019;76(12):1531-1536. doi:10.1007/s00284-019-01778-6
58. Getman TJ, Khiatah B, Robinson H, Saeed M. Case Report: Infective Endocarditis of Mechanical Aortic Valve Due to Neisseria elongata Bacteremia. *The American journal of case reports*. Feb 8 2022;23:e933750. doi:10.12659/ajcr.933750
59. Brandão M, Gonçalves-Teixeira P, Queirós PR, Ferreira ND, Oliveira M. Prosthetic Aortic Valve Endocarditis by Neisseria Elongata after Bentall Procedure: When

Multimodality Imaging is Key to Diagnosis. *Arquivos brasileiros de cardiologia*. May 2021;116(5):1023-1026. Endocardite de Prótese Valvular Aórtica por Neisseria Elongata após Procedimento de Bentall: Quando a Imagem Multimodal é Chave para o Diagnóstico. doi:10.36660/abc.20200706
60. Banjari M, Haddad E, Bonnet I, et al. Infective endocarditis due to Neisseria elongata: A case report and literature review. *Infectious diseases now*. Oct 2021;51(7):622-626. doi:10.1016/j.idnow.2021.01.013
61. Motta JC, Forero-Carreño C, Arango Á, Sánchez M. Staphylococcus cohnii endocarditis in native valve. *New microbes and new infections*. Nov 2020;38:100825. doi:10.1016/j.nmni.2020.100825
62. Ito Y, Sasaki T, Li Y, et al. Staphylococcus cohnii is a potentially biotherapeutic skin commensal alleviating skin inflammation. *Cell reports*. Apr 27 2021;35(4):109052. doi:10.1016/j.celrep.2021.109052
63. Quintero-Martinez JA, Hindy JR, Mahmood M, Gerberi DJ, DeSimone DC, Baddour LM. TEA Clinical Profile of Infective Endocarditis in Patients with Recent COVID-19: A Systematic Review. *The American journal of the medical sciences*. Feb 27 2022;doi:10.1016/j.amjms.2022.02.005
64. Khan Z, Warrier V, Muhammad S, McKechnie C. Subarachnoid Haemorrhage in a Patient With Suspected Infective Endocarditis in a District General Hospital: A Case Report-Based Literature Review. *Cureus*. Jan 2022;14(1):e21602. doi:10.7759/cureus.21602
65. Noosak C, Jantorn P, Meesane J, Voravuthikunchai S, Saeloh D. Dual-functional bioactive silk sericin for osteoblast responses and osteomyelitis treatment. *PloS one*. 2022;17(3):e0264795. doi:10.1371/journal.pone.0264795
66. Elkattawy S, Alyacoub R, Younes I, Mowafy A, Noori M, Mirza M. A rare report of Rothia dentocariosa endocarditis. *Journal of community hospital internal medicine perspectives*. 2021;11(3):413-415. doi:10.1080/20009666.2021.1880539
67. Aguilar-Luis MA, Casas Apayco L, Tinco Valdez C, et al. Screening and Assessment of Antimicrobial Susceptibility of Periodontopathic Bacteria in Peruvian Patients with Periodontitis: A Pilot Study. *International journal of dentistry*. 2021;2021:2695793. doi:10.1155/2021/2695793
68. Dietze J, Mauger T. Combined Rothia dentocariosa and Streptococcus viridans Corneal Ulcer in an Immunocompromised Patient. *Case reports in ophthalmological medicine*. 2021;2021:9014667. doi:10.1155/2021/9014667
69. Wang X, Luo N, Mi Q, et al. Influence of cigarette smoking on oral microbiota in patients with recurrent aphthous stomatitis. *Journal of investigative medicine : the official publication of the American Federation for Clinical Research*. Mar 2022;70(3):805-813. doi:10.1136/jim-2021-002119
70. Overmyer KA, Rhoads TW, Merrill AE, et al. Proteomics, Lipidomics, Metabolomics, and 16S DNA Sequencing of Dental Plaque From Patients With Diabetes and Periodontal Disease. *Molecular & cellular proteomics : MCP*. 2021;20:100126. doi:10.1016/j.mcpro.2021.100126
71. Ekinci O, Ozbek E. Infective Endocarditis caused by Gemella Sanguinis: A Case with Fever of Unknown Origin and Anemia. *Journal of the College of Physicians and Surgeons--Pakistan : JCPSP*. Sep 2021;31(9):1133-1134. doi:10.29271/jcpsp.2021.09.1133

72. Emmanouilidou G, Voukelatou P, Vrettos I, et al. A Case Report of Successful Conservative Treatment for Infective Endocarditis Caused by Gemella sanguinis. *Case reports in infectious diseases*. 2019;2019:9382395. doi:10.1155/2019/9382395
73. Sideris AC, Zimmermann E, Ogami T, Avgerinos DV. A rare case of isolated mitral valve endocarditis by Gemella sanguinis: Case report and review of the literature. *International journal of surgery case reports*. 2020;69:51-54. doi:10.1016/j.ijscr.2020.03.001
74. Inoue T, Nishikawa T, Kunimasa K, et al. Infectious pericarditis caused by Gemella sanguinis induced by Endobronchial Ultrasound-guided Transbronchial Needle Aspiration (EBUS-TBNA): A case report. *Respiratory medicine case reports*. 2020;30:101057. doi:10.1016/j.rmcr.2020.101057
75. Huang F, Liu X, Cheng Y, et al. Antibody to peptidoglycan recognition protein (PGLYRP)-2 as a novel biomarker in rheumatoid arthritis. *Clinical and experimental rheumatology*. Sep-Oct 2021;39(5):988-994.
76. Wan X, Wang S, Wang M, Liu J, Zhang Y. Identification of Peptoniphilus harei From Blood Cultures in an Infected Aortic Aneurysm Patient: Case Report and Review Published Literature. *Frontiers in cellular and infection microbiology*. 2021;11:755225. doi:10.3389/fcimb.2021.755225
77. Costescu Strachinaru DI, Gallez JL, Paridaens MS, Djebara S, Soete O, Soentjens P. A case of Escherichia coli and Peptoniphilus species mixed osteomyelitis successfully identified by MALDI TOF-MS with a review of the literature. *Acta clinica Belgica*. Feb 2022;77(1):126-129. doi:10.1080/17843286.2020.1783908
78. Tomlinson JS, Khan S, Curtis S, James R. Streptococcus sinensis causing infective endocarditis in the Netherlands: our experiences from the UK. *European heart journal Case reports*. Oct 2020;4(5):1-2. doi:10.1093/ehjcr/ytaa177
79. van Ommen A, Slavenburg S, Diepersloot R, de Vries Feyens CA. Fatal outcome of first case of Streptococcus sinensis in infective endocarditis in the Netherlands: a case report. *European heart journal Case reports*. Feb 2020;4(1):1-4. doi:10.1093/ehjcr/ytz237
80. San Francisco A, Tomlinson JS, Walters S, Curtis S, James R. Lesson of the month 2: When steroids stop working - infective endocarditis, the great mimicker. *Clinical medicine (London, England)*. Jan 2019;19(1):82-84. doi:10.7861/clinmedicine.19-1-82
81. Goret J, Baudinet T, Camou F, et al. Identification of Streptococcus sinensis from a patient with endocarditis using MALDI-TOF mass spectrometry, 16S rDNA- and sodA-based phylogeny. *Journal of microbiology, immunology, and infection = Wei mian yu gan ran za zhi*. Jun 2019;52(3):507-509. doi:10.1016/j.jmii.2018.04.004
82. Mohd-Afzal S, Sabouni B, Ali H, Foggensteiner L, Mortiboy D. Case Report and Review of Paracoccus yeei - A Novel Cause of Peritoneal Dialysis Peritonitis in the United Kingdom. *Saudi journal of kidney diseases and transplantation : an official publication of the Saudi Center for Organ Transplantation, Saudi Arabia*. Jul-Aug 2021;32(4):1158-1162. doi:10.4103/1319-2442.338291
83. Fosso C, Maillart E, Beun B, Touzani F, Mahadeb B, Clevenbergh P. Opportunistic peritonitis in peritoneal dialysis: The example of Paracoccus yeei. *Clinical case reports*. May 2021;9(5):e04176. doi:10.1002/ccr3.4176
84. Bhikoo R, Chong S, McLeod K, Heyworth P, McAllister IL. Paracoccus yeei, a Novel Bacterial Cause of Endophthalmitis following Intravitreal Injection. *Ocular immunology*

and inflammation. Nov 20 2020:1-3. doi:10.1080/09273948.2020.1836233
85. Gonzales Zamora JA, Romero Alvarez M, Henry Z, Baracco GJ, Dickinson G, Lichtenberger P. Liver abscess caused by Lawsonella clevelandensis in a patient with rheumatoid arthritis: A case report and literature review. *IDCases*. 2020;20:e00734. doi:10.1016/j.idcr.2020.e00734
86. Ogawa C, Inoue R, Yonejima Y, Hisa K, Yamamoto Y, Suzuki T. Supplemental Leuconostoc mesenteroides strain NTM048 attenuates imiquimod-induced psoriasis in mice. *Journal of applied microbiology*. Dec 2021;131(6):3043-3055. doi:10.1111/jam.15161
87. Lawal FJ, Baer SL. Capnocytophaga gingivalis Bacteremia After Upper Gastrointestinal Bleeding in Immunocompromised Patient. *Journal of investigative medicine high impact case reports*. Jan-Dec 2021;9:23247096211020672. doi:10.1177/23247096211020672
88. Idate U, Bhat K, Kotrashetti V, Kugaji M, Kumbar V. Molecular identification of Capnocytophaga species from the oral cavity of patients with chronic periodontitis and healthy individuals. *Journal of oral and maxillofacial pathology : JOMFP*. May-Aug 2020;24(2):397. doi:10.4103/jomfp.JOMFP_33_20
89. Kim JH, Oh JW, Lee Y, Yun JH, Choi SH, Lee DW. Quantification of Bacteria in Mouth-Rinsing Solution for the Diagnosis of Periodontal Disease. *Journal of clinical medicine*. Feb 22 2021;10(4)doi:10.3390/jcm10040891
90. Bertelsen A, Elborn JS, Schock BC. Microbial interaction: Prevotella spp. reduce P. aeruginosa induced inflammation in cystic fibrosis bronchial epithelial cells. *Journal of cystic fibrosis : official journal of the European Cystic Fibrosis Society*. Jul 2021;20(4):682-691. doi:10.1016/j.jcf.2021.04.012
91. Sahu V, Mallika Pathak M, Das P, Ravi A. Corynebacterium jeikeium as an Unusual Cause of Keratitis: A Case Report From a Tertiary Care Hospital in Chhattisgarh, India. *Cureus*. Dec 2021;13(12):e20164. doi:10.7759/cureus.20164
92. Cho SH, Park JS, Lee WK, et al. The first case of hand infection caused by Dermabacter jinjuensis in a symmetrical peripheral gangrene patient. *Annals of medicine and surgery (2012)*. Dec 2018;36:63-66. doi:10.1016/j.amsu.2018.10.008
93. Le Guern R, Wallet F, Vega E, Courcol RJ, Loïez C. Psychrobacter sanguinis: an unusual bacterium for nosocomial meningitis. *Journal of clinical microbiology*. Sep 2014;52(9):3475-7. doi:10.1128/jcm.01197-14
94. Ortiz-Alcántara JM, Segura-Candelas JM, Garcés-Ayala F, et al. Fatal Psychrobacter sp. infection in a pediatric patient with meningitis identified by metagenomic next-generation sequencing in cerebrospinal fluid. *Archives of microbiology*. Mar 2016;198(2):129-35. doi:10.1007/s00203-015-1168-2
95. Kaya A, Tekkesin AI, Kalenderoglu K, Alper AT. An unusual etiological agent of implantable cardioverter device endocarditis: Corynebacterium mucifaciens. *Indian heart journal*. Mar-Apr 2016;68(2):190-1. doi:10.1016/j.ihj.2015.11.022
96. Sanz-Rodríguez N, Almagro-Moltó M, Vozmediano-Serrano MT, Gómez-Garcés JL. [First case of a corneal ulcer associated with Corynebacterium mucifaciens]. *Enfermedades infecciosas y microbiologia clinica*. Oct 2014;32(8):543-4. Primer aislamiento de Corynebacterium mucifaciens en una úlcera corneal. doi:10.1016/j.eimc.2013.11.012

97. Djossou F, Bézian MC, Moynet D, Le Flèche-Matéos A, Malvy D. Corynebacterium mucifaciens in an immunocompetent patient with cavitary pneumonia. *BMC infectious diseases*. Dec 17 2010;10:355. doi:10.1186/1471-2334-10-355
98. Cantarelli VV, Brodt TC, Secchi C, Inamine E, Pereira Fde S, Pilger DA. Fatal case of bacteremia caused by an atypical strain of Corynebacterium mucifaciens. *The Brazilian journal of infectious diseases : an official publication of the Brazilian Society of Infectious Diseases*. Dec 2006;10(6):416-8. doi:10.1590/s1413-86702006000600013
99. Suzuki J, Sugiyama T, Ito K, et al. Campylobacter showae bacteremia with cholangitis. *Journal of infection and chemotherapy : official journal of the Japan Society of Chemotherapy*. Oct 2013;19(5):960-3. doi:10.1007/s10156-012-0524-2
100. Lugonja B, Yeo L, Milward MR, et al. Periodontitis prevalence and serum antibody reactivity to periodontal bacteria in primary Sjögren's syndrome: a pilot study. *Journal of clinical periodontology*. Jan 2016;43(1):26-33. doi:10.1111/jcpe.12485
101. Retchless AC, Kretz CB, Rodriguez-Rivera LD, et al. Oropharyngeal microbiome of a college population following a meningococcal disease outbreak. *Scientific reports*. Jan 20 2020;10(1):632. doi:10.1038/s41598-020-57450-8
102. Diao J, Yuan C, Tong P, Ma Z, Sun X, Zheng S. Potential Roles of the Free Salivary Microbiome Dysbiosis in Periodontal Diseases. *Frontiers in cellular and infection microbiology*. 2021;11:711282. doi:10.3389/fcimb.2021.711282
103. Medkour H, Laidoudi Y, Dahmana H, et al. Severe pneumonia in a street rat (Rattus norvegicus) caused by Rodentibacter rarus strain RMC2. *Open veterinary journal*. Jan-Mar 2021;11(1):165-173. doi:10.4314/ovj.v11i1.24
104. Ruiz-Pino M, Foronda-García-Hidalgo C, Alarcón-Blanco P, Gutiérrez-Fernández J. Male genitourinary infections by Corynebacterium glucuronolyticum. A review and clinical experience. *Revista espanola de quimioterapia : publicacion oficial de la Sociedad Espanola de Quimioterapia*. Oct 2019;32(5):479-484.
105. Gherardi G, Di Bonaventura G, Pompilio A, Savini V. Corynebacterium glucuronolyticum causing genitourinary tract infection: Case report and review of the literature. *IDCases*. 2015;2(2):56-8. doi:10.1016/j.idcr.2015.03.001
106. Toprak NU, Sayin E, Akilli FM, Gundogdu A. Sepsis caused by Anaerococcus nagyae after transarterial-chemoembolization for hepatocellular carcinoma: Case report and literature review. *Anaerobe*. Dec 2021;72:102464. doi:10.1016/j.anaerobe.2021.102464
107. AbdulWahab A, Taj-Aldeen SJ, Ibrahim EB, Talaq E, Abu-Madi M, Fotedar R. Discrepancy in MALDI-TOF MS identification of uncommon Gram-negative bacteria from lower respiratory secretions in patients with cystic fibrosis. *Infection and drug resistance*. 2015;8:83-8. doi:10.2147/idr.S80341
108. Yasutomi E, Ueda Y, Asaji N, et al. Liver abscess caused by Cutibacterium namnetense after transarterial chemoembolization for hepatocellular carcinoma. *Clinical journal of gastroenterology*. Feb 2021;14(1):246-250. doi:10.1007/s12328-020-01283-5
109. Aubin GG, Kambarev S, Bémer P, Lawson PA, Corvec S. Draft Genome Sequence of Highly Rifampin-Resistant Propionibacterium namnetense NTS 31307302T Isolated from a Patient with a Bone Infection. *Genome announcements*. Aug 11 2016;4(4)doi:10.1128/genomeA.00819-16

110. Aubin GG, Bémer P, Kambarev S, et al. Propionibacterium namnetense sp. nov., isolated from a human bone infection. *International journal of systematic and evolutionary microbiology*. Sep 2016;66(9):3393-3399. doi:10.1099/ijsem.0.001204

111. Corvec S, Guillouzouic A, Aubin GG, et al. Rifampin-Resistant Cutibacterium (formerly Propionibacterium) namnetense Superinfection after Staphylococcus aureus Bone Infection Treatment. *Journal of bone and joint infection*. 2018;3(5):255-257. doi:10.7150/jbji.30029

112. Khalaf SA, Mansour A, Perveze I, Fender B, Walker DR, Dandachi D. Staphylococcus lugdunensis as Cause of Septic Pericarditis. *Missouri medicine*. Nov-Dec 2021;118(6):552-555.

113. Mani S, Chandrasekharan P. Staphylococcus lugdunensis Bacteremia with an Infected Aortic Thrombus in a Preterm Infant. *Children (Basel, Switzerland)*. Jan 2 2022;9(1)doi:10.3390/children9010046

114. Charalambous LT, Kim BI, Schwartz AM, et al. Prosthetic Knee Infection with Coagulase-Negative Staphylococcus: A Harbinger of Poor Outcomes. *The Journal of arthroplasty*. Feb 20 2022;doi:10.1016/j.arth.2022.02.050

115. Bisiaux-Salauze B, Perez C, Sebald M, Petit JC. Bacteremias caused by Selenomonas artemidis and Selenomonas infelix. *Journal of clinical microbiology*. Jan 1990;28(1):140-2. doi:10.1128/jcm.28.1.140-142.1990

116. Antezack A, Boxberger M, Ben Khedher M, La Scola B, Monnet-Corti V. Isolation and description of Selenomonas timonae sp. nov., a novel Selenomonas species detected in a gingivitis patient. *International journal of systematic and evolutionary microbiology*. Oct 2021;71(10)doi:10.1099/ijsem.0.005040

117. Erturan G, Holme H, Iyer S. Corynebacterium pseudodiphtheriticum septic arthritis secondary to intra-articular injection--a case report and literature review. *J Med Microbiol*. Jun 2012;61(Pt 6):860-863. doi:10.1099/jmm.0.037937-0

118. Craig TJ, Maguire FE, Wallace MR. Tracheobronchitis due to Corynebacterium pseudodiphtheriticum. *South Med J*. Apr 1991;84(4):504-6. doi:10.1097/00007611-199104000-00026

119. Souza MC, Santos LS, Gomes DL, et al. Aggregative adherent strains of Corynebacterium pseudodiphtheriticum enter and survive within HEp-2 epithelial cells. *Mem Inst Oswaldo Cruz*. Jun 2012;107(4):486-93. doi:10.1590/s0074-02762012000400008

120. Alhifany AA, Almangour TA, Tabb DE, Levine DH. Premature Labor and Neonatal Septicemia Caused by Capnocytophaga Ochracea. *Am J Case Rep*. Jun 16 2017;18:674-676. doi:10.12659/ajcr.903824

121. Ito S, Hagiya H, Kimura K, et al. Capnocytophaga ochracea-related Bacterium Bacteremia in a Hypertrophic Cardiomyopathy Patient without Neutropenia. *Intern Med*. 2016;55(18):2731-5. doi:10.2169/internalmedicine.55.6593

122. Kristensen B, Schønheyder HC, Peterslund NA, Rosthøj S, Clausen N, Frederiksen W. Capnocytophaga (Capnocytophaga ochracea group) bacteremia in hematological patients with profound granulocytopenia. *Scand J Infect Dis*. 1995;27(2):153-5. doi:10.3109/00365549509018997

123. Hosohama-Saito K, Kokubu E, Okamoto-Shibayama K, Kita D, Katakura A, Ishihara K. Involvement of luxS in Biofilm Formation by Capnocytophaga ochracea. *PLoS One*. 2016;11(1):e0147114. doi:10.1371/journal.pone.0147114

124. Godovikova V, Goetting-Minesky MP, Timm JC, Fenno JC. Immunotopological Analysis of the Treponema denticola Major Surface Protein (Msp). *J Bacteriol*. Jan 15 2019;201(2)doi:10.1128/jb.00528-18

125. Kikuchi Y, Kimizuka R, Kato T, Okuda K, Kokubu E, Ishihara K. Treponema denticola Induces Epithelial Barrier Dysfunction in Polarized Epithelial Cells. *Bull Tokyo Dent Coll*. Nov 30 2018;59(4):265-275. doi:10.2209/tdcpublication.2017-0052

126. Kurniyati K, Li C. Genetic Manipulations of Oral Spirochete Treponema denticola. *Methods Mol Biol*. 2021;2210:15-23. doi:10.1007/978-1-0716-0939-2_2

127. Patel DT, O'Bier NS, Schuler EJA, Marconi RT. The Treponema denticola DgcA protein (TDE0125) is a functional diguanylate cyclase. *Pathog Dis*. Mar 20 2021;79(3)doi:10.1093/femspd/ftab004

128. Tanno-Nakanishi M, Kikuchi Y, Kokubu E, Yamada S, Ishihara K. Treponema denticola transcriptional profiles in serum-restricted conditions. *FEMS Microbiol Lett*. Aug 1 2018;365(16)doi:10.1093/femsle/fny171

129. Xu X, Li X, Chen Q, Yuan J. Detection of Treponema denticola in Chronic Periodontitis by Quantitative Real-Time Polymerase Chain Reaction. *J Nanosci Nanotechnol*. Mar 1 2020;20(3):1463-1469. doi:10.1166/jnn.2020.17165

130. Akutko K, Matusiewicz K. Campylobacter concisus as the etiologic agent of gastrointestinal diseases. *Adv Clin Exp Med*. Jan-Feb 2017;26(1):149-154. doi:10.17219/acem/61428

131. Miljković-Selimović B, Babić T, Kocić B, Aleksić E, Malešević A, Tambur Z. Campylobacter concisus. *J Infect Dev Ctries*. Sep 30 2021;15(9):1216-1221. doi:10.3855/jidc.14530

132. Liu F, Ma R, Wang Y, Zhang L. The Clinical Importance of Campylobacter concisus and Other Human Hosted Campylobacter Species. *Front Cell Infect Microbiol*. 2018;8:243. doi:10.3389/fcimb.2018.00243

133. Zhang L, Lee H, Grimm MC, Riordan SM, Day AS, Lemberg DA. Campylobacter concisus and inflammatory bowel disease. *World J Gastroenterol*. Feb 7 2014;20(5):1259-67. doi:10.3748/wjg.v20.i5.1259

134. Domann E, Hong G, Imirzalioglu C, et al. Culture-independent identification of pathogenic bacteria and polymicrobial infections in the genitourinary tract of renal transplant recipients. *J Clin Microbiol*. Dec 2003;41(12):5500-10. doi:10.1128/jcm.41.12.5500-5510.2003

135. Teng JLL, Tang Y, Wong SSY, et al. MALDI-TOF MS for identification of Tsukamurella species: Tsukamurella tyrosinosolvens as the predominant species associated with ocular infections. *Emerging microbes & infections*. May 9 2018;7(1):80. doi:10.1038/s41426-018-0083-4

136. Chen CH, Lee CT, Chang TC. Tsukamurella tyrosinosolvens bacteremia with coinfection of Mycobacterium bovis pneumonia: case report and literature review. *SpringerPlus*.

2016;5(1):2033. doi:10.1186/s40064-016-3707-y
137. Liu CW, Su BC, Chen JY. Tilapia Piscidin 4 (TP4) Reprograms M1 Macrophages to M2 Phenotypes in Cell Models of Gardnerella vaginalis-Induced Vaginosis. *Frontiers in immunology*. 2021;12:773013. doi:10.3389/fimmu.2021.773013
138. Kim H, Kim Y, Kang CH. In Vivo Confirmation of the Antimicrobial Effect of Probiotic Candidates against Gardnerella vaginalis. *Microorganisms*. Aug 9 2021;9(8)doi:10.3390/microorganisms9081690
139. Ghayoumi N, Chen C, Slots J. Dialister pneumosintes, a new putative periodontal pathogen. *Journal of periodontal research*. Feb 2002;37(1):75-8. doi:10.1034/j.1600-0765.2002.05019.x
140. Siqueira JF, Jr., Rôças IN. Dialister pneumosintes can be a suspected endodontic pathogen. *Oral surgery, oral medicine, oral pathology, oral radiology, and endodontics*. Oct 2002;94(4):494-8. doi:10.1067/moe.2002.125202
141. Ferraro CT, Gornic C, Barbosa AS, Peixoto RJ, Colombo AP. Detection of Dialister pneumosintes in the subgingival biofilm of subjects with periodontal disease. *Anaerobe*. Oct-Dec 2007;13(5-6):244-8. doi:10.1016/j.anaerobe.2007.09.002
142. Ulger Toprak N, Duman N, Sacak B, et al. Alloprevotella rava isolated from a mixed infection of an elderly patient with chronic mandibular osteomyelitis mimicking oral squamous cell carcinoma. *New Microbes New Infect*. Jul 2021;42:100880. doi:10.1016/j.nmni.2021.100880
143. Aguirre M, Morrison D, Cookson BD, Gay FW, Collins MD. Phenotypic and phylogenetic characterization of some Gemella-like organisms from human infections: description of Dolosigranulum pigrum gen. nov., sp. nov. *The Journal of applied bacteriology*. Dec 1993;75(6):608-12. doi:10.1111/j.1365-2672.1993.tb01602.x
144. Roitman KL, Almuzara MN, Fernández Rodriguez MJ, et al. [Dolosigranulum pigrum in corneal abscess]. *Revista Argentina de microbiologia*. Dec 8 2021;Dolosigranulum pigrum en absceso corneal. doi:10.1016/j.ram.2021.10.002
145. Sampo M, Ghazouani O, Cadiou D, Trichet E, Hoffart L, Drancourt M. Dolosigranulum pigrum keratitis: a three-case series. *BMC ophthalmology*. Jul 10 2013;13:31. doi:10.1186/1471-2415-13-31
146. Johnsen BO, Rønning EJ, Onken A, Figved W, Jenum PA. Dolosigranulum pigrum causing biomaterial-associated arthritis. *APMIS : acta pathologica, microbiologica, et immunologica Scandinavica*. Feb 2011;119(2):85-7. doi:10.1111/j.1600-0463.2010.02697.x
147. Fontanals D, García-Miralles C, Ballester R, Cochs B, Orellana R, Capilla S. Chorioamnionitis due to Leptotrichia trevisanii. *Anaerobe*. Feb 2018;49:18-20. doi:10.1016/j.anaerobe.2017.11.001
148. Kumagai J, Takiguchi Y, Shono K, et al. Acute myelogenous leukemia with Leptotrichia trevisanii bacteremia. *Intern Med*. 2013;52(22):2573-6. doi:10.2169/internalmedicine.52.9580
149. Mora-Palma JC, Rodríguez-Oliver AJ, Navarro-Marí JM, Gutiérrez-Fernández J. Emergent genital infection by Leptotrichia trevisanii. *Infection*. Feb 2019;47(1):111-114.

doi:10.1007/s15010-018-1175-8
150. Hou H, Chen Z, Tian L, Sun Z. Leptotrichia trevisanii bacteremia in a woman with systemic lupus erythematosus receiving high-dose chemotherapy. *BMC Infect Dis*. Dec 14 2018;18(1):661. doi:10.1186/s12879-018-3495-9
151. Schrimsher JM, McGuirk JP, Hinthorn DR. Leptotrichia trevisanii sepsis after bone marrow transplantation. *Emerging infectious diseases*. Oct 2013;19(10):1690-1. doi:10.3201/eid1910.121048
152. İnal N, Hazırolan G. A case of bacteremia caused by Leptotrichia trevisanii in pediatric patient with febrile neutropenia and review of literature. *Acta microbiologica et immunologica Hungarica*. May 27 2021;doi:10.1556/030.2021.01358
153. Cooreman S, Schuermans C, Van Schaeren J, et al. Bacteraemia caused by Leptotrichia trevisanii in a neutropenic patient. *Anaerobe*. Feb 2011;17(1):1-3. doi:10.1016/j.anaerobe.2010.12.002
154. Sabater Cabrera C, Fernández Blázquez A, García Carús E. Bacteremia due to Leptotrichia trevisanii after an allogeneic bone marrow transplant. *Enfermedades infecciosas y microbiologia clinica*. Jun-Jul 2017;35(6):389-390. Bacteriemia por Leptotrichia trevisanii en una paciente sometida a trasplante alogénico de médula ósea. doi:10.1016/j.eimc.2016.09.010
155. Henao-Martínez AF, González-Fontal GR, Johnson S. A case of community-acquired Acinetobacter junii-johnsonii cellulitis. *Biomedica : revista del Instituto Nacional de Salud*. Jun 2012;32(2):179-81. doi:10.1590/s0120-41572012000300004
156. Broniek G, Langwińska-Wośko E, Szaflik J, Wróblewska M. Acinetobacter junii as an aetiological agent of corneal ulcer. *Infection*. Dec 2014;42(6):1051-3. doi:10.1007/s15010-014-0647-8
157. Tsai HY, Cheng A, Liu CY, et al. Bacteremia caused by Acinetobacter junii at a medical center in Taiwan, 2000-2010. *Eur J Clin Microbiol Infect Dis*. Oct 2012;31(10):2737-43. doi:10.1007/s10096-012-1622-x
158. Khim LP, Teng TZJ, Shelat VG. Acinetobacter junii Cholangitis with a Metallic Biliary Stent for Palliation of Klatskin Tumor. *Surgical infections*. Oct 20 2021;doi:10.1089/sur.2021.264
159. Hung YT, Lee YT, Huang LJ, et al. Clinical characteristics of patients with Acinetobacter junii infection. *Journal of microbiology, immunology, and infection = Wei mian yu gan ran za zhi*. Feb 2009;42(1):47-53.
160. Kawano T, Shinojima N, Hanatani S, Araki E, Mikami Y, Mukasa A. Atypical pituitary abscess lacking rim enhancement and diffusion restriction with an unusual organism, Moraxella catarrhalis: A case report and review of the literature. *Surgical neurology international*. 2021;12:617. doi:10.25259/sni_835_2021
161. Padra M, Benktander J, Padra JT, et al. Mucin Binding to Moraxella catarrhalis during Airway Inflammation Is Dependent on Sialic Acid. *American journal of respiratory cell and molecular biology*. Dec 2021;65(6):593-602. doi:10.1165/rcmb.2021-0064OC

162. Chan C, Ng D, Schryvers AB. The Role of the Moraxella catarrhalis CopB Protein in Facilitating Iron Acquisition From Human Transferrin and Lactoferrin. *Frontiers in microbiology*. 2021;12:714815. doi:10.3389/fmicb.2021.714815

163. Segonds C, Chabanon G. [Burkholderia cepacia: dangers of a phytopathogen organism for patients with cystic fibrosis]. *Annales de biologie clinique*. May-Jun 2001;59(3):259-69. Burkholderia cepacia: les dangers d'un micro-organisme phytopathogène pour les patients atteints de mucoviscidose.

164. Vandamme P, Mahenthiralingam E, Holmes B, et al. Identification and population structure of Burkholderia stabilis sp. nov. (formerly Burkholderia cepacia genomovar IV). *J Clin Microbiol*. Mar 2000;38(3):1042-7. doi:10.1128/jcm.38.3.1042-1047.2000

165. Summanen PH, Durmaz B, Väisänen ML, et al. Porphyromonas somerae sp. nov., a pathogen isolated from humans and distinct from porphyromonas levii. *J Clin Microbiol*. Sep 2005;43(9):4455-9. doi:10.1128/jcm.43.9.4455-4459.2005

166. Gao W, Howden BP, Stinear TP. Evolution of virulence in Enterococcus faecium, a hospital-adapted opportunistic pathogen. *Current opinion in microbiology*. Feb 2018;41:76-82. doi:10.1016/j.mib.2017.11.030

167. Klare I, Konstabel C, Mueller-Bertling S, et al. Spread of ampicillin/vancomycin-resistant Enterococcus faecium of the epidemic-virulent clonal complex-17 carrying the genes esp and hyl in German hospitals. *Eur J Clin Microbiol Infect Dis*. Dec 2005;24(12):815-25. doi:10.1007/s10096-005-0056-0

168. Duarte B, Pereira AP, Freitas AR, et al. 2CS-CHX(T) Operon Signature of Chlorhexidine Tolerance among Enterococcus faecium Isolates. *Applied and environmental microbiology*. Dec 1 2019;85(23)doi:10.1128/aem.01589-19

169. Smith DL, Johnson JA, Harris AD, Furuno JP, Perencevich EN, Morris JG, Jr. Assessing risks for a pre-emergent pathogen: virginiamycin use and the emergence of streptogramin resistance in Enterococcus faecium. *The Lancet Infectious diseases*. Apr 2003;3(4):241-9. doi:10.1016/s1473-3099(03)00581-4

170. Issa E, Salloum T, Tokajian S. From Normal Flora to Brain Abscesses: A Review of Streptococcus intermedius. *Frontiers in microbiology*. 2020;11:826. doi:10.3389/fmicb.2020.00826

171. Mishra AK, Fournier PE. The role of Streptococcus intermedius in brain abscess. *Eur J Clin Microbiol Infect Dis*. Apr 2013;32(4):477-83. doi:10.1007/s10096-012-1782-8

172. Catalya S, Komal B, Tulpule S, Raoof N, Sen S. Isolated Streptococcus intermedius pulmonary nodules. *IDCases*. 2017;8:48-49. doi:10.1016/j.idcr.2017.03.007

173. Livingston LV, Perez-Colon E. Streptococcus intermedius Bacteremia and Liver Abscess following a Routine Dental Cleaning. *Case reports in infectious diseases*. 2014;2014:954046. doi:10.1155/2014/954046

174. Salem A, Azeez S, Stirrup J, Lawrence D, Ruparelia N. Streptococcus intermedius masquerading as fungal infective endocarditis. *British journal of hospital medicine (London, England : 2005)*. Nov 2 2019;80(11):674-675. doi:10.12968/hmed.2019.80.11.674

175. Tran MP, Caldwell-McMillan M, Khalife W, Young VB. Streptococcus intermedius causing infective endocarditis and abscesses: a report of three cases and review of the literature. *BMC Infect Dis*. Nov 10 2008;8:154. doi:10.1186/1471-2334-8-154

176. Mercer GD, Politis M, Campagnoli TR, Galic IJ, Chen JC. Delayed-Onset Streptococcus intermedius Endophthalmitis Following Anti-Vascular Endothelial Growth Factor Intravitreal Injection. *Retinal cases & brief reports*. Nov 4 2020;doi:10.1097/icb.0000000000001084
177. Georgilis K, Prifti H, Vorropoulou O, Petrocheilou-Paschou V. Streptococcus intermedius spondylodiskitis. *Eur J Clin Microbiol Infect Dis*. Sep 1994;13(9):776-7. doi:10.1007/bf02276068
178. Gaillard T, Darles C, Pons S, Martinaud C, Soler C, Brisou P. Acinetobacter parvus bacteraemia community-acquired. *International journal of medical microbiology : IJMM*. Dec 2012;302(7-8):327-9. doi:10.1016/j.ijmm.2012.10.002
179. Samantaray S, Biswas R, Sasidharan GM. Intracranial abscess due to Prevotella bivia: First case report from India. *Anaerobe*. Oct 2020;65:102249. doi:10.1016/j.anaerobe.2020.102249
180. Kentos A, Motte S, Nonhoff C, et al. Prevotella bivia as an unusual cause of endocarditis. *Eur J Clin Microbiol Infect Dis*. Feb 1994;13(2):142-5. doi:10.1007/bf01982187
181. Kostov S, Slavchev S, Dzhenkov D, Strashilov S, Yordanov A. An unusual case of fulminant generalized peritonitis secondary to purulent salpingitis caused by Prevotella bivia - case report with literature review. *Germs*. Mar 2020;10(1):51-54. doi:10.18683/germs.2020.1185
182. Huits RM, van Assen S, Wildeboer-Veloo AC, Verschuuren EA, Koeter GH. Prevotella bivia necrobacillosis following infectious mononucleosis. *The Journal of infection*. Aug 2006;53(2):e59-63. doi:10.1016/j.jinf.2005.10.016
183. Aravena-Román M, Spröer C, Siering C, Inglis T, Schumann P, Yassin AF. Corynebacterium aquatimens sp. nov., a lipophilic Corynebacterium isolated from blood cultures of a patient with bacteremia. *Syst Appl Microbiol*. Sep 2012;35(6):380-4. doi:10.1016/j.syapm.2012.06.008
184. Bhally HS, Lema C, Romagnoli M, Borek A, Wakefield T, Carroll KC. Leptotrichia buccalis bacteremia in two patients with acute myelogenous leukemia. *Anaerobe*. Dec 2005;11(6):350-3. doi:10.1016/j.anaerobe.2005.04.001
185. Vemelen K, Mertens I, Thomas J, Vandeven J, Verhaegen J, Verbist L. Bacteraemia with Leptotrichia buccalis: report of a case and review of the literature. *Acta clinica Belgica*. 1996;51(4):265-70. doi:10.1080/22953337.1996.11718520
186. Smid MC, Dotters-Katz SK, Plongla R, Boggess KA. Leptotrichia Buccalis: A Novel Cause of Chorioamnionitis. *Infectious disease reports*. Apr 15 2015;7(2):5801. doi:10.4081/idr.2015.5801
187. Tierra Rodríguez AM, Raya Fernández C. Septic shock caused by Leptotrichia buccalis in a neutropenic patient secondary to chemotherapy. *Enfermedades infecciosas y microbiologia clinica (English ed)*. Jan 2020;38(1):41-42. Shock séptico por Leptotrichia buccalis en paciente neutropénico por quimioterapia. doi:10.1016/j.eimc.2019.01.008
188. Ciodaro F, Gazia F, Galletti B, Galletti F. Hyperbaric oxygen therapy in a case of cervical abscess extending to anterior mediastinum, with isolation of Prevotella corporis. *BMJ case reports*. Jul 10 2019;12(7)doi:10.1136/bcr-2019-229873
189. Fernandez LV, Fortuny AS, Rodriguez EF. [Corynebacterium pyruviciproducens and Corynebacterium amycolatum mastitis in immunocompetent no breastfeeding

women]. *Revista Argentina de microbiologia*. Jan-Mar 2021;53(1):39-42. Mastitis por Corynebacterium pyruviciproducens y Corynebacterium amycolatum en mujeres inmunocompetentes no lactantes. doi:10.1016/j.ram.2020.06.006
190. Gliga S, Devaux M, Gosset Woimant M, Mompoint D, Perronne C, Davido B. Actinomyces graevenitzii pulmonary abscess mimicking tuberculosis in a healthy young man. *Canadian respiratory journal*. November/December 2014;21(6):e75-e77. doi:10.1155/2014/841480
191. Tietz A, Aldridge KE, Figueroa JE. Disseminated coinfection with Actinomyces graevenitzii and Mycobacterium tuberculosis: case report and review of the literature. *J Clin Microbiol*. Jun 2005;43(6):3017-22. doi:10.1128/jcm.43.6.3017-3022.2005
192. Hwang SS, Park SD, Jang IH, Uh Y, Yoon KJ, Kim HY. Actinomyces graevenitzii bacteremia in a patient with alcoholic liver cirrhosis. *Anaerobe*. Apr 2011;17(2):87-9. doi:10.1016/j.anaerobe.2011.03.002
193. Caballero Vázquez A, Cruz Rueda JJ, Ceballos Gutierrez JA. Diagnosis of Actinomyces graevenitzii Lung Infection using Linear EBUS. *Archivos de bronconeumologia*. Jun 2017;53(6):351-352. Diagnóstico mediante EBUS lineal de infección pulmonar por Actinomyces graevenitzii. doi:10.1016/j.arbres.2016.10.014
194. Fernández González R, González Noya A, Fernández-Rodríguez R. Aggregatibacter aphrophilus brain abscess after tooth manipulation. *Medicina clinica*. Oct 8 2021;157(7):355-356. Absceso cerebral por Aggregatibacter aphrophilus tras manipulación dental. doi:10.1016/j.medcli.2020.07.049
195. Maraki S, Papadakis IS, Chronakis E, Panagopoulos D, Vakis A. Aggregatibacter aphrophilus brain abscess secondary to primary tooth extraction: Case report and literature review. *Journal of microbiology, immunology, and infection = Wei mian yu gan ran za zhi*. Feb 2016;49(1):119-22. doi:10.1016/j.jmii.2013.12.007
196. Altdorfer A, Gavage P, Moerman F. Aggregatibacter aphrophilus spinal epidural abscess. *BMJ case reports*. Jul 16 2020;13(7)doi:10.1136/bcr-2020-235320
197. Moreno T, Varman R, Idicula W. Unique presentation of Aggregatibacter aphrophilus in a superficial left temporal abscess. *IDCases*. 2020;20:e00753. doi:10.1016/j.idcr.2020.e00753
198. Hirano K, Tokui T, Inagaki M, Fujii T, Maze Y, Toyoshima H. Aggregatibacter aphrophilus infective endocarditis confirmed by broad-range PCR diagnosis: A case report. *International journal of surgery case reports*. 2017;31:150-153. doi:10.1016/j.ijscr.2017.01.041
199. Lindholm M, Min Aung K, Nyunt Wai S, Oscarsson J. Role of OmpA1 and OmpA2 in Aggregatibacter actinomycetemcomitans and Aggregatibacter aphrophilus serum resistance. *Journal of oral microbiology*. 2019;11(1):1536192. doi:10.1080/20002297.2018.1536192
200. Patel SR, Patel NH, Borah A, Saltzman H. Aggregatibacter aphrophilus pacemaker endocarditis: a case report. *BMC research notes*. Dec 8 2014;7:885. doi:10.1186/1756-0500-7-885
201. Shum JW, Tsang FC, Fung KS, Li KK. Presumed Aggregatibacter aphrophilus endogenous endophthalmitis. *International ophthalmology*. Apr 2015;35(2):269-73. doi:10.1007/s10792-015-0044-z
202. Khurana A, Kowlgi NG, Naut ER. Retroperitoneal, Psoas, and Scrotal Abscesses Due to an Uncommon Organism - Aggregatibacter aphrophilus: Case Report and Review

of Literature. *Connecticut medicine*. Oct 2016;80(9):543-547.
203. Patas K, Douros K, Priftis KN, Ioannidis A, Nikolaou C, Chatzipanagiotou S. Isolation of Aggregatibacter aphrophilus from bronchoalveolar lavage in a paediatric patient presenting with haemoptysis. *New Microbes New Infect*. May 2019;29:100509. doi:10.1016/j.nmni.2019.01.005
204. Boulze-Pankert M, Roux C, Nkamga VD, et al. Aggregatibacter aphrophilus chronic lacrimal canaliculitis: a case report. *BMC ophthalmology*. Aug 2 2016;16:132. doi:10.1186/s12886-016-0312-3
205. Fernando SA, Gottlieb T. Aggregatibacter aphrophilus Sacroiliitis Following Gastroscopy in a Young Sportsman. *Clinical journal of sport medicine : official journal of the Canadian Academy of Sport Medicine*. Jan 2017;27(1):e3-e5. doi:10.1097/jsm.0000000000000311
206. Tok S, Neidert MC, Bloemberg G, Sürücü O. Aggregatibacter aphrophilus ventriculitis following C1-C2 transarticular screw fixation. *Neurologia i neurochirurgia polska*. 2016;50(1):63-8. doi:10.1016/j.pjnns.2015.11.005
207. Belkacem A, Caseris M, Yazdanpanah Y. A Case of Aggregatibacter aphrophilus Multiple Abscess. *Open forum infectious diseases*. Apr 2015;2(2):ofv031. doi:10.1093/ofid/ofv031
208. Manetos CM, Pavlidis AN, Kallistratos MS, et al. Native aortic valve endocarditis caused by Brevibacterium epidermidis in an immunocompetent patient. *The American journal of the medical sciences*. Sep 2011;342(3):257-8. doi:10.1097/MAJ.0b013e31821ffb9f
209. McCaughey C, Damani NN. Central venous line infection caused by Brevibacterium epidermidis. *The Journal of infection*. Sep 1991;23(2):211-2. doi:10.1016/0163-4453(91)92451-a
210. Siqueira JF, Jr., Rôças IN. Campylobacter gracilis and Campylobacter rectus in primary endodontic infections. *International endodontic journal*. Mar 2003;36(3):174-80. doi:10.1046/j.1365-2591.2003.00636.x
211. Siqueira JF, Jr., Rôças I. A 16S rDNA-based nested PCR protocol to detect Campylobacter gracilis in oral infections. *Pesquisa odontologica brasileira = Brazilian oral research*. Apr-Jun 2003;17(2):142-6. doi:10.1590/s1517-74912003000200008
212. Miller WG, Yee E. Complete Genome Sequence of Campylobacter gracilis ATCC 33236T. *Genome Announc*. Sep 17 2015;3(5)doi:10.1128/genomeA.01087-15
213. Shinha T. Fatal bacteremia caused by Campylobacter gracilis, United States. *Emerging infectious diseases*. Jun 2015;21(6):1084-5. doi:10.3201/eid2106.142043
214. Wang Y, Wan X, Wu X, Zhang C, Liu J, Hou S. Eubacterium rectale contributes to colorectal cancer initiation via promoting colitis. *Gut pathogens*. Jan 12 2021;13(1):2. doi:10.1186/s13099-020-00396-z
215. Cheng JW, Wang P, Xiao M, et al. First case report of endocarditis caused by haematobacter massiliensis in China. *BMC Infect Dis*. Oct 31 2017;17(1):709. doi:10.1186/s12879-017-2809-7
216. Bavbek M, Caner H, Arslan H, Demirhan B, Tunçbilek S, Altinörs N. Cerebral Dermabacter hominis abscess. *Infection*. May-Jun 1998;26(3):181-3. doi:10.1007/bf02771848

217. Bertona E, De Paulis AN, Gutiérrez MA, Santa María V, Vay CA, Predari SC. [Unusually infected sebaceous cyst by Dermabacter hominis]. *Revista Argentina de microbiologia*. Oct-Dec 2016;48(4):303-307. Un caso inusual de quiste sebáceo infectado por Dermabacter hominis. doi:10.1016/j.ram.2016.09.003
218. Albersmeier A, Bomholt C, Glaub A, et al. Draft Genome Sequence of the Multidrug-Resistant Clinical Isolate Dermabacter hominis 1368. *Genome Announc*. Jul 24 2014;2(4)doi:10.1128/genomeA.00728-14
219. Gómez-Garcés JL, Oteo J, García G, Aracil B, Alós JI, Funke G. Bacteremia by Dermabacter hominis, a rare pathogen. *J Clin Microbiol*. Jun 2001;39(6):2356-7. doi:10.1128/jcm.39.6.2356-2357.2001
220. Jones D, Collins MD. Taxonomic studies on some human cutaneous coryneform bacteria: Description of Dermabacter hominis gen.nov., sp.nov. *FEMS Microbiology Letters*. 1988;51(1):51-55. doi:10.1111/j.1574-6968.1988.tb02967.x
221. Cobo F, Rodríguez-Granger J, Sampedro A, Navarro-Marí JM. Bartholin's abscess due to Dialister micraerophilus in a woman presenting with repetitive bartholinitis episodes. *Medecine et maladies infectieuses*. May 2018;48(3):225-226. doi:10.1016/j.medmal.2017.12.008
222. Costales J, Alsyouf M, Napolitan P, Wang S, Hu B. Corynebacterium urealyticum: rare urinary tract infection with serious complications. *The Canadian journal of urology*. Feb 2019;26(1):9680-9682.
223. Rocha DJP, Azevedo V, Brenig B, et al. Whole-genome sequencing reveals misidentification of a multidrug-resistant urine clinical isolate as Corynebacterium urealyticum. *Journal of global antimicrobial resistance*. Dec 2020;23:16-19. doi:10.1016/j.jgar.2020.07.020
224. Salem N, Salem L, Saber S, Ismail G, Bluth MH. Corynebacterium urealyticum: a comprehensive review of an understated organism. *Infection and drug resistance*. 2015;8:129-45. doi:10.2147/idr.S74795
225. Chiquet C, Boisset S, Pechinot A, Creuzot-Garcher C, Aptel F, Bron AM. Massilia timonae as cause of chronic endophthalmitis following cataract surgery. *Journal of cataract and refractive surgery*. Aug 2015;41(8):1778-80. doi:10.1016/j.jcrs.2015.07.016
226. Van Craenenbroeck AH, Camps K, Zachée P, Wu KL. Massilia timonae infection presenting as generalized lymphadenopathy in a man returning to Belgium from Nigeria. *J Clin Microbiol*. Jul 2011;49(7):2763-5. doi:10.1128/jcm.00160-11
227. Ballestero-Téllez M, Laborda Guirao T, Batista Diaz N, Pascual Hernandez Á. First reported case of a corneal abscess caused by Massilia timonae. *Enfermedades infecciosas y microbiologia clinica*. Mar 2016;34(3):212-3. doi:10.1016/j.eimc.2015.07.003
228. Kansara T, Majmundar M, Doshi R, Ghosh K, Saeed M. A Case of Life-threatening Actinomyces turicensis Bacteremia. *Cureus*. Jan 24 2020;12(1):e6761. doi:10.7759/cureus.6761
229. Hagiya H, Ogawa H, Takahashi Y, Kimura K, Hasegawa K, Otsuka F. Actinomyces turicensis Bacteremia Secondary to Pyometra. *Intern Med*. 2015;54(21):2775-7. doi:10.2169/internalmedicine.54.4637

230. Agrafiotis AC, Lardinois I. Pleural empyema caused by Actinomyces turicensis. *New Microbes New Infect*. May 2021;41:100892. doi:10.1016/j.nmni.2021.100892
231. Panwar K, Duane TM, Tessier JM, Patel K, Sanders JM. Actinomyces turicensis Necrotizing Soft-Tissue Infection of the Thigh in a Diabetic Male. *Surgical infections*. Jul 2019;20(5):431-433. doi:10.1089/sur.2018.149
232. Gatti M, Gasparini LE, Grimaldi CM, et al. Septic shock due to NSTI caused by Actinomyces Turicensis: the role of clinical pharmacology. Case report and review of the literature. *Journal of chemotherapy (Florence, Italy)*. Dec 2017;29(6):372-375. doi:10.1080/1120009x.2017.1306154
233. Kocsis B, Tiszlavicz Z, Jakab G, et al. Case report of Actinomyces turicensis meningitis as a complication of purulent mastoiditis. *BMC Infect Dis*. Dec 20 2018;18(1):686. doi:10.1186/s12879-018-3610-y
234. Sanders JW, Martin JW, Hooke M, Hooke J. Methylobacterium mesophilicum infection: case report and literature review of an unusual opportunistic pathogen. *Clin Infect Dis*. Jun 2000;30(6):936-8. doi:10.1086/313815
235. Zinchuk A, Zubach O, Zadorozhnyj A, Chudina Y, Bilavka V. Characteristics of Meningitis Due to Methylobacterium mesophilicum: A Rare Case. *Japanese journal of infectious diseases*. 2015;68(4):343-6. doi:10.7883/yoken.JJID.2014.262
236. Liu JW, Wu JJ, Chen HM, Huang AH, Ko WC, Chuang YC. Methylobacterium mesophilicum synovitis in an alcoholic. *Clin Infect Dis*. May 1997;24(5):1008-9. doi:10.1093/clinids/24.5.1008
237. Hill GB, Ayers OM, Kohan AP. Characteristics and sites of infection of Eubacterium nodatum, Eubacterium timidum, Eubacterium brachy, and other asaccharolytic eubacteria. *J Clin Microbiol*. Aug 1987;25(8):1540-5. doi:10.1128/jcm.25.8.1540-1545.1987
238. Vincent JW, Falkler WA, Jr., Heath JR, 3rd. Eubacterium brachy. Reactivity in in vitro bone resorptive bioassay. *Journal of periodontology*. Feb 1984;55(2):93-7. doi:10.1902/jop.1984.55.2.93
239. Vincent JW, Falkler WA, Jr., Dalessandro NF, Miller RA, Heath JR, 3rd. Reaction of human sera with Eubacterium brachy: isolation and characterization of an extracellular antigen. *Infection and immunity*. Mar 1985;47(3):592-7. doi:10.1128/iai.47.3.592-597.1985
240. Rochford JC. Pleuropulmonary infection associated with Eubacterium brachy, a new species of Eubacterium. *J Clin Microbiol*. Nov 1980;12(5):722-3. doi:10.1128/jcm.12.5.722-723.1980
241. Yamakawa H, Hagiwara E, Hayashi M, et al. A case of relapsed lung abscess caused by Eubacterium brachy infection following an initial diagnosis of pulmonary actinomycosis. *Respiratory medicine case reports*. 2017;22:171-174. doi:10.1016/j.rmcr.2017.08.013
242. Mora-Palma JC, Guillot-Suay V, Sánchez Gila MM, Gutiérrez-Fernández J. [Pelvic inflammatory disease by Streptococcus constellatus. Clinical experience and a review]. *Rev Esp Quimioter*. Aug 2020;33(4):285-288. Enfermedad pélvica inflamatoria por Streptococcus constellatus. Experiencia clínica y revisión de la literatura. doi:10.37201/req/020.2020

243. Zhang Z, Xiao B, Liang Z. Successful treatment of pyopneumothorax secondary to Streptococcus constellatus infection with linezolid: a case report and review of the literature. *Journal of medical case reports*. Oct 7 2020;14(1):180. doi:10.1186/s13256-020-02475-w

244. Lee YJ, Lee J, Kwon BS, Kim Y. An empyema caused by Streptococcus constellatus in an older immunocompetent patient: Case report. *Medicine*. Nov 12 2021;100(45):e27893. doi:10.1097/md.0000000000027893

245. Ye RH, Yang JC, Hong HH, et al. Descending necrotizing mediastinitis caused by Streptococcus constellatus in an immunocompetent patient: case report and review of the literature. *BMC pulmonary medicine*. Feb 17 2020;20(1):43. doi:10.1186/s12890-020-1068-3

246. Chrastek D, Hickman S, Sitaranjan D, et al. Streptococcus constellatus Causing Empyema and Sepsis, Necessitating Early Surgical Decortication. *Case reports in infectious diseases*. 2020;2020:4630809. doi:10.1155/2020/4630809

247. Al Asaadi Z, Srinivasan B, Melchers LJ, Brennan PA. Streptococcus constellatus causing bony destruction secondary to odontogenic infection: three rare cases. *The British journal of oral & maxillofacial surgery*. Jul 2019;57(6):594-596. doi:10.1016/j.bjoms.2019.05.004

248. Haidar A, Haddad A, Naqvi A, Onyesoh NU, Malik R, Williams M. Streptococcus constellatus Causing Septic Thrombophlebitis of the Right Ovarian Vein with Extension into the Inferior Vena Cava. *Case reports in infectious diseases*. 2015;2015:495898. doi:10.1155/2015/495898

249. Lim SW, Lim HY, Kannaiah T, Zuki Z. Streptococcus Constellatus Spondylodiscitis in a Teenager: A Case Report. *Malaysian orthopaedic journal*. Nov 2017;11(3):50-52. doi:10.5704/moj.1711.004

250. Tymon-Rosario J, Atrio JM, Yoon HA, Erlichman D, Lerner V. Streptococcus constellatus Peritonitis and Subsequent Septic Shock following Intrauterine Device Removal. *Case reports in obstetrics and gynecology*. 2019;2019:6491617. doi:10.1155/2019/6491617

251. Lee JH, Heo HJ, Kim KM, Lee HG, Baek SM, Jung DW. Herpes zoster in the ophthalmic branch of the trigeminal ganglia obscuring cavernous sinus thrombosis due to Streptococcus constellatus subsp. constellatus - A case report. *Anesthesia and pain medicine*. Apr 30 2020;15(2):205-208. doi:10.17085/apm.2020.15.2.205

252. Daniel AM, Garzón D, Vivas A, Viviana TM, Cubides-Diaz DA, Fabian YM. Catheter-related bloodstream infection due to Acinetobacter ursingii in a hemodialysis patient: case report and literature review. *The Pan African medical journal*. 2021;39:208. doi:10.11604/pamj.2021.39.208.30565

253. Horii T, Tamai K, Mitsui M, Notake S, Yanagisawa H. Blood stream infections caused by Acinetobacter ursingii in an obstetrics ward. *Infection, genetics and evolution : journal of molecular epidemiology and evolutionary genetics in infectious diseases*. Jan 2011;11(1):52-6. doi:10.1016/j.meegid.2010.10.011

254. Bernier AM, Bernard K. Draft Genome Sequence for the Type Strain of Corynebacterium afermentans LCDC 88-0199T, Isolated from a Human Blood Culture. *Genome Announc*. Jul 7 2016;4(4)doi:10.1128/genomeA.00661-16

255. Kumari P, Tyagi A, Marks P, Kerr KG. Corynebacterium afermentans spp. afermentans sepsis in a neurosurgical patient. *The Journal of infection*. Sep 1997;35(2):201-2. doi:10.1016/s0163-4453(97)92106-x

256. Dykhuizen RS, Douglas G, Weir J, Gould IM. Corynebacterium afermentans subsp. lipophilum: multiple abscess formation in brain and liver. *Scand J Infect Dis*. 1995;27(6):637-9. doi:10.3109/00365549509047082
257. Minkin R, Shapiro JM. Corynebacterium afermentans lung abscess and empyema in a patient with human immunodeficiency virus infection. *South Med J*. Apr 2004;97(4):395-7. doi:10.1097/01.Smj.0000100114.54554.C1
258. Wolfgang WJ, Passaretti TV, Jose R, et al. Neisseria oralis sp. nov., isolated from healthy gingival plaque and clinical samples. *Int J Syst Evol Microbiol*. Apr 2013;63(Pt 4):1323-1328. doi:10.1099/ijs.0.041731-0
259. Alamri Y, Keene A, Pithie A. Acute Cystitis Caused by Commensal Neisseria oralis: A Case Report and Review of the Literature. *Infectious disorders drug targets*. 2017;17(1):64-66. doi:10.2174/1871526517666161123110613
260. Cho HW, Byun JH, Kim D, Lee H, Yong D, Lee KW. The First Case of Ochrobactrum pseudogrignonense Bacteremia in Korea. *Annals of laboratory medicine*. Jul 2020;40(4):331-333. doi:10.3343/alm.2020.40.4.331
261. Renvoise A, Harle JR, Raoult D, Roux V. Gordonia sputi bacteremia. *Emerging infectious diseases*. Sep 2009;15(9):1535-7. doi:10.3201/eid1509.080903
262. Fang W, Li J, Cui HS, et al. First identification of Gordonia sputi in a post-traumatic endophthalmitis patient - a case report and literatures review. *BMC ophthalmology*. Oct 11 2017;17(1):190. doi:10.1186/s12886-017-0573-5
263. Negishi T, Matsumoto T, Saito S, et al. Catheter-Related Bacteremia Due to Gordonia sputi in a Patient with Acute Lymphocytic Leukemia: a Case Report. *Japanese journal of infectious diseases*. Jul 22 2016;69(4):342-3. doi:10.7883/yoken.JJID.2015.487
264. Eribi A, Al-Amri K, Al-Jabri A, Osman A, Mohamed Elfadil O. Gordonia sputi related multiple brain abscesses, an AIDS-presenting illness: Thinking outside the box. *IDCases*. 2020;21:e00906. doi:10.1016/j.idcr.2020.e00906
265. Martín D, Barrios A, Domingo D, et al. Cerebrospinal fluid shunt-associated meningitis caused by Gordonia sputi: case report and review of the literature. *Infez Med*. Jun 1 2017;25(2):174-178.
266. Gunalan A, Biswas R, Sridharan B, Elamurugan TP. Pathogenic potential of Parabacteroides distasonis revealed in a splenic abscess case: a truth unfolded. *BMJ case reports*. Dec 13 2020;13(12)doi:10.1136/bcr-2020-236701
267. Radaelli E, Manarolla G, Pisoni G, et al. Suppurative adenitis of preputial glands associated with Corynebacterium mastitidis infection in mice. *Journal of the American Association for Laboratory Animal Science : JAALAS*. Jan 2010;49(1):69-74.
268. Shao S, Guo X, Guo P, Cui Y, Chen Y. Roseomonas mucosa infective endocarditis in patient with systemic lupus erythematosus: case report and review of literature. *BMC Infect Dis*. Feb 12 2019;19(1):140. doi:10.1186/s12879-019-3774-0
269. Kimura K, Hagiya H, Nishi I, Yoshida H, Tomono K. Roseomonas mucosa bacteremia in a neutropenic child: A case report and literature review. *IDCases*. 2018;14:e00469.

doi:10.1016/j.idcr.2018.e00469
270. Bhende M, Karpe A, Arunachalam S, Therese KL, Biswas J. Endogenous endophthalmitis due to Roseomonas mucosa presenting as a subretinal abscess. *Journal of ophthalmic inflammation and infection*. Dec 2017;7(1):5. doi:10.1186/s12348-017-0123-6
271. Beucler N, Meyer M, Choucha A, Seng P, Dufour H. Peritonitis caused by Roseomonas mucosa after ventriculoperitoneal shunt revision: a case report. *Acta neurochirurgica*. Oct 2020;162(10):2459-2462. doi:10.1007/s00701-020-04449-9
272. Gomez-Garces JL, Balas D, Merino MT, Ignacio Alos J. Mobiluncus curtisii bacteremia following septic abortion. *Clin Infect Dis*. Dec 1994;19(6):1166-7. doi:10.1093/clinids/19.6.1166
273. Sahuquillo-Arce JM, Ramirez-Galleymore P, Garcia J, Marti V, Arizo D. Mobiluncus curtisii bacteremia. *Anaerobe*. Apr 2008;14(2):123-4. doi:10.1016/j.anaerobe.2007.12.003
274. Hill DA, Seaton RA, Cameron FM, McLellan A, Brown R, France AJ. Severe sepsis caused by Mobiluncus curtisii subsp. curtisii in a previously healthy female: case report and review. *The Journal of infection*. Sep 1998;37(2):194-6. doi:10.1016/s0163-4453(98)80180-1
275. Zeng W, Ma H, Fan W, et al. Structure determination of CAMP factor of Mobiluncus curtisii and insights into structural dynamics. *International journal of biological macromolecules*. May 1 2020;150:1027-1036. doi:10.1016/j.ijbiomac.2019.10.107
276. Schwebke JR, Morgan SC, Hillier SL. Humoral antibody to Mobiluncus curtisii, a potential serological marker for bacterial vaginosis. *Clinical and diagnostic laboratory immunology*. Sep 1996;3(5):567-9. doi:10.1128/cdli.3.5.567-569.1996
277. Meltzer MC, Desmond RA, Schwebke JR. Association of Mobiluncus curtisii with recurrence of bacterial vaginosis. *Sexually transmitted diseases*. Jun 2008;35(6):611-3. doi:10.1097/OLQ.0b013e318167b105
278. Andrade López AC, Bande Fernández JJ, Astudillo Cortés E, Durá Gúrpide B, Rodríguez Suárez C. Aureimonas altamirensis: The first case of peritonitis on peritoneal dialysis. *Nefrologia*. Nov-Dec 2019;39(6):675-677. Aureimonas altamirensis: primer caso de peritonitis en diálisis peritoneal. doi:10.1016/j.nefro.2019.03.007
279. Schröttner P, Rudolph WW, Taube F, Gunzer F. First report on the isolation of Aureimonas altamirensis from a patient with peritonitis. *International journal of infectious diseases : IJID : official publication of the International Society for Infectious Diseases*. Dec 2014;29:71-3. doi:10.1016/j.ijid.2014.09.006
280. Kim N, Hwang JH, Cho YG, Kim DS, Lee HS, Lee J. First Case of Aureimonas altamirensis Bacteremia in Korea. *Annals of laboratory medicine*. Nov 2019;39(6):587-589. doi:10.3343/alm.2019.39.6.587
281. Eshaghi A, Shahinas D, Patel SN, Kus JV. First draft genome sequence of Aureimonas altamirensis, isolated from patient blood culture. *FEMS Microbiol Lett*. Mar 2015;362(6)doi:10.1093/femsle/fnv016
282. St Geme JW, 3rd. The pathogenesis of nontypable Haemophilus influenzae otitis media. *Vaccine*. Dec 8 2000;19 Suppl 1:S41-50. doi:10.1016/s0264-410x(00)00277-2

283. St Geme JW, 3rd. Insights into the mechanism of respiratory tract colonization by nontypable Haemophilus influenzae. *The Pediatric infectious disease journal*. Oct 1997;16(10):931-5. doi:10.1097/00006454-199710000-00005
284. Stewart L, Sinha S, Madsen PJ, Glaser L, Chen HI, Culyba MJ. Spinal epidural abscess caused by Gardnerella vaginalis and Prevotella amnii. *Infectious diseases in clinical practice (Baltimore, Md)*. 2018 Jul 2018;26(4):237-239. doi:10.1097/ipc.0000000000000565
285. Olate-Pérez A, Díaz-Céspedes RA, Ruíz-Del-Río N, Hernández-Pérez D, Duch-Samper A. Brevibacterium casei endophthalmitis after intravitreal dexamethasone implant. *Archivos de la Sociedad Espanola de Oftalmologia*. 2020 Dec 03 2020;doi:10.1016/j.oftal.2020.09.007
286. Joshi S, Misra R, Kirolikar S, Mushrif S. Catheter-related Brevibacterium casei bloodstream infection in a child with aplastic anaemia. *Indian journal of medical microbiology*. 2020 Apr-Jun 2020;38(2):226-228. doi:10.4103/ijmm.IJMM_20_292
287. Dauby N, Martiny D, Busson L, et al. Atopobium vaginae intrapartum bacteremia: A case report with a literature review. *Anaerobe*. 2019 Oct 2019;59:212-214. doi:10.1016/j.anaerobe.2018.09.010
288. Taillandier P, Roingeard C, Violette J, Leclère F-M, Faivre S. Septic shock caused by Gardnerella vaginalis and Atopobium vaginae. *IDCases*. 2020 2020;21:e00876. doi:10.1016/j.idcr.2020.e00876
289. Monticelli J, Knezevich A, Luzzati R, Bella SD. Clinical management of non-faecium non-faecalis vancomycin-resistant enterococci infection. Focus on Enterococcus gallinarum and Enterococcus casseliflavus/flavescens. *Journal of infection and chemotherapy : official journal of the Japan Society of Chemotherapy*. 2018 Apr 2018;24(4):237-246. doi:10.1016/j.jiac.2018.01.001
290. Bao QD, Liu TX, Xie M, Tian X. Exogenous endophthalmitis caused by Enterococcus casseliflavus: A case report. *World J Clin Cases*. Nov 26 2019;7(22):3904-3911. doi:10.12998/wjcc.v7.i22.3904
291. García-Tornel S, Marqués JC, Tahull JMG, Ullot R, Minguella JM. Bacteroides coagulans osteoarthritis. *Pediatric infectious disease*. 1983 Nov-Dec 1983;2(6):472-4. doi:10.1097/00006454-198311000-00016
292. Merhej V, Falsen E, Raoult D, Roux V. Corynebacterium timonense sp. nov. and Corynebacterium massiliense sp. nov., isolated from human blood and human articular hip fluid. *International journal of systematic and evolutionary microbiology*. 2009 Aug 2009;59(Pt 8):1953-9. doi:10.1099/ijs.0.005827-0
293. Baumgartner A, Thurnheer T, Lüthi-Schaller H, Gmür R, Belibasakis GN. The phylum Synergistetes in gingivitis and necrotizing ulcerative gingivitis. *Journal of medical microbiology*. Nov 2012;61(Pt 11):1600-1609. doi:10.1099/jmm.0.047456-0
294. McCracken BA, Nathalia Garcia M. Phylum Synergistetes in the oral cavity: A possible contributor to periodontal disease. *Anaerobe*. Apr 2021;68:102250. doi:10.1016/j.anaerobe.2020.102250
295. Church DL, Simmon KE, Sporina J, Lloyd T, Gregson DB. Identification by 16S rRNA gene sequencing of Negativicoccus succinicivorans recovered from the blood of a

patient with hemochromatosis and pancreatitis. *Journal of clinical microbiology*. 2011 Aug 2011;49(8):3082-4. doi:10.1128/jcm.01913-10

296. Asif AA, Roy M, Ahmad S. Rare case of Prevotella pleuritidis lung abscess. *BMJ case reports*. 2020 Sep 07 2020;13(9)doi:10.1136/bcr-2020-235960

297. Sakamoto M, Ohkusu K, Masaki T, Kako H, Ezaki T, Benno Y. Prevotella pleuritidis sp. nov., isolated from pleural fluid. *International journal of systematic and evolutionary microbiology*. 2007 Aug 2007;57(Pt 8):1725-1728. doi:10.1099/ijs.0.64885-0

298. Copeland A, Sikorski J, Lapidus A, et al. Complete genome sequence of Atopobium parvulum type strain (IPP 1246). *Standards in genomic sciences*. 2009 Sep 23 2009;1(2):166-73. doi:10.4056/sigs.29547

299. Suzuki J, Sasahara T, Toshima M, Morisawa Y. Peripherally inserted central catheter-related bloodstream infection due to Tsukamurella pulmonis: a case report and literature review. *BMC infectious diseases*. 2017 10 11 2017;17(1):677. doi:10.1186/s12879-017-2796-8

300. Kechker P, Senderovich Y, Ken-Dror S, Laviad-Shitrit S, Halpern M. Tsukamurella pulmonis conjunctivitis in patients with an underlying nasolacrimal duct obstruction - report of two cases. *Access microbiology*. 2021 Feb 2021;3(2):000185. doi:10.1099/acmi.0.000185

301. Jangda U, Upadhyay A, Bagheri F, Patel NR, Mendelson RI. Corynebacterium propinquum: A Rare Cause of Prosthetic Valve Endocarditis. *Case reports in medicine*. 2016 2016;2016:1391789. doi:10.1155/2016/1391789

302. Malkoçoğlu G, Gencer H, Kaya A, Dalgıç N, Bulut ME, Aktaş E. Corynebacterium propinquum bronchopneumonia in a child with ataxia telangiectasia. *The Turkish journal of pediatrics*. 2016 2016;58(5):558-561. doi:10.24953/turkjped.2016.05.018

303. Badenoch PR, O'Daniel LJ, Wise RP, Slattery JA, Mills RA. Corynebacterium propinquum Keratitis Identified Using MALDI-TOF. *Cornea*. 2016 May 2016;35(5):686-7. doi:10.1097/ico.0000000000000787

304. Saïdani M, Kammoun S, Boubaker IB-B, Redjeb SB. Corynebacterium propinquum isolated from a pus collection in a patient with an osteosynthesis of the elbow. *La Tunisie medicale*. 2010 May 2010;88(5):360-2.

305. Babay HA. Pleural effusion due to Corynebacterium propinquum in a patient with squamous cell carcinoma. *Annals of Saudi medicine*. 2001 Sep-Nov 2001;21(5-6):337-9. doi:10.5144/0256-4947.2001.337

306. Stevens DA, Kim KK, Johnson N, Lee J-S, Hamilton JR. Halomonas johnsoniae: review of a medically underappreciated genus of growing human importance. *The American journal of the medical sciences*. 2013 May 2013;345(5):335-8. doi:10.1097/MAJ.0b013e31825600de

307. Frickmann H, Hahn A, Skusa R, et al. Comparison of the etiological relevance of Staphylococcus haemolyticus and Staphylococcus hominis. *European journal of clinical microbiology & infectious diseases : official publication of the European Society of Clinical Microbiology*. 2018 Aug 2018;37(8):1539-1545. doi:10.1007/s10096-018-3282-y

308. Legaria MC, Nastro M, Camporro J, et al. Peptostreptococcus anaerobius: Pathogenicity, identification, and antimicrobial susceptibility. Review of monobacterial infections and addition of a case of urinary tract infection directly identified from a urine sample by MALDI-TOF MS. *Anaerobe*. 2021 Oct 07 2021;72:102461.

doi:10.1016/j.anaerobe.2021.102461
309. Cone LA, Battista BA, Shaeffer CW, Jr. Endocarditis due to Peptostreptococcus anaerobius: case report and literature review of peptostreptococcal endocarditis. *The Journal of heart valve disease*. May 2003;12(3):411-3.
310. Chen YL, Tsai SH, Hsu KC, Chen CS, Hsu CW. Primary sternal osteomyelitis due to Peptostreptococcus anaerobius. *Infection*. Apr 2012;40(2):195-7. doi:10.1007/s15010-011-0171-z
311. Tandon A, Tay-Kearney ML, Metcalf C, McAllister L. Bacillus circulans endophthalmitis. *Clin Exp Ophthalmol*. Apr 2001;29(2):92-3.
312. Russo A, Tarantino U, d'Ettorre G, et al. First report of spondylodiscitis caused by Bacillus circulans in an immunocompetent patient: Clinical case and review of the literature. *IDCases*. 2021;23:e01058. doi:10.1016/j.idcr.2021.e01058
313. Alebouyeh M, Gooran Orimi P, Azimi-Rad M, et al. Fatal sepsis by Bacillus circulans in an immunocompromised patient. *Iran J Microbiol*. Sep 2011;3(3):156-8.
314. Gurol Y, Kipritci Z, Selcuk NA, Koc Y, Kocagoz S. Bacillus circulans paracardiac infection in non-hodgkin lymphoma--a case report. *Prague Med Rep*. 2008;109(1):19-22.
315. Sanyal SK, Karmaker M, Sultana M, Hossain MA. Association of Bacillus circulans with non-diabetic foot infection in Bangladeshi patient. *Indian J Med Microbiol*. Oct-Dec 2015;33(4):606-8. doi:10.4103/0255-0857.167346
316. Goudswaard WB, Dammer MH, Hol C. Bacillus circulans infection of a proximal interphalangeal joint after a clenched-fist injury caused by human teeth. *Eur J Clin Microbiol Infect Dis*. Nov 1995;14(11):1015-16. doi:10.1007/bf01691387
317. Espinoza-Monje M, Campos J, Alvarez Villamil E, et al. Characterization of Weissella viridescens UCO-SMC3 as a Potential Probiotic for the Skin: Its Beneficial Role in the Pathogenesis of Acne Vulgaris. *Microorganisms*. Jul 13 2021;9(7)doi:10.3390/microorganisms9071486
318. Meservey A, Sullivan A, Wu C, Lantos PM. Staphylococcus sciuri peritonitis in a patient on peritoneal dialysis. *Zoonoses Public Health*. Feb 2020;67(1):93-95. doi:10.1111/zph.12664
319. Shittu A, Lin J, Morrison D, Kolawole D. Isolation and molecular characterization of multiresistant Staphylococcus sciuri and Staphylococcus haemolyticus associated with skin and soft-tissue infections. *J Med Microbiol*. Jan 2004;53(Pt 1):51-55. doi:10.1099/jmm.0.05294-0
320. Stepanovic S, Jezek P, Vukovic D, Dakic I, Petrás P. Isolation of members of the Staphylococcus sciuri group from urine and their relationship to urinary tract infections. *J Clin Microbiol*. Nov 2003;41(11):5262-4. doi:10.1128/jcm.41.11.5262-5264.2003
321. Stepanović S, Jezek P, Dakić I, Vuković D, Seifert L. Staphylococcus sciuri: an unusual cause of pelvic inflammatory disease. *Int J STD AIDS*. Jun 2005;16(6):452-3. doi:10.1258/0956462054093999
322. Horii T, Suzuki Y, Kimura T, Kanno T, Maekawa M. Intravenous catheter-related septic shock caused by Staphylococcus sciuri and Escherichia vulneris. *Scandinavian journal of infectious diseases*. 2001;33(12):930-2. doi:10.1080/00365540110076750

323. Coimbra DG, Almeida AG, Jùnior JB, et al. Wound infection by multiresistant Staphylococcus sciuri identified by molecular methods. *New Microbiol*. Oct 2011;34(4):425-7.
324. Koçoğlu E, Karabay O. [Catheter associated Staphylococcus sciuri sepsis in a patient with acute myeloid leukemia]. *Mikrobiyol Bul*. Oct 2006;40(4):397-400. Akut myelositik lösemili bir olguda kateter ile ilişkili Staphylococcus sciuri sepsisi.
325. Hedin G, Widerström M. Endocarditis due to Staphylococcus sciuri. *Eur J Clin Microbiol Infect Dis*. Sep 1998;17(9):673-5. doi:10.1007/bf01708356
326. Seo JY, Yeom JS, Ko KS. Actinomyces cardiffensis septicemia: a case report. *Diagn Microbiol Infect Dis*. May 2012;73(1):86-8. doi:10.1016/j.diagmicrobio.2012.02.012
327. Mahalakshmi K, Krishnan P, Chandrasekaran SC. Detection of Tannerella forsythia bspA and prtH genotypes among periodontitis patients and healthy subjects-A case-Control study. *Arch Oral Biol*. Dec 2018;96:178-181. doi:10.1016/j.archoralbio.2018.09.012
328. Barash A, Chou TY. Moraxella atlantae keratitis presenting with an infectious ring ulcer. *Am J Ophthalmol Case Rep*. Sep 2017;7:62-65. doi:10.1016/j.ajoc.2017.06.003
329. Carbonell-Muñoz C, Zhilina S, Barbosa-Ventura A, et al. [Isolation of Moraxella atlantae on the valve of a patient with native infective endocarditis]. *Rev Esp Quimioter*. Dec 2018;31(6):550-551. Aislamiento de Moraxella atlantae en la válvula nativa de un paciente con endocarditis infecciosa.
330. Buchman AL, Pickett MJ. Moraxella atlantae bacteraemia in a patient with systemic lupus erythematosis. *J Infect*. Sep 1991;23(2):197-9. doi:10.1016/0163-4453(91)92335-3
331. Yin X, Liang Y, Zeng L, Chen S. Bacteremia and Bone Marrow Infection Caused by Moraxella Atlantae in an Elderly Patient with Pneumonia. *Clin Lab*. Dec 1 2016;62(12):2419-2422. doi:10.7754/Clin.Lab.2016.160527
332. García-Fernández-Bravo I, Ordieres-Ortega L, Braojos-Sánchez F, Demelo-Rodríguez P. Respiratory sepsis due to Moraxella atlantae: Utility of mass spectrometry to identify rare species. *Enferm Infecc Microbiol Clin*. Apr 2017;35(4):265-266. Sepsis respiratoria por Moraxella atlantae: utilidad de la espectrometría de masas en la identificación de especies poco frecuentes. doi:10.1016/j.eimc.2016.03.005
333. Mormeneo Bayo S, Palacián Ruíz MP, Asin Samper U, Millán Lou MI, Pascual Catalán A, Villuendas Usón MC. Pacemaker-induced endocarditis by Gordonia bronchialis. *Enferm Infecc Microbiol Clin (Engl Ed)*. Jan 11 2021;doi:10.1016/j.eimc.2020.11.010
334. Choi R, Strnad L, Flaxel CJ, Lauer AK, Suhler EB. Gordonia bronchialis-Associated Endophthalmitis, Oregon, USA. *Emerg Infect Dis*. May 2019;25(5):1017-1019. doi:10.3201/eid2505.180340
335. Johnson JA, Onderdonk AB, Cosimi LA, et al. Gordonia bronchialis bacteremia and pleural infection: case report and review of the literature. *J Clin Microbiol*. Apr 2011;49(4):1662-6. doi:10.1128/jcm.02121-10
336. Wright SN, Gerry JS, Busowski MT, et al. Gordonia bronchialis sternal wound infection in 3 patients following open heart surgery: intraoperative transmission from a healthcare worker. *Infect Control Hosp Epidemiol*. Dec 2012;33(12):1238-41. doi:10.1086/668441

337. Chang JH, Ji M, Hong HL, et al. Sternal Osteomyelitis Caused by Gordonia bronchialis after Open-Heart Surgery. *Infect Chemother*. Jun 2014;46(2):110-4. doi:10.3947/ic.2014.46.2.110
338. Bruno V, Tjon J, Lin S, et al. Peritoneal dialysis-related peritonitis caused by Gordonia bronchialis: first pediatric report. *Pediatr Nephrol*. Jan 2022;37(1):217-220. doi:10.1007/s00467-021-05313-3
339. Choi ME, Jung CJ, Won CH, et al. Case report of cutaneous nodule caused by Gordonia bronchialis in an immunocompetent patient after receiving acupuncture. *J Dermatol*. Apr 2019;46(4):343-346. doi:10.1111/1346-8138.14785
340. Bartolomé-Álvarez J, Sáez-Nieto JA, Escudero-Jiménez A, et al. Cutaneous abscess due to Gordonia bronchialis: case report and literature review. *Rev Esp Quimioter*. Jun 2016;29(3):170-3.
341. Sng LH, Koh TH, Toney SR, Floyd M, Butler WR, Tan BH. Bacteremia caused by Gordonia bronchialis in a patient with sequestrated lung. *J Clin Microbiol*. Jun 2004;42(6):2870-1. doi:10.1128/jcm.42.6.2870-2871.2004
342. Werno AM, Anderson TP, Chambers ST, Laird HM, Murdoch DR. Recurrent breast abscess caused by Gordonia bronchialis in an immunocompetent patient. *J Clin Microbiol*. Jun 2005;43(6):3009-10. doi:10.1128/jcm.43.6.3009-3010.2005
343. Funke G, Osorio CR, Frei R, Riegel P, Collins MD. Corynebacterium confusum sp. nov., isolated from human clinical specimens. *International journal of systematic bacteriology*. Oct 1998;48 Pt 4:1291-6. doi:10.1099/00207713-48-4-1291
344. Kressirer CA, Smith DJ, King WF, Dobeck JM, Starr JR, Tanner ACR. Scardovia wiggsiae and its potential role as a caries pathogen. *J Oral Biosci*. Aug 2017;59(3):135-141. doi:10.1016/j.job.2017.05.002
345. Posteraro P, De Maio F, Menchinelli G, et al. First bloodstream infection caused by Prevotella copri in a heart failure elderly patient with Prevotella-dominated gut microbiota: a case report. *Gut Pathog*. 2019;11:44. doi:10.1186/s13099-019-0325-6
346. Wyss C, Choi BK, Schüpbach P, Guggenheim B, Göbel UB. Treponema amylovorum sp. nov., a saccharolytic spirochete of medium size isolated from an advanced human periodontal lesion. *International journal of systematic bacteriology*. Jul 1997;47(3):842-5. doi:10.1099/00207713-47-3-842
347. Forrester JD, Spain DA. Clostridium ramosum bacteremia: case report and literature review. *Surg Infect (Larchmt)*. Jun 2014;15(3):343-6. doi:10.1089/sur.2012.240
348. Kozaki S, Miyamoto S, Uchida K, et al. Infected thoracic aortic aneurysm caused by Clostridium ramosum: A case report. *J Cardiol Cases*. Sep 2019;20(3):103-105. doi:10.1016/j.jccase.2019.06.005
349. García-Jiménez A, Prim N, Crusi X, Benito N. Septic arthritis due to Clostridium ramosum. *Semin Arthritis Rheum*. Apr 2016;45(5):617-20. doi:10.1016/j.semarthrit.2015.09.009
350. Miret C, Fernández-Sola J, Molleda M, De Dios A. [Clostridium ramosum: a rare cause of brain abscess]. *An Med Interna*. Jul 1998;15(7):392-3. Clostridium ramosun: una

causa excepcional de absceso cerebral.
351. Lavigne JP, Bouziges N, Sotto A, Leroux JL, Michaux-Charachon S. Spondylodiscitis due to Clostridium ramosum infection in an immunocompetent elderly patient. *J Clin Microbiol*. May 2003;41(5):2223-6. doi:10.1128/jcm.41.5.2223-2226.2003
352. Ikeda M, Kobayashi T, Suzuki T, et al. Propionimicrobium lymphophilum and Actinotignum schaalii bacteraemia: a case report. *New Microbes New Infect*. Jul 2017;18:18-21. doi:10.1016/j.nmni.2017.03.004
353. Williams GD. Two Cases of Urinary Tract Infection Caused by Propionimicrobium lymphophilum. *J Clin Microbiol*. Sep 2015;53(9):3077-80. doi:10.1128/jcm.00438-15
354. Rodríguez Fernández L, Martín Guerra JM, Gil González I. Bacteremia by Nocardia farcinica. *Med Clin (Barc)*. Jun 26 2020;154(12):520-521. Bacteriemia por Nocardia farcinica. doi:10.1016/j.medcli.2019.02.016
355. Verner A, Durrani A, Kowalski RP, Jhanji V. A Case of Nocardia farcinica Keratitis in a Pediatric Contact Lens Wearer. *Eye Contact Lens*. Mar 2020;46(2):e11-e12. doi:10.1097/icl.0000000000000594
356. Wang H, Cheng F, Dong XT, Li XF, Wang YJ, Hao MJ. [Antibiotic analysis and whole genome sequencing of two nocardia farcinica strains causing joint infection]. *Zhonghua Yu Fang Yi Xue Za Zhi*. Dec 6 2020;54(12):1441-1447. doi:10.3760/cma.j.cn112150-20200922-01226
357. Kim J, Kang M, Kim J, et al. A Case of Nocardia farcinica Pneumonia and Mediastinitis in an Immunocompetent Patient. *Tuberc Respir Dis (Seoul)*. Apr 2016;79(2):101-3. doi:10.4046/trd.2016.79.2.101
358. Wang A, Xu Q, Wang Y, Liao H. Orbital and intracranial Nocardia farcinica infection caused by trauma to the orbit: a case report. *BMC Infect Dis*. Nov 8 2019;19(1):953. doi:10.1186/s12879-019-4605-z
359. García Callejo J. Psoas and neck abscess by Nocardia farcinica. *Cir Esp (Engl Ed)*. Feb 2019;97(2):111-112. Absceso de psoas y cuello por Nocardia farcinica. doi:10.1016/j.ciresp.2018.07.005
360. Sirijatuphat R, Niltwat S, Tiangtam O, Tungsubutra W. Purulent pericarditis and cardiac tamponade caused by Nocardia farcinica in a nephrotic syndrome patient. *Intern Med*. 2013;52(19):2231-5. doi:10.2169/internalmedicine.52.0453
361. Kumar VA, Augustine D, Panikar D, et al. Nocardia farcinica brain abscess: epidemiology, pathophysiology, and literature review. *Surg Infect (Larchmt)*. Oct 2014;15(5):640-6. doi:10.1089/sur.2012.205
362. Nasri E, Fakhim H, Barac A, et al. Nocardia farcinica meningitis in a patient with high-grade astrocytoma. *J Infect Dev Ctries*. Sep 30 2019;13(9):854-857. doi:10.3855/jidc.11582
363. Brown JM, Cowley KD, Manninen KI, McNeil MM. Phenotypic and molecular epidemiologic evaluation of a Nocardia farcinica mastitis epizootic. *Vet Microbiol*. Nov 15 2007;125(1-2):66-72. doi:10.1016/j.vetmic.2007.04.044

364. Burdová M, Donátová K, Mahelková G, Chrenková V, Dotřelová D. Post-traumatic exogenous endophthalmitis caused by Nocardia farcinica. *J Ophthalmic Inflamm Infect*. Jun 1 2021;11(1):16. doi:10.1186/s12348-021-00245-3
365. Graat HC, Van Ooij A, Day GA, McPhee IB. Nocardia farcinica spinal osteomyelitis. *Spine (Phila Pa 1976)*. May 15 2002;27(10):E253-7. doi:10.1097/00007632-200205150-00021
366. Scorey H, Daniel S. Nocardia farcinica bacteraemia presenting as a prostate abscess. *IDCases*. 2016;5:24-6. doi:10.1016/j.idcr.2016.06.001
367. Sanchez Eluchans N, Barberis C, Cittadini R, et al. [Corynebacterium kroppenstedtii breast infections: Report of four cases]. *Rev Argent Microbiol*. Oct-Dec 2021;53(4):304-308. Infecciones mamarias por Corynebacterium kroppenstedtii: comunicación de 4 casos. doi:10.1016/j.ram.2021.01.002
368. Saraiya N, Corpuz M. Corynebacterium kroppenstedtii: a challenging culprit in breast abscesses and granulomatous mastitis. *Curr Opin Obstet Gynecol*. Oct 2019;31(5):325-332. doi:10.1097/gco.0000000000000541
369. Vaidya A, Sarbajna T, Kakizaki H, Takahashi Y. Corynebacterium kroppenstedtii as a pathogen of a Jones tube infection following conjunctivo-dacryocystorhinostomy. *Orbit*. Dec 2020;39(6):415-417. doi:10.1080/01676830.2019.1688359
370. Roth S, Ehrlich T, Schäfers HJ, Becker SL. Late-onset native valve endocarditis caused by Corynebacterium kroppenstedtii. *Int J Infect Dis*. Dec 2020;101:1-3. doi:10.1016/j.ijid.2020.09.023
371. Matsuoka T, Shimizu T, Minagawa T, et al. First case of an invasive Bacteroides dorei infection detected in a patient with a mycotic aortic aneurysm-raising a rebellion of major indigenous bacteria in humans: a case report and review. *BMC Infect Dis*. Jun 30 2021;21(1):625. doi:10.1186/s12879-021-06345-8
372. Sárvári KP, Sántha D, Kovács R, et al. Six cases of Solobacterium moorei isolated alone or in mixed culture in Hungary and comparison with previously published cases. *Anaerobe*. Oct 2020;65:102241. doi:10.1016/j.anaerobe.2020.102241
373. Liu WJ, Xiao M, Yi J, Li Y, Kudinha T, Xu YC. First case report of bacteremia caused by Solobacterium moorei in China, and literature review. *BMC Infect Dis*. Aug 20 2019;19(1):730. doi:10.1186/s12879-019-4359-7
374. Martin CA, Wijesurendra RS, Borland CD, Karas JA. Femoral vein thrombophlebitis and septic pulmonary embolism due to a mixed anaerobic infection including Solobacterium moorei: a case report. *J Med Case Rep*. Jul 2 2007;1:40. doi:10.1186/1752-1947-1-40
375. Detry G, Pierard D, Vandoorslaer K, Wauters G, Avesani V, Glupczynski Y. Septicemia due to Solobacterium moorei in a patient with multiple myeloma. *Anaerobe*. Jun 2006;12(3):160-2. doi:10.1016/j.anaerobe.2006.04.002
376. Tan EM, Tande AJ, Osmon DR, Wilson JW. Mycobacterium iranicum septic arthritis and tenosynovitis. *J Clin Tuberc Other Mycobact Dis*. Aug 2017;8:16-18. doi:10.1016/j.jctube.2017.05.003
377. Grandjean Lapierre S, Toro A, Drancourt M. Mycobacterium iranicum bacteremia and hemophagocytic lymphohistiocytosis: a case report. *BMC Res Notes*. Aug 8

2017;10(1):372. doi:10.1186/s13104-017-2684-8
378. Becker SL, Halfmann A, Schilling L, et al. Mycobacterium iranicum infection in a patient with fish tank granuloma: a first case report. *Eur J Dermatol*. Apr 1 2018;28(2):238-239. doi:10.1684/ejd.2017.3196
379. Inagaki K, Mizutani M, Nagahara Y, et al. Successful Treatment of Peritoneal Dialysis-related Peritonitis due to Mycobacterium iranicum. *Intern Med*. 2016;55(14):1929-31. doi:10.2169/internalmedicine.55.5219
380. Tsukamura M, Mizuno S, Gane NF, Mills A, King L. Mycobacterium rhodesiae sp. nov. A new species of rapid-growing scotochromogenic mycobacteria. *Japanese journal of microbiology*. Sep 1971;15(5):407-16. doi:10.1111/j.1348-0421.1971.tb00598.x
381. Curry EM, Yehia M, Roberts S. CAPD peritonitis caused by Mycobacterium rhodesiae. *Peritoneal dialysis international : journal of the International Society for Peritoneal Dialysis*. Jan-Feb 2008;28(1):97-9.
382. Cools P, Oyaert M, Vaneechoutte M, De Laere E, Vervaeke S. Atopobium deltae sp. nov., isolated from the blood of a patient with Fournier's gangrene. *International journal of systematic and evolutionary microbiology*. Sep 2014;64(Pt 9):3140-3145. doi:10.1099/ijs.0.065243-0
383. Oyaert M, Cools P, Breyne J, et al. Sepsis with an Atopobium-like species in a patient with Fournier's gangrene. *Journal of clinical microbiology*. Jan 2014;52(1):364-6. doi:10.1128/jcm.02310-13
384. Yang J, Wang P, Liu T, et al. Involvement of mucosal flora and enterochromaffin cells of the caecum and descending colon in diarrhoea-predominant irritable bowel syndrome. *BMC microbiology*. Nov 13 2021;21(1):316. doi:10.1186/s12866-021-02380-2
385. Kaczmarczyk M, Löber U, Adamek K, et al. The gut microbiota is associated with the small intestinal paracellular permeability and the development of the immune system in healthy children during the first two years of life. *Journal of translational medicine*. Apr 28 2021;19(1):177. doi:10.1186/s12967-021-02839-w
386. Sato N, Kakuta M, Hasegawa T, et al. Metagenomic profiling of gut microbiome in early chronic kidney disease. *Nephrology, dialysis, transplantation : official publication of the European Dialysis and Transplant Association - European Renal Association*. Aug 27 2021;36(9):1675-1684. doi:10.1093/ndt/gfaa122
387. Jiang W, Yu X, Kosik RO, et al. Gut Microbiota May Play a Significant Role in the Pathogenesis of Graves' Disease. *Thyroid : official journal of the American Thyroid Association*. May 2021;31(5):810-820. doi:10.1089/thy.2020.0193
388. Wang Y, Ye X, Ding D, Lu Y. Characteristics of the intestinal flora in patients with peripheral neuropathy associated with type 2 diabetes. *The Journal of international medical research*. Sep 2020;48(9):300060520936806. doi:10.1177/0300060520936806
389. Tian R, Liu H, Feng S, et al. Gut microbiota dysbiosis in stable coronary artery disease combined with type 2 diabetes mellitus influences cardiovascular prognosis. *Nutrition, metabolism, and cardiovascular diseases : NMCD*. May 6 2021;31(5):1454-1466. doi:10.1016/j.numecd.2021.01.007
390. Palmieri O, Castellana S, Biscaglia G, et al. Microbiome Analysis of Mucosal Ileoanal Pouch in Ulcerative Colitis Patients Revealed Impairment of the Pouches

Immunometabolites. *Cells*. Nov 19 2021;10(11)doi:10.3390/cells10113243
391. Zinkernagel MS, Zysset-Burri DC, Keller I, et al. Association of the Intestinal Microbiome with the Development of Neovascular Age-Related Macular Degeneration. *Scientific reports*. Jan 17 2017;7:40826. doi:10.1038/srep40826
392. Botelho PB, Ferreira MVR, Araújo AM, Mendes MM, Nakano EY. Effect of multispecies probiotic on gut microbiota composition in individuals with intestinal constipation: A double-blind, placebo-controlled randomized trial. *Nutrition (Burbank, Los Angeles County, Calif)*. Oct 2020;78:110890. doi:10.1016/j.nut.2020.110890
393. Laue HE, Korrick SA, Baker ER, Karagas MR, Madan JC. Prospective associations of the infant gut microbiome and microbial function with social behaviors related to autism at age 3 years. *Scientific reports*. Sep 23 2020;10(1):15515. doi:10.1038/s41598-020-72386-9
394. Lyra A, Rinttilä T, Nikkilä J, et al. Diarrhoea-predominant irritable bowel syndrome distinguishable by 16S rRNA gene phylotype quantification. *World journal of gastroenterology*. Dec 21 2009;15(47):5936-45. doi:10.3748/wjg.15.5936
395. Chen Y, Lin H, Cole M, et al. Signature changes in gut microbiome are associated with increased susceptibility to HIV-1 infection in MSM. *Microbiome*. Dec 9 2021;9(1):237. doi:10.1186/s40168-021-01168-w
396. Halmos EP, Christophersen CT, Bird AR, Shepherd SJ, Muir JG, Gibson PR. Consistent Prebiotic Effect on Gut Microbiota With Altered FODMAP Intake in Patients with Crohn's Disease: A Randomised, Controlled Cross-Over Trial of Well-Defined Diets. *Clinical and translational gastroenterology*. Apr 14 2016;7(4):e164. doi:10.1038/ctg.2016.22
397. Rôças IN, Siqueira JF, Jr. Prevalence of new candidate pathogens Prevotella baroniae, Prevotella multisaccharivorax and as-yet-uncultivated Bacteroidetes clone X083 in primary endodontic infections. *Journal of endodontics*. Oct 2009;35(10):1359-62. doi:10.1016/j.joen.2009.05.033
398. Sun B, Liu B, Gao X, Xing K, Xie L, Guo T. Metagenomic Analysis of Saliva Reveals Disease-Associated Microbiotas in Patients With Periodontitis and Crohn's Disease-Associated Periodontitis. *Frontiers in cellular and infection microbiology*. 2021;11:719411. doi:10.3389/fcimb.2021.719411
399. Rôças IN, Alves FR, Santos AL, Rosado AS, Siqueira JF, Jr. Apical root canal microbiota as determined by reverse-capture checkerboard analysis of cryogenically ground root samples from teeth with apical periodontitis. *Journal of endodontics*. Oct 2010;36(10):1617-21. doi:10.1016/j.joen.2010.07.001
400. Onorati I, Guiraudet P, Billard-Pomares T, Martinod E. A recurrent lung abscess caused by delayed diagnosis of unique co-infection with Abiotrophia defectiva. *Interactive cardiovascular and thoracic surgery*. Dec 7 2020;31(6):909-911. doi:10.1093/icvts/ivaa212
401. Gupta G, Chaudhary M, Khunt A, Shah V, Shah MM. An unreported case of Streptococcus cristatus septic arthritis of wrist in a neonate. *Journal of clinical orthopaedics and trauma*. Mar-Apr 2020;11(2):328-331. doi:10.1016/j.jcot.2019.02.002
402. Matthys C, Claeys G, Verschraegen G, et al. Streptococcus cristatus isolated from a resected heart valve and blood cultures: case reports and application of phenotypic and genotypic techniques for identification. *Acta clinica Belgica*. Jul-Aug 2006;61(4):196-200. doi:10.1179/acb.2006.034
403. Lieberman JA, Naureckas Li C, Lamb GS, et al. Case Report: Comparison of Plasma Metagenomics to Bacterial PCR in a Case of Prosthetic Valve Endocarditis. *Frontiers in*

pediatrics. 2020;8:575674. doi:10.3389/fped.2020.575674
404. Kanasi E, Dewhirst FE, Chalmers NI, et al. Clonal analysis of the microbiota of severe early childhood caries. *Caries research*. 2010;44(5):485-97. doi:10.1159/000320158
405. Dzidic M, Collado MC, Abrahamsson T, et al. Oral microbiome development during childhood: an ecological succession influenced by postnatal factors and associated with tooth decay. *The ISME journal*. Sep 2018;12(9):2292-2306. doi:10.1038/s41396-018-0204-z
406. Tanner AC, Mathney JM, Kent RL, et al. Cultivable anaerobic microbiota of severe early childhood caries. *Journal of clinical microbiology*. Apr 2011;49(4):1464-74. doi:10.1128/jcm.02427-10
407. Jones AL, Koerner RJ, Natarajan S, Perry JD, Goodfellow M. Dietzia papillomatosis sp. nov., a novel actinomycete isolated from the skin of an immunocompetent patient with confluent and reticulated papillomatosis. *International journal of systematic and evolutionary microbiology*. Jan 2008;58(Pt 1):68-72. doi:10.1099/ijs.0.65178-0
408. Jimenez Flores E, Tian S, Sizova M, Epstein SS, Lamont RJ, Uriarte SM. Peptoanaerobacter stomatis Primes Human Neutrophils and Induces Granule Exocytosis. *Infection and immunity*. Jul 2017;85(7)doi:10.1128/iai.01043-16
409. Shinha T. Cellulitis and Bacteremia due to Neisseria weaveri following a dog bite. *IDCases*. 2018;12:56-57. doi:10.1016/j.idcr.2018.03.008
410. Eiros JM, Domínguez-Gil M, Pellicer JL. [Cellulitis by Neisseria weaveri after a dog bite]. *Revista espanola de quimioterapia : publicacion oficial de la Sociedad Espanola de Quimioterapia*. Dec 2015;28(6):322-3. Celulitis por Neisseria weaveri tras mordedura de perro.
411. Kocyigit I, Unal A, Sipahioglu M, Tokgoz B, Oymak O, Utas C. Peritoneal dialysis-related peritonitis due to Neisseria weaveri: the first case report. *Peritoneal dialysis international : journal of the International Society for Peritoneal Dialysis*. Jan-Feb 2010;30(1):116-7. doi:10.3747/pdi.2008.00039
412. Stricker RB, Pompilio KJ, Axelrod JL, Kochman RS, Newman JC. Neisseria subflava endophthalmitis. *American journal of ophthalmology*. Sep 1982;94(3):423-4. doi:10.1016/0002-9394(82)90380-4
413. Rivacoba MC, Izquierdo G, Zenteno N, Porte L. [Neisseria subflava bacteremia in newborns: case report and review of the literature]. *Revista chilena de infectologia : organo oficial de la Sociedad Chilena de Infectologia*. Aug 2017;34(4):389-392. Bacteriemia por Neisseria subflava en un recién nacido. doi:10.4067/s0716-10182017000400389
414. Uwamino Y, Sugita K, Iwasaki E, et al. The First Case Report of Acute Cholangitis and Bacteremia Due to Neisseria subflava. *Internal medicine (Tokyo, Japan)*. 2017;56(2):221-223. doi:10.2169/internalmedicine.56.7482
415. Tena Gómez D, Carranza González R, Torres Cañadillas C, Manrique González E, Garrido Palomo R. [Bacteremia due to Neisseria subflava biovar flava]. *Anales de pediatria (Barcelona, Spain : 2003)*. Aug 2004;61(2):188-9. Bacteriemia por Neisseria subflava biovar flava. doi:10.1016/s1695-4033(04)78383-6
416. Demmler GJ, Couch RS, Taber LH. Neisseria subflava bacteremia and meningitis in a child: report of a case and review of the literature. *Pediatric infectious disease*. May-Jun 1985;4(3):286-8. doi:10.1097/00006454-198505000-00016

417. Pollack S, Mogtader A, Lange M. Neisseria subflava endocarditis. Case report and review of the literature. *The American journal of medicine*. Apr 1984;76(4):752-8. doi:10.1016/0002-9343(84)90311-5
418. Chong Y, Song KS, Lee SY. Neisseria subflava infections--bacteriological aspects of two cases. *Yonsei medical journal*. 1975;16(1):44-9. doi:10.3349/ymj.1975.16.1.44
419. Bullman S, Corcoran D, O'Leary J, Lucey B, Byrne D, Sleator RD. Campylobacter ureolyticus: an emerging gastrointestinal pathogen? *FEMS immunology and medical microbiology*. Mar 2011;61(2):228-30. doi:10.1111/j.1574-695X.2010.00760.x
420. O'Donovan D, Corcoran GD, Lucey B, Sleator RD. Campylobacter ureolyticus: a portrait of the pathogen. *Virulence*. May 15 2014;5(4):498-506. doi:10.4161/viru.28776
421. Hatanaka N, Shimizu A, Somroop S, et al. High Prevalence of Campylobacter ureolyticus in Stool Specimens of Children with Diarrhea in Japan. *Japanese journal of infectious diseases*. Jul 24 2017;70(4):455-457. doi:10.7883/yoken.JJID.2016.428
422. Burgos-Portugal JA, Kaakoush NO, Raftery MJ, Mitchell HM. Pathogenic potential of Campylobacter ureolyticus. *Infection and immunity*. Feb 2012;80(2):883-90. doi:10.1128/iai.06031-11
423. Serichantalergs O, Ruekit S, Pandey P, et al. Incidence of Campylobacter concisus and C. ureolyticus in traveler's diarrhea cases and asymptomatic controls in Nepal and Thailand. *Gut pathogens*. 2017;9:47. doi:10.1186/s13099-017-0197-6
424. Bullman S, Lucid A, Corcoran D, Sleator RD, Lucey B. Genomic investigation into strain heterogeneity and pathogenic potential of the emerging gastrointestinal pathogen Campylobacter ureolyticus. *PloS one*. 2013;8(8):e71515. doi:10.1371/journal.pone.0071515
425. O'Doherty A, Koziel M, De Barra L, et al. Development of nalidixic acid amphotericin B vancomycin (NAV) medium for the isolation of Campylobacter ureolyticus from the stools of patients presenting with acute gastroenteritis. *British journal of biomedical science*. 2014;71(1):6-12. doi:10.1080/09674845.2014.11669956
426. Man SM, Kaakoush NO, Leach ST, et al. Host attachment, invasion, and stimulation of proinflammatory cytokines by Campylobacter concisus and other non-Campylobacter jejuni Campylobacter species. *The Journal of infectious diseases*. Dec 15 2010;202(12):1855-65. doi:10.1086/657316
427. Brito F, Zaltman C, Carvalho AT, et al. Subgingival microflora in inflammatory bowel disease patients with untreated periodontitis. *European journal of gastroenterology & hepatology*. Feb 2013;25(2):239-45. doi:10.1097/MEG.0b013e32835a2b70
428. Luo L, Wang C, Shen N, et al. Polymicrobial anaerobic bacterial meningitis secondary to dermal sinus: a case report. *Translational pediatrics*. Nov 2021;10(11):3118-3123. doi:10.21037/tp-21-210
429. Prodger JL, Abraham AG, Tobian AA, et al. Penile bacteria associated with HIV seroconversion, inflammation, and immune cells. *JCI insight*. Apr 22 2021;6(8)doi:10.1172/jci.insight.147363
430. Teixeira L, Avery RK, Iseman M, et al. Mycobacterium llatzerense lung infection in a liver transplant recipient: case report and review of the literature. *American journal of transplantation : official journal of the American Society of Transplantation and the American Society of Transplant Surgeons*. Aug 2013;13(8):2198-200.

doi:10.1111/ajt.12318
431. Cárdenas AM, Gomila M, Lalucat J, Edelstein PH. Abdominal abscess caused by Mycobacterium llatzerense. *Journal of clinical microbiology*. Apr 2014;52(4):1287-9. doi:10.1128/jcm.03525-13
432. Greninger AL, Langelier C, Cunningham G, et al. Two Rapidly Growing Mycobacterial Species Isolated from a Brain Abscess: First Whole-Genome Sequences of Mycobacterium immunogenum and Mycobacterium llatzerense. *Journal of clinical microbiology*. Jul 2015;53(7):2374-7. doi:10.1128/jcm.00402-15
433. Mashima I, Miyoshi-Akiyama T, Tomida J, et al. Draft Genome Sequences of Two Veillonella tobetsuensis Clinical Isolates from Intraoperative Bronchial Fluids of Elderly Patients with Pulmonary Carcinoma. *Microbiology resource announcements*. Sep 19 2019;8(38)doi:10.1128/mra.00397-19
434. Bomholt C, Glaub A, Gravermann K, et al. Whole-Genome Sequence of the Clinical Strain Corynebacterium argentoratense DSM 44202, Isolated from a Human Throat Specimen. *Genome announcements*. Oct 3 2013;1(5)doi:10.1128/genomeA.00793-13
435. Fernández-Natal MI, Soriano F, Acedo A, Hernandez M, Tauch A, Rodríguez-Lázaro D. Draft Genome Sequences of the Two Unrelated Macrolide-Resistant Corynebacterium argentoratense Strains CNM 463/05 and CNM 601/08, Isolated from Patients in the University Hospital of León, Spain. *Genome announcements*. Jul 9 2015;3(4)doi:10.1128/genomeA.00765-15
436. Fernández-Natal I, Sáez-Nieto JA, Rodríguez-Lázaro D, et al. Phenotypic, molecular characterization, antimicrobial susceptibility and draft genome sequence of Corynebacterium argentoratense strains isolated from clinical samples. *New microbes and new infections*. Mar 2016;10:116-21. doi:10.1016/j.nmni.2016.01.007
437. Kania RE, Lamers GE, Vonk MJ, et al. Characterization of mucosal biofilms on human adenoid tissues. *The Laryngoscope*. Jan 2008;118(1):128-34. doi:10.1097/MLG.0b013e318155a464
438. Yan Q, Gu Y, Li X, et al. Alterations of the Gut Microbiome in Hypertension. *Frontiers in cellular and infection microbiology*. 2017;7:381. doi:10.3389/fcimb.2017.00381
439. Chu W, Han Q, Xu J, et al. Metagenomic analysis identified microbiome alterations and pathological association between intestinal microbiota and polycystic ovary syndrome. *Fertility and sterility*. Jun 2020;113(6):1286-1298.e4. doi:10.1016/j.fertnstert.2020.01.027
440. Nomura K, Ishikawa D, Okahara K, et al. Bacteroidetes Species Are Correlated with Disease Activity in Ulcerative Colitis. *Journal of clinical medicine*. Apr 17 2021;10(8)doi:10.3390/jcm10081749
441. Wang K, Zhang Z, Mo ZS, et al. Gut microbiota as prognosis markers for patients with HBV-related acute-on-chronic liver failure. *Gut microbes*. Jan-Dec 2021;13(1):1-15. doi:10.1080/19490976.2021.1921925
442. Zapała B, Stefura T, Wójcik-Pędziwiatr M, et al. Differences in the Composition of Gut Microbiota between Patients with Parkinson's Disease and Healthy Controls: A Cohort Study. *Journal of clinical medicine*. Dec 3 2021;10(23)doi:10.3390/jcm10235698
443. Carlstein C, Marie Søes L, Jørgen Christensen J. Aerococcus christensenii as Part of Severe Polymicrobial Chorioamnionitis in a Pregnant Woman. *The open microbiology*

journal. 2016;10:27-31. doi:10.2174/1874285801610010027
444. Jose A, Cunha BA, Klein NC, Schoch PE. Aerococcus christensenii native aortic valve subacute bacterial endocarditis (SBE) presenting as culture negative endocarditis (CNE) mimicking marantic endocarditis. *Heart & lung : the journal of critical care*. Mar-Apr 2014;43(2):161-3. doi:10.1016/j.hrtlng.2013.11.005
445. Sharara SL, Tayyar R, Kanafani ZA, Kanj SS. HACEK endocarditis: a review. *Expert review of anti-infective therapy*. Jun 2016;14(6):539-45. doi:10.1080/14787210.2016.1184085
446. Avery LM, Felberbaum CB, Hasan M. Ciprofloxacin for the treatment of Cardiobacterium hominis prosthetic valve endocarditis. *IDCases*. 2018;11:77-79. doi:10.1016/j.idcr.2018.01.016
447. Suresh P, Blackwood RA. A pediatric case of cardiobacterium hominis endocarditis. *Infectious disease reports*. Jan 22 2013;5(1):e7. doi:10.4081/idr.2013.e7
448. Shingu M, Ishimaru N, Ohnishi J, et al. Hemolytic Anemia in a Patient with Subacute Bacterial Endocarditis by Cardiobacterium hominis. *Internal medicine (Tokyo, Japan)*. Nov 1 2021;60(21):3489-3495. doi:10.2169/internalmedicine.6186-20
449. Chambers ST, Murdoch D, Morris A, et al. HACEK infective endocarditis: characteristics and outcomes from a large, multi-national cohort. *PloS one*. 2013;8(5):e63181. doi:10.1371/journal.pone.0063181
450. Donovan J, Hatcher J, Riddell A, Tiberi S. Back pain, leg swelling and a cardiac arrest: an interesting case of endocarditis. *BMJ case reports*. May 23 2014;2014doi:10.1136/bcr-2013-202215
451. Foster MA, Walls T. High rates of complications following Kingella kingae infective endocarditis in children: a case series and review of the literature. *The Pediatric infectious disease journal*. Jul 2014;33(7):785-6. doi:10.1097/inf.0000000000000303
452. Maaliki N, Verdecia J, Ravi M. Elusive Enterobacter cloacae causing pacemaker endocarditis. *IDCases*. 2021;24:e01149. doi:10.1016/j.idcr.2021.e01149
453. Menegueti MG, Machado-Viana J, Gaspar GG, Nicolini EA, Basile-Filho A, Auxiliadora-Martins M. Ischemic Stroke and Septic Shock After Subacute Endocarditis Caused by Haemophilus parainfluenzae: Case Report. *Journal of clinical medicine research*. Jan 2017;9(1):71-73. doi:10.14740/jocmr2703w
454. Molet L, Revest M, Fournet M, et al. A case of tricuspid valve endocarditis due to Cardiobacterium hominis which emphasizes the shift between the poverty of clinical symptoms and the severity of cardiac damages. *Annales de biologie clinique*. Dec 1 2016;74(6):693-696. Endocardite tricuspide à Cardiobacterium hominis illustrant le paradoxe de la pauvreté des signes cliniques malgré une atteinte valvulaire sévère. doi:10.1684/abc.2016.1187
455. Tagini F, Pillonel T, Asner S, Prod'hom G, Greub G. Draft Genome Sequence of a Cardiobacterium hominis Strain Isolated from Blood Cultures of a Patient with Infective Endocarditis. *Genome announcements*. Sep 22 2016;4(5)doi:10.1128/genomeA.00999-16
456. Wong D, Carson J, Johnson A. Subacute bacterial endocarditis caused by Cardiobacterium hominis: A case report. *The Canadian journal of infectious diseases & medical microbiology = Journal canadien des maladies infectieuses et de la microbiologie medicale*. Jan-Feb 2015;26(1):41-3. doi:10.1155/2015/568750

457. Ducoulombier V, Budzik JF, Dehecq E, Baclet N, Houvenagel E. [Cardiobacterium hominis septic arthritis]. *Medecine et maladies infectieuses*. Mar 2014;44(3):129-31. Arthrite septique à Cardiobacterium hominis. doi:10.1016/j.medmal.2014.01.004
458. Salinas J, Irígoin A, Calvo M, Concha C, Ardiles L. [Cardiobacterium hominis pericarditis: an unusual case]. *Revista chilena de infectologia : organo oficial de la Sociedad Chilena de Infectologia*. Dec 2016;33(6):691-695. Pericarditis bacteriana por Cardiobacterium hominis: un caso clínico inusual. doi:10.4067/s0716-10182016000600013
459. Manderwad GP, Kodiganti M, Ali MJ. Cardiobacterium hominis-induced acute dacryocystitis and lacrimal abscess. *Indian journal of ophthalmology*. Apr 2014;62(4):495-7. doi:10.4103/0301-4738.116461
460. Shindo S, Hirano T, Ueda A, Maeda Y, Ando Y. [A case of cerebral embolism caused by Cardiobacterium hominis endocarditis]. *Rinsho shinkeigaku = Clinical neurology*. 2013;53(8):654-7. doi:10.5692/clinicalneurol.53.654
461. Lopez-Oliva I, Paropkari AD, Saraswat S, et al. Dysbiotic Subgingival Microbial Communities in Periodontally Healthy Patients With Rheumatoid Arthritis. *Arthritis & rheumatology (Hoboken, NJ)*. Jul 2018;70(7):1008-1013. doi:10.1002/art.40485
462. Lee LW, Lee YL, Hsiao SH, Lin HP. Bacteria in the apical root canals of teeth with apical periodontitis. *Journal of the Formosan Medical Association = Taiwan yi zhi*. Jun 2017;116(6):448-456. doi:10.1016/j.jfma.2016.08.010
463. Rôças IN, Siqueira JF, Jr. Species-directed 16S rRNA gene nested PCR detection of Olsenella species in association with endodontic diseases. *Letters in applied microbiology*. 2005;41(1):12-6. doi:10.1111/j.1472-765X.2005.01723.x
464. Rôças IN, Siqueira JF, Jr. Frequency and levels of candidate endodontic pathogens in acute apical abscesses as compared to asymptomatic apical periodontitis. *PloS one*. 2018;13(1):e0190469. doi:10.1371/journal.pone.0190469
465. Tatikonda A, Sudheep N, Biswas KP, Gowtham K, Pujari S, Singh P. Evaluation of Bacteriological Profile in the Apical Root Segment of the Patients with Primary Apical Periodontitis. *The journal of contemporary dental practice*. Jan 1 2017;18(1):44-48. doi:10.5005/jp-journals-10024-1986
466. Vieira Colombo AP, Magalhães CB, Hartenbach FA, Martins do Souto R, Maciel da Silva-Boghossian C. Periodontal-disease-associated biofilm: A reservoir for pathogens of medical importance. *Microbial pathogenesis*. May 2016;94:27-34. doi:10.1016/j.micpath.2015.09.009
467. Wang Y, Wang S, Wu C, et al. Oral Microbiome Alterations Associated with Early Childhood Caries Highlight the Importance of Carbohydrate Metabolic Activities. *mSystems*. Nov 5 2019;4(6)doi:10.1128/mSystems.00450-19
468. Willmann C, Mata X, Hanghoej K, et al. Oral health status in historic population: Macroscopic and metagenomic evidence. *PloS one*. 2018;13(5):e0196482. doi:10.1371/journal.pone.0196482
469. Zakaria MN, Takeshita T, Shibata Y, et al. Microbial community in persistent apical periodontitis: a 16S rRNA gene clone library analysis. *International endodontic journal*. Aug 2015;48(8):717-28. doi:10.1111/iej.12361

470. Joron C, Roméo B, Le Flèche-Matéos A, Rames C, El Samad Y, Hamdad F. Dermacoccus nishinomiyaensis as a cause of persistent paediatric catheter-related bacteraemia. *Clinical microbiology and infection : the official publication of the European Society of Clinical Microbiology and Infectious Diseases*. Aug 2019;25(8):1054-1055. doi:10.1016/j.cmi.2019.02.023
471. Sakamoto M, Takeuchi Y, Umeda M, Ishikawa I, Benno Y, Nakase T. Detection of Treponema socranskii associated with human periodontitis by PCR. *Microbiol Immunol*. 1999;43(5):485-90. doi:10.1111/j.1348-0421.1999.tb02432.x
472. Siqueira JF, Jr., Rocas IN. Treponema socranskii in primary endodontic infections as detected by nested PCR. *J Endod*. Apr 2003;29(4):244-7. doi:10.1097/00004770-200304000-00003
473. Amano J, Hase R, Otsuka Y, Tsuchimochi T, Noguchi Y, Igarashi S. Catheter-related bloodstream infection by Microbacterium paraoxydans in a pediatric patient with B-cell precursor acute lymphocytic leukemia: A case report and review of literature on Microbacterium bacteremia. *J Infect Chemother*. Oct 2019;25(10):806-810. doi:10.1016/j.jiac.2019.03.013
474. Choi HS, Bae EH, Ma SK, Kim SW. Peritoneal Dialysis-Related Peritonitis Caused by Microbacterium paraoxydans. *Jpn J Infect Dis*. Mar 24 2017;70(2):195-196. doi:10.7883/yoken.JJID.2016.004
475. Drobeniuc A, Traenkner J, Rebolledo PA, Ghazaryan V, Rouphael N. Staphylococcus simulans: A rare uropathogen. *IDCases*. 2021;25:e01202. doi:10.1016/j.idcr.2021.e01202
476. Shields BE, Tschetter AJ, Wanat KA. Staphylococcus simulans: An emerging cutaneous pathogen. *JAAD Case Rep*. Nov 2016;2(6):428-429. doi:10.1016/j.jdcr.2016.08.015
477. Goda K, Kenzaka T, Hoshijima M, Yachie A, Akita H. Toxic shock syndrome with a cytokine storm caused by Staphylococcus simulans: a case report. *BMC Infect Dis*. Jan 6 2021;21(1):19. doi:10.1186/s12879-020-05731-y
478. Mallet M, Loiez C, Melliez H, Yazdanpanah Y, Senneville E, Lemaire X. Staphylococcus simulans as an authentic pathogenic agent of osteoarticular infections. *Infection*. Oct 2011;39(5):473-6. doi:10.1007/s15010-011-0173-x
479. Li X, Chen J. Septic Shock Induced by Bacterial Prostatitis with Morganella morganii subsp. morganii in a Posttransplantation Patient. *Case Rep Transplant*. 2015;2015:850532. doi:10.1155/2015/850532
480. Tan JY, Zhang YK, Wu MZ, Yuan CL. [A case of septic shock and multiple organ injury induced by urinary tract infection with Morganella morganii subsp. morganii]. *Zhonghua Wei Zhong Bing Ji Jiu Yi Xue*. Sep 2013;25(9):565. doi:10.3760/cma. j. issn.2095-4352. 2013. 09.020
481. Kawasuji H, Kaya H, Kawamura T, et al. Bacteremia caused by Slackia exigua: A report of two cases and literature review. *J Infect Chemother*. Jan 2020;26(1):119-123. doi:10.1016/j.jiac.2019.06.006
482. Rieber H, Frontzek A, Schmitt H. Slackia exigua, an anaerobic Gram-positive rod and part of human oral microbiota associated with periprosthetic joint infection of the

hip. First case and review of the literature. *Anaerobe*. Apr 2019;56:130-132. doi:10.1016/j.anaerobe.2019.02.015
483. Lee MY, Kim MH, Lee WI, Kang SY. Septic shock caused by Slackia exigua in a patient with diabetes. *Anaerobe*. Dec 5 2021;73:102498. doi:10.1016/j.anaerobe.2021.102498
484. Kus NJ, Kim BJ, Ross HM. A case report of necrotizing fasciitis with growth of Actinomyces europaeus and Actinotignum schaalii. *J Surg Case Rep*. Oct 2019;2019(10):rjz286. doi:10.1093/jscr/rjz286
485. Nielsen HL. First report of Actinomyces europaeus bacteraemia result from a breast abscess in a 53-year-old man. *New Microbes New Infect*. Sep 2015;7:21-2. doi:10.1016/j.nmni.2015.05.001
486. White SE, Woolley SD. Actinomyces europaeus Isolated from a Breast Abscess in a Penicillin-Allergic Patient. *Case Rep Infect Dis*. 2018;2018:6708614. doi:10.1155/2018/6708614
487. Koizumi Y, Ohno T, Takada T, et al. An unexpected case of Cardiobacterium valvarum prosthetic arthritis without cardiac lesions: Case report and literature review. *J Infect Chemother*. May 2021;27(5):747-750. doi:10.1016/j.jiac.2020.12.006
488. Washio Y, Sakamoto SI, Saito R, et al. Infective endocarditis caused by Cardiobacterium valvarum. *Access Microbiol*. 2019;1(8):e000040. doi:10.1099/acmi.0.000040
489. Naim Ur R, Abdullah AK, Hawass NE, Sadiq S, el-Nageeb S, Akhtar Uz Z. Cranial and epidural mycetoma caused by streptomyces somaliensis. *Neuroradiology*. 1987;29(1):95-7. doi:10.1007/BF00341050
490. Mnif B, Boujelbene I, Mahjoubi F, et al. Endocarditis due to Kytococcus schroeteri: case report and review of the literature. *J Clin Microbiol*. Mar 2006;44(3):1187-9. doi:10.1128/JCM.44.3.1187-1189.2006
491. Jourdain S, Miendje Deyi VY, Musampa K, et al. Kytococcus schroeteri infection of a ventriculoperitoneal shunt in a child. *Int J Infect Dis*. Jul 2009;13(4):e153-5. doi:10.1016/j.ijid.2008.09.004
492. Blennow O, Westling K, Froding I, Ozenci V. Pneumonia and bacteremia due to Kytococcus schroeteri. *J Clin Microbiol*. Feb 2012;50(2):522-4. doi:10.1128/JCM.01245-11
493. Kjaer Hansen S, Lofberg SV, Nielsen DK, Kobbero H, Justesen US. Bacteraemia with Moryella indoligenes and Fastidiosipila sanguinis: a case report. *Access Microbiol*. 2020;2(5):acmi000108. doi:10.1099/acmi.0.000108
494. Cheng Z, Huang Y, Wie W, Wang Y, Wang Z. Bloodstream Infection Caused by Bacteroides caccae in a Diabetic Patient: a Case Report and Review of the Literature. *Clin Lab*. Dec 1 2019;65(12)doi:10.7754/Clin.Lab.2019.190534
495. Cobo F, Borrego J, Rojo MD, Navarro-Mari JM. Polymicrobial anaerobic bacteremia due to Atopobium rimae and Parvimonas micra in a patient with cancer. *Anaerobe*. Dec 2018;54:260-263. doi:10.1016/j.anaerobe.2018.02.002
496. George N, Flamiatos E, Kawasaki K, et al. Oral microbiota species in acute apical endodontic abscesses. *J Oral Microbiol*. 2016;8:30989. doi:10.3402/jom.v8.30989

497. Rocas IN, Siqueira JF, Jr. Occurrence of two newly named oral treponemes - Treponema parvum and Treponema putidum - in primary endodontic infections. *Oral Microbiol Immunol*. Dec 2005;20(6):372-5. doi:10.1111/j.1399-302X.2005.00238.x
498. Sayers G, Marques PX, Evans NJ, et al. Identification of spirochetes associated with contagious ovine digital dermatitis. *J Clin Microbiol*. Apr 2009;47(4):1199-201. doi:10.1128/JCM.01934-08
499. Evans NJ, Brown JM, Demirkan I, et al. Three unique groups of spirochetes isolated from digital dermatitis lesions in UK cattle. *Vet Microbiol*. Jul 27 2008;130(1-2):141-50. doi:10.1016/j.vetmic.2007.12.019
500. Cassir N, Benamar S, La Scola B. Clostridium butyricum: from beneficial to a new emerging pathogen. *Clinical microbiology and infection : the official publication of the European Society of Clinical Microbiology and Infectious Diseases*. Jan 2016;22(1):37-45. doi:10.1016/j.cmi.2015.10.014
501. Ewa AU, Ochang EA, Bassey GE, Ekeng BE. Tatumella ptyseos septicaemia in a tertiary hospital in Nigeria: a case report. *Pan Afr Med J*. 2021;39:6. doi:10.11604/pamj.2021.39.6.25490
502. Lee SH, Kim KK, Choi BK. Upregulation of intercellular adhesion molecule 1 and proinflammatory cytokines by the major surface proteins of Treponema maltophilum and Treponema lecithinolyticum, the phylogenetic group IV oral spirochetes associated with periodontitis and endodontic infections. *Infect Immun*. Jan 2005;73(1):268-76. doi:10.1128/IAI.73.1.268-276.2005
503. Rams TE, Hawley CE, Whitaker EJ, Degener JE, van Winkelhoff AJ. Centipeda periodontii in human periodontitis. *Odontology*. Sep 2015;103(3):286-91. doi:10.1007/s10266-014-0166-1
504. Guner Ozenen G, Sahbudak Bal Z, Bilen NM, et al. The First Report of Sphingomonas yanoikuyae as a Human Pathogen in a Child With a Central Nervous System Infection. *The Pediatric infectious disease journal*. Dec 1 2021;40(12):e524. doi:10.1097/inf.0000000000003301
505. Ryan MP, Pembroke JT, Adley CC. Differentiating the growing nosocomial infectious threats Ralstonia pickettii and Ralstonia insidiosa. *European journal of clinical microbiology & infectious diseases : official publication of the European Society of Clinical Microbiology*. Oct 2011;30(10):1245-7. doi:10.1007/s10096-011-1219-9
506. Bernshteyn M, Kumar PA, Joshi S. Kocuria kristinae pneumonia and bacteremia. *Proceedings (Baylor University Medical Center)*. Jul 23 2020;33(4):608-609. doi:10.1080/08998280.2020.1792749
507. Kate A, Joseph J, Bagga B. Kocuria kristinae interface keratitis following deep anterior lamellar keratoplasty. *Indian journal of ophthalmology*. Jul 2020;68(7):1463-1466. doi:10.4103/ijo.IJO_1455_19
508. Pandit L, Cox LM, Malli C, et al. Clostridium bolteae is elevated in neuromyelitis optica spectrum disorder in India and shares sequence similarity with AQP4. *Neurology(R) neuroimmunology & neuroinflammation*. Jan 2021;8(1)doi:10.1212/nxi.0000000000000907
509. Pequegnat B, Sagermann M, Valliani M, et al. A vaccine and diagnostic target for Clostridium bolteae, an autism-associated bacterium. *Vaccine*. Jun 10 2013;31(26):2787-

90. doi:10.1016/j.vaccine.2013.04.018

510. Li J, Chen P, Li J, Gao X, Chen X, Chen J. A new treatment of sepsis caused by veillonella parvula: A case report and literature review. *Journal of clinical pharmacy and therapeutics*. Oct 2017;42(5):649-652. doi:10.1111/jcpt.12559

511. Bhatti MA, Frank MO. Veillonella parvula meningitis: case report and review of Veillonella infections. *Clinical infectious diseases : an official publication of the Infectious Diseases Society of America*. Sep 2000;31(3):839-40. doi:10.1086/314046

512. Collins M, Hutson RA, Falsen E, Sjöden B, Facklam RR. Description of Gemella sanguinis sp. nov., isolated from human clinical specimens. *Journal of clinical microbiology*. 1998;36(10):3090-3.

513. Zbinden A, Mueller NJ, Tarr PE, Spröer C, Keller PM, Bloemberg GV. Streptococcus tigurinus sp. nov., isolated from blood of patients with endocarditis, meningitis and spondylodiscitis. *International journal of systematic and evolutionary microbiology*. Dec 2012;62(Pt 12):2941-2945. doi:10.1099/ijs.0.038299-0

514. Zbinden A, Bostanci N, Belibasakis GN. The novel species Streptococcus tigurinus and its association with oral infection. *Virulence*. 2015;6(3):177-82. doi:10.4161/21505594.2014.970472

515. Gahl M, Stöckli T, Fahrner R. Facklamia hominis bacteremia after transurethral resection of the prostate: a case report. *BMC urology*. Dec 7 2020;20(1):192. doi:10.1186/s12894-020-00762-8
