# Supplementary material for: The species-level microbiota of healthy eyes revealed by the integration of metataxonomics with culturomics and genome analysis
Source: Front Microbiol. 2022 Sep 2;13:950591. doi: 10.3389/fmicb.2022.950591 (PMC9481467; doi:10.3389/fmicb.2022.950591)
Supplement: Supplementary Table 3 — Statistical table of pathogenicity analysis of strains isolated and cultivated on the ocular surface. [file Table_3.PDF]

Supplementary Table 3*:* Statistical table of pathogenicity analysis of strains isolated and cultivated on the ocular surface

Serial number	Species	Genus	Number of isolated bacteria	Pathogenicity analysis
1	*Aeromicrobium_choanae*	*Aeromicrobium*	1	
2	*Aeromicrobium_flavum*	*Aeromicrobium*	1	
3	*Arthrobacter_citreus*	*Aeromicrobium*	1	
4	*Bacillus_cereus*	*Bacillus*	1	Can cause food poisoning [1]
5	*Bacillus_pumilus*	*Bacillus*	26	
6	*Bacillus_subtilis*	*Bacillus*	1	
7	*Bacillus_velezensis*	*Bacillus*	4	
8	*Bifidobacterium_dentium*	*Bifidobacterium*	1	Related to oral diseases [2]
9	*Brevundimonas_vesicularis*	*Brevundimonas*	28	
10	*Corynebacterium_simulans*	*Corynebacterium*	2	Acute suppurative spondylitis that can be caused [3]; Cause pneumonia [4]
11	*Corynebacterium_tuberculostearicum*	*Corynebacterium*	8	Ubiquitous bacteria on human skin that can cause skin diseases [5]
12	*Cutibacterium_avidum*	*Cutibacterium*	3	Can cause skin and soft tissue infections and is regarded as a potential skin pathogen [6]
13	*Cutibacterium_acnes*	*Cutibacterium*	1	Can cause skin diseases, is the main bacteria causing acne [7]
14	*Enterococcus_hirae*	*Enterococcus*	14	Bacteremia [8]; Endocarditis [9]; Acute osteomyelitis [10]; Urinary tract infection [11]
15	*Georgenia_daeguensis*	*Georgenia*	1	
16	*Microbacterium_oleivorans*	*Microbacterium*	30	
17	*Micrococcus_aloeverae*	*Micrococcus*	3	Peritonitis [12]
18	*Micrococcus_antarcticus*	*Micrococcus*	4	
19	*Micrococcus_endophyticus*	*Micrococcus*	3	

20	*Micrococcus_yunnanensis*	*Micrococcus*	11	
21	*Moraxella_osloensis*	*Moraxella*	25	Peritonitis [13] ;Bacteremia [14]
22	*Paenibacillus_silvae*	*Paenibacillus*	10	
23	*Paracoccus_laeviglucosivorans*	*Paracoccus*	4	
24	*Parvimonas_micra*	*Parvimonas*	6	Bacteremia [15]
25	*Prevotella_intermedia*	*Prevotella*	1	Related to oral cancer [16]
26	*Pseudomonas_fragi*	*Pseudomonas*	1	
27	*Pseudomonas_frederiksbergensis*	*Pseudomonas*	4	
28	*Pseudomonas_koreensis*	*Pseudomonas*	3	
29	*Pseudomonas_orientalis*	*Pseudomonas*	1	
30	*Pseudomonas_weihenstephanensis*	*Pseudomonas*	2	
31	*Slackia_exigua*	*Slackia*	2	Related to periodontal disease [17] ;Bacteremia [18]
32	*Sphingomonas _olei*	*Sphingomonas*	1	
33	*Staphylococcus_aureus*	*Staphylococcus*	2	Can cause pneumonia, otitis media and septicemia [19]
34	*Staphylococcus_capitis*	*Staphylococcus*	1	Cause endocarditis [20]; Skin and soft tissue infection [21]; Osteomyelitis [22]; Artificial joint infection [23]
35	*Staphylococcus_epidermidis*	*Staphylococcus*	168	Bacteremia [24]
36	*Staphylococcus_haemolyticus*	*Staphylococcus*	23	Meningitis [25]; Testicular epididymitis and bacteremia [26]
37	*Staphylococcus_hominis*	*Staphylococcus*	2	Skin and soft tissue infection [27]; Cellulitis and bacteremia [28-30]
38	*Staphylococcus_warneri*	*Staphylococcus*	20	
39	*Streptococcus _cristatus*	*Staphylococcus*	1	Neonatal septic arthritis [31]
40	*Streptococcus _pseudopneumoniae*	*Staphylococcus*	2	Cystic fibrosis [32]
41	*Streptococcus_oralis*	*Staphylococcus*	3	Peritonitis [33]
42	*Terribacillus_aidingensis*	*Terribacillus*	2	

Table Note: 20 of the 42 strains isolated and cultured in this study are potential pathogenic bacteria, including Moraxella. It has been reported that Moraxella can cause keratitis, conjunctivitis and endophthalmitis, and is an important bacterial pathogen of eyes [34-35].

REFERCENS

[1] Ho D, Ang G, Er C, Yap SF, Meyyur Aravamudan V. An Unusual Presentation of Parvimonas micra Infective Endocarditis. Cureus. 2018;10(10):e3447. Published 2018 Oct 13. doi:10.7759/cureus.3447

[2] Pokusaeva K, Johnson C, Luk B, et al. GABA-producing Bifidobacterium dentium modulates visceral sensitivity in the intestine. Neurogastroenterol Motil. 2017;29(1):e12904. doi:10.1111/nmo.12904

[3] Ogasawara M, Matsuhisa T, Kondo T, et al. Pyogenic spondylitis with acute course caused by Corynebacterium simulans. J Infect Chemother. 2020;26(3):294-297. doi:10.1016/j.jiac.2019.10.012

[4] Online Microbiology Notes https://microbenotes.com/ 2020-3-30

[5] Altonsy MO, Kurwa HA, Lauzon GJ, et al. Corynebacterium tuberculostearicum, a human skin colonizer, induces the canonical nuclear factor-κB inflammatory signaling pathway in human skin cells. Immun Inflamm Dis. 2020;8(1):62-79. doi:10.1002/iid3.284

[6] Corvec S. Clinical and Biological Features of Cutibacterium (Formerly Propionibacterium) avidum, an Underrecognized Microorganism. Clin Microbiol Rev. 2018;31(3):e00064-17. Published 2018 May 30. doi:10.1128/CMR.00064-17

[7] Boisrenoult P. Cutibacterium acnes prosthetic joint infection: Diagnosis and treatment. Orthop Traumatol Surg Res. 2018;104(1S):S19-S24. doi:10.1016/j.otsr.2017.05.030

[8] Nakamura T, Ishikawa K, Matsuo T, Kawai F, Uehara Y, Mori N. Enterococcus hirae bacteremia associated with acute pyelonephritis in a patient with alcoholic cirrhosis: a case report and literature review. BMC Infect Dis. 2021;21(1):999. Published 2021 Sep 23. doi:10.1186/s12879-021-06707-2

[9] Pinkes ME, White C, Wong CS. Native-valve Enterococcus hirae endocarditis: a case report and review of the literature. BMC Infect Dis. 2019;19(1):891. Published 2019 Oct 24. doi:10.1186/s12879-019-4532-z

[10] Bollam R, Yassin M, Phan T. Detection of Enterococcus hirae in a case of acute osteomyelitis. Radiol Case Rep. 2021;16(9):2366-2369. Published 2021 Jul 1. doi:10.1016/j.radcr.2021.06.016

[11] Bilek HC, Deveci A, Ünal S, Tanrıverdi Çaycı Y, Tanyel E. Enterococcus hirae as a cause of bacteremic urinary tract infection: first case report from Turkey. J Infect Dev Ctries. 2020;14(12):1780-1482. Published 2020 Dec 31. doi:10.3855/jidc.12522

[12] Song SH, Choi HS, Ma SK, Kim SW, Shin JH, Bae EH. Micrococcus aloeverae - A Rare Cause of Peritoneal Dialysis-Related Peritonitis Confirmed by 16S rRNA Gene Sequencing. J Nippon Med Sch. 2019;86(1):55-57. doi:10.1272/jnms.JNMS.2019_86-10

[13] Yamada A, Kasahara K, Ogawa Y, et al. Peritonitis due to Moraxella osloensis: A case report and literature review. J Infect Chemother. 2019;25(12):1050-1052. doi:10.1016/j.jiac.2019.05.018

[14] Maruyama Y, Shigemura T, Aoyama K, Nagano N, Nakazawa Y. Bacteremia due to Moraxella osloensis: a case report and literature review. Braz J Infect Dis. 2018;22(1):60-62. doi:10.1016/j.bjid.2017.10.008

[15] Alonso A, Rojo F, Martínez JL. Environmental and clinical isolates of Pseudomonas aeruginosa show pathogenic and biodegradative properties irrespective of their origin. Environ Microbiol. 1999;1(5):421-430. doi:10.1046/j.1462-2920.1999.00052.x

[16] Zhang L, Liu Y, Zheng HJ, Zhang CP. The Oral Microbiota May Have Influence on Oral Cancer. Front Cell Infect Microbiol. 2020;9:476. Published 2020 Jan 15. doi:10.3389/fcimb.2019.00476

[17] Rieber H, Frontzek A, Schmitt H. Slackia exigua, an anaerobic Gram-positive rod and part of human oral microbiota associated with periprosthetic joint infection of the hip. First case and review of the literature. Anaerobe. 2019;56:130-132. doi:10.1016/j. anaerobe.2019.02.015

[18] Lim KR, Son JS, Moon SY. A case of Slackia exigua bacteremia associated with pyometra in a patient with poor dentition [published online ahead of print, 2021 Nov 12]. Anaerobe. 2021;73:102477. doi:10.1016/j.anaerobe.2021.102477

[19] Peetermans M, Meyers S, Liesenborghs L, et al. Von Willebrand factor and ADAMTS13 impact on the outcome of Staphylococcus aureus sepsis. J Thromb Haemost. 2020;18(3):722-731. doi:10.1111/jth.14686

[20] Thakker RA, Chatila K, Reynoso D, Karnath B. Native and Prosthetic Valve Staphylococcus capitis Endocarditis: A Review of the Literature. Cardiol Res. 2021;12(3):140-145. doi:10.14740/cr1231

[21] Natsis NE, Cohen PR. Coagulase-Negative Staphylococcus Skin and Soft Tissue Infections. Am J Clin Dermatol. 2018;19(5):671-677. doi:10.1007/s40257-018-0362-9

[22] Brooks D, Thomas V, Snowden J. Staphylococcus capitis Osteomyelitis: Case Report. Glob Pediatr Health. 2019;6:2333794X19833736. Published 2019 Mar 7. doi:10.1177/2333794X19833736

[23] Tevell S, Hellmark B, Nilsdotter-Augustinsson Å, Söderquist B. Staphylococcus capitis isolated from prosthetic joint infections. Eur J Clin Microbiol Infect Dis. 2017;36(1):115-122. doi:10.1007/s10096-016-2777-7

[24] Ponce de Leon S, Wenzel RP. Hospital-acquired bloodstream infections with Staphylococcus epidermidis. Review of 100 cases. Am J Med. 1984;77(4):639-644. doi:10.1016/0002-9343(84)90354-1

[25] Bryce AN, Doocey R, Handy R. Staphylococcus haemolyticus meningitis and bacteremia in an allogenic stem cell transplant patient. IDCases. 2021;26:e01259. Published 2021 Aug 24. doi:10.1016/j.idcr.2021.e01259

[26] Pindar C, Viau RA. Staphylococcus haemolyticus epididymo-orchitis and bacteraemia: a case report. JMM Case Rep. 2018;5(7):e005157. Published 2018 Jul 5. doi:10.1099/jmmcr.0.005157

[27] Natsis NE, Cohen PR. Coagulase-Negative Staphylococcus Skin and Soft Tissue Infections. Am J Clin Dermatol. 2018;19(5):671-677. doi:10.1007/s40257-018-0362-9

[28] Uddin O, Hurst J, Alkayali T, Schmalzle SA. Staphylococcus hominis cellulitis and bacteremia associated with surgical clips. IDCases. 2022;27:e01436. Published 2022 Feb 1. doi:10.1016/j.idcr.2022.e01436

[29] Ahmed NH, Baruah FK, Grover RK. Staphylococcus hominis subsp. novobiosepticus, an emerging multidrug-resistant bacterium, as a causative agent of septicaemia in cancer patients. Indian J Med Res. 2017;146(3):420-425. doi:10.4103/ijmr.IJMR_1362_15

[30] Dansey K, Stratton L, Park BD. Staphylococcus hominis carotid artery infection with septic embolization. J Vasc Surg Cases. 2015;1(2):81-83. Published 2015 Apr 21. doi:10.1016/j.jvsc.2015.03.001

[31] Gupta G, Chaudhary M, Khunt A, Shah V, Shah MM. An unreported case of Streptococcus cristatus septic arthritis of wrist in a neonate. J Clin Orthop Trauma. 2020;11(2):328-331. doi:10.1016/j.jcot.2019.02.002

[32] Alonso A, Rojo F, Martínez JL. Environmental and clinical isolates of Pseudomonas aeruginosa show pathogenic and biodegradative properties irrespective of their origin. Environ Microbiol. 1999;1(5):421-430. doi:10.1046/j.1462-2920.1999.00052.x

[33] Kotani A, Oda Y, Hirakawa Y, Nakamura M, Hamasaki Y, Nangaku M. Peritoneal Dialysis-Related Peritonitis Caused by Streptococcus oralis. Intern Med. 2021;60(21):3447-3452. doi:10.2169/internalmedicine.6234-20

[34] Walls A, Wald E. Neonatal Moraxella osloensis ophthalmia. Emerg Infect Dis. 2005;11(11):1803-1804. doi:10.3201/eid1111.050488

[35] Berrocal AM, Scott IU, Miller D, Flynn HW Jr. Endophthalmitis caused by Moraxella species. Am J Ophthalmol. 2001;132(5):788-790. doi:10.1016/s0002-9394(01)01098-4
